# Extreme expansion of NBS-encoding genes in *Rosaceae*

**DOI:** 10.1186/s12863-015-0208-x

**Published:** 2015-05-03

**Authors:** YanXiao Jia, Yang Yuan, Yanchun Zhang, Sihai Yang, Xiaohui Zhang

**Affiliations:** State Key Laboratory of Pharmaceutical Biotechnology, School of Life Sciences, Nanjing University, 210023 Nanjing, China

**Keywords:** NBS-encoding gene, Rosaceae, Cucurbitaceae, Expansion, Rapid evolution

## Abstract

**Background:**

Nucleotide binding site leucine-rich repeats (*NBS-LRR*) genes encode a large class of disease resistance (R) proteins in plants. Extensive studies have been carried out to identify and investigate NBS-encoding gene families in many important plant species. However, no comprehensive research into NBS-encoding genes in the Rosaceae has been performed.

**Results:**

In this study, five whole-genome sequenced Rosaceae species, including apple, pear, peach, mei, and strawberry, were analyzed to investigate the evolutionary pattern of NBS-encoding genes and to compare them to those of three Cucurbitaceae species, cucumber, melon, and watermelon. Considerable differences in the copy number of NBS-encoding genes were observed between Cucurbitaceae and Rosaceae species. In Rosaceae species, a large number and a high proportion of NBS-encoding genes were observed in peach (437, 1.52%), mei (475, 1.51%), strawberry (346, 1.05%) and pear (617, 1.44%), and apple contained a whopping 1303 (2.05%) NBS-encoding genes, which might be the highest number of *R*-genes in all of these reported diploid plant. However, no more than 100 NBS-encoding genes were identified in Cucurbitaceae. Many more species-specific gene families were classified and detected with the signature of positive selection in Rosaceae species, especially in the apple genome.

**Conclusions:**

Taken together, our findings indicate that NBS-encoding genes in Rosaceae, especially in apple, have undergone extreme expansion and rapid adaptive evolution. Useful information was provided for further research on the evolutionary mode of disease resistance genes in Rosaceae crops.

**Electronic supplementary material:**

The online version of this article (doi:10.1186/s12863-015-0208-x) contains supplementary material, which is available to authorized users.

## Background

The battle between plants and pathogens has gone on since they first emerged in the Earth’s ecosystem. This ongoing battle against pathogens has led to two types of immune responses in plants: a basal response to pathogen-associated molecular patterns (PAMPs) and a gene-for-gene response specific to a pathogen [1-4]. The former is present constitutively and the latter is induced upon exposure to pathogens. The latter, which is mediated by plant resistance (*R*) genes, is better studied. Plants have *R* genes whose products can recognize the complementary avirulence genes of pathogens. This defense mechanism has aroused people’s great interest, because it is possible to exploit the natural inducible defenses to engineer broad-spectrum pathogen resistance. It will be of great significance in crop breeding.

Numerous *R* genes from many plants have been cloned and characterized over the past few decades. Most cloned *R* genes belong to a large gene family. In this family, the genes encode proteins with nucleotide binding sites and leucine rich repeats (NBS-LRR) domain [5]. Moreover, according to the N-terminal of proteins, the *NBS-LRR* gene family can be further classified into two types, *TIR-NBS-LRR* (TIR) genes with a Toll/Interleukin-1 (TIR) receptor domain and non-*TIR-NBS-LRR* (non-TNL) genes that lack the TIR domain. These often have a coiled-coil (CC) domain instead [6,7].

When a genome sequence is available, the analysis of large gene families is helpful to understand the major events responsible for their molecular evolution. In recent years, lots of plant species have been whole-genome sequenced and these provide abundant materials for investigating the evolutionary patterns of *R* genes. Studies of the NBS gene family has been performed in many monocots and dicots, such as *Oryza sativa*, *Zea mays*, *Populus trichocarpa*, *Malus domestica*, *Arabidopsis thaliana*, *Brassica rapa*, *Citrus sinensis*, and *Solanum tuberosum* [8-18]. All the results have shown that the size of the NBS gene family differs in each species. In general, approximately 0.2–1.6% of genes predicted in plant genomes are NBS-encoding genes. They also have diverse evolutionary characters. Frequent gene duplications and gene loss of NBS-encoding genes in different species have been observed, indicating a rapid evolution of this gene family.

A few studies focused on comparative analysis of NBS genes among closely related species provide more information that can be used to assess the evolutionary process and identify unique and identical evolutionary patterns of *R* genes. Comparative analysis of *NBS-LRR* genes in four gramineous species, rice, maize, sorghum, and brachypodium, showed considerable copy number variation and a tendency of gene loss in grass species [19]. Similarly, Luo et al. also investigated the *R* genes in four Poaceae species and observed frequent deletions and translocations [20]. A survey of *R* genes in different Cucurbitaceae species has indicated that Cucurbitaceae species harbor a limited number of *R* gens. It can be inferred that the reasons for the low copy number of *R* genes are frequent loss and infrequent duplications [21]. Recently, four species of the legume family, including *Medicago truncatula*, soybean, common bean, and pigeon pea, also have been studied in genome-wide to investigate the *NBS-LRR* genes [12]. This study indicated differential NBS gene loss and frequent duplications during legume evolution and ectopic duplications were supposed to create many novel NBS gene loci in individual legume genomes.

As more genomic data have been available for some angiosperm families, *NBS-LRR* genes should be further investigated among phylogenetically similar related species to fill the gaps in the understanding of their evolutionary patterns. The Rosids comprise a very large group of eudicots, containing 16 orders and splitting between the Fabids (Euroside I) and the Malvids (Euroside II). The Fabids contains many plants of great agricultural importance, such as members of the Rosaceae, Cucurbitaceae and Fabaceae. Rosaceae comprises approximately 3400 species and it grows throughout the world. The family is important, because it includes many economically important genera such as *Malus* (apples), *Pyrus* (pears), *Prunus* (plums, cherries, almonds, apricots), *Rubus* (raspberries, blackberries), and *Fragaria* (strawberries). The rose family is also a source of ornamental plants. The Rosaceae constantly face threats from various pathogens, including bacteria, fungi, nematodes, and viruses. However, few functional *R* genes in Rosaceae have been identified and cloned [22,23]. Therefore, it would be interesting to investigate the *R* gene repertoire among different Rosaceae species. The gourd family (Cucurbitaceae) also contains many useful species of food and ornamental plants. It includes the gourds, melons, squashes, and pumpkins. Like other plants, the gourd family also faces an extensive damage in productivity because of lots of diseases. It is reported that *NBS* genes in Rosaceae have experienced expansion and more than 1000 *NBS-LRRs* have been detected in apple [8,24], whereas Cucurbitaceae species have been found to contain a limited number of *R* genes (<100) [21]. The distinct features of *R* genes in the two families of Euroside I provide an interesting topic for comparing and uncovering the different evolutionary patterns of *R* genes in the two families of Rosids.

Here, five genomes of representative species in various Rosaceae genera, *Prunus persica*, *Prunus mume*, *Fragaria vesca*, *Pyrus bretschneideri Rehd,* and *Malus domestica*, were used for a comprehensive analysis of *R* genes [24-28]. Meanwhile, we reannotated *R* genes from three sequenced genomes in the gourd family, including *Cucumis sativus*, *Cucumis melo*, and *Citrullus lanatus* [29-32]. A comparative analysis of *R* genes was performed between Rosaceae and Cucurbitaceae. Considerable copy number variations of NBS-encoding genes were observed between the Cucurbitaceae and Rosaceae species. Fewer than 100 NBS-encoding genes were identified in Cucurbitaceae while 346-1303 NBS genes were found in Rosaceae. Many more species-specific gene families were detected in Rosaceae species, especially in the apple genome, suggesting a recent expansion of *R* genes in these genomes. The possible reason for the differentiation in the gene copy number is discussed.

## Results

### Numbers of NBS-encoding gene in different plant genomes

**Figure 1:**
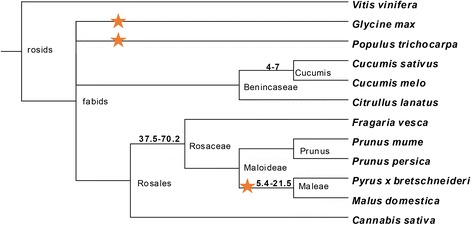
Species tree of plant species used in this study. Stars indicate the occurrence of recent whole genome duplication (WGD). Numbers in the figure indicate species divergence time. Units: MYA (million years ago). The data were downloaded from NCBI Common Tree in the Taxonomy section (http://www.ncbi.nlm.nih.gov/) and the tree was constructed using TreeView.

Eight plant species from Cucurbitaceae and Rosaceae were selected to identify and compare NBS-encoding genes in their genomes (Figure 1). Three of the eight species were from the Cucurbitaceae: cucumber (*Cucumis sativus*), melon (*Cucumis melo*) and watermelon (*Citrullus lanatus*), while the other five were from the Rosaceae, peach (*Prunus persica*), mei (*Prunus mume*), strawberry (*Fragaria vesca*), pear (*Pyrus bretschneideri*) and apple (*Malus domestica*). Additionally, Cannabis (*Cannabis sativa*) was selected as an outer group species of the Rosaceae [33], while poplar (*Populus trichocarpa*), soybean (*Glycine max*) and grape (*Vitis vinifera*) were chosen as examples of the Salicaceae, Leguminosae, and Vitaceae families [34-36].

Different patterns in NBS-encoding gene numbers were observed between Cucurbitaceae and Rosaceae. Fewer than 100 NBS-encoding genes were identified in all of these cucurbitaceous species (Table 1). Three sequenced genomes of cucumber (V1,V2 and VW) had 59, 62, and 71 NBS-encoding genes, respectively, while melon and watermelon contained 80 and 45 NBS-encoding genes, respectively. The proportions of NBS-encoding genes in the whole genome were also low (0.19%–0.27%), which may be the lowest level reported so far, indicating that the cucurbitaceous species may have other mechanisms of disease resistance that reduced their need to have as many NBS-encoding genes as other plants [30,31].

**Table 1:** **NBS-encoding genes among surveyed plant species**

**Family**	**Species**	**Sequenced length (Mb)**	**Genome size (Mb)**	**Estimated gene number**	**Number of NBS-encoding genes**	**Percentage of NBS-encoding genes**	**Reference**
**Cucurbitaceae**	Cucumber V1	243.5	367	26682	59	0.22%	30
	Cucumber V2	203	367	25600	62	0.24%	30
	Cucumber VW	224	367	26548	71	0.27%	32
	Melon	375	450	27427	80	0.23%	29
	Watermelon	353.5	425	23440	45	0.19%	31
**Rosaceae**	Peach	224.6	265	27852	437	1.52%	25
	Mei	237	280	31390	475	1.51%	27
	Strawberry	240	240	34809	346	1.05%	28
	Pear	512	527	42812	617	1.44%	26
	Apple	604	742	57386	1303	2.05%	22
	Cannabis	534	818/843	~30,000	234	0.78%	33
	Poplar	485	550	45654	402	0.88%	35
	Grape	487	475	30434	341	1.12%	34
	Soybean	950	1,115	46430	392	0.84%	36

On the contrary, the rosaceous species had a large number and a high proportion of NBS-encoding genes. Peach, mei, strawberry and pear each had 437, 475, 346, and 617 NBS-encoding genes, while apple even contained 1303 NBS-encoding genes, which might have the highest *R*-gene numbers in all of these reported diploid plants (Table 1). These NBS-encoding genes accounted for about 1.05–2.05%, of all predicted genes in the five rosaceous species. Only 234 NBS-encoding genes were identified in the outgroup, Cannabis, suggesting a common expansion of NBS-encoding genes after the split between cannabis and the ancestor of Rosaceae species. Moreover, NBS-encoding genes in the five Rosaceae species might have different evolutionary patterns after their split from the common ancestors due to that their copy numbers of NBS-encoding genes varied great differently. For example, although pear and apple are both Maloideae species and diverged from each other not long ago, the number of NBS-encoding genes in apple was 2-fold greater than in pear. Meanwhile, pear and apple contained 1.3-3.8 times of NBS genes than their relative species. The other three species, poplar, grape and soybean, which are evolutionarily distant from the Cucurbitaceae and Rosacea in the phylogenetic tree, contained 402, 341, and 392 NBS-encoding genes, respectively.

### Classification of TIR and non-TIR NBS-encoding genes

**Figure 2:**
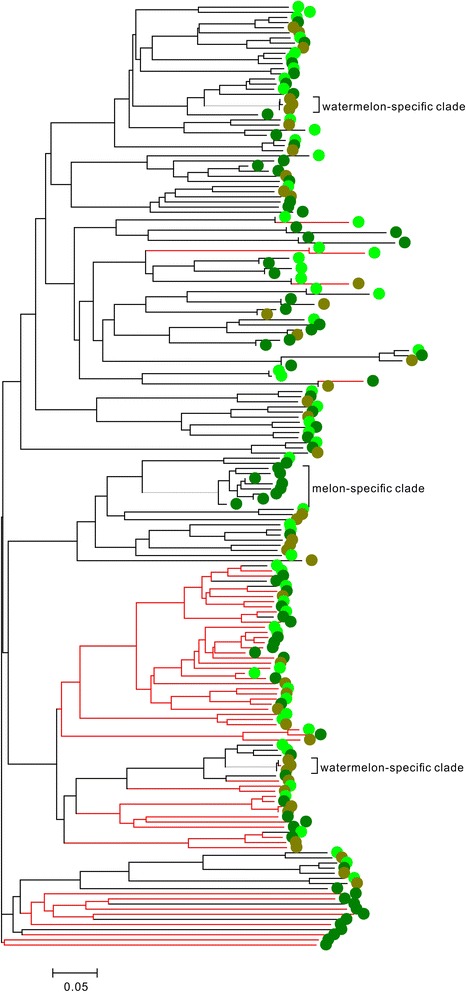
Phylogenetic tree based on NBS domain of NBS-encoding genes in cucumber, melon and watermelon. Red lines represent TIR genes and black lines represent non-TIR genes. NBS-encoding genes from cucumber, melon and watermelon are shown as light green circles, green circles and brown circles, respectively. The brackets denote species-specific gene clades.

The NBS-encoding genes usually can be further classified into two types based on the structures of N-terminus: the TIR subclass and the non-TIR subclass. Based on Pfam results and the phylogenetic tree (Figures 2, Additional file 1: Figure S1, Additional file 2: Figure S2, and Additional file 3: Figure S3), we divided all NBS-encoding genes into TIR and non-TIR NBS-encoding genes. A total of 1705 TIR genes and 1754 non-TIR genes were detected. In general, each genome had similar numbers of TIR genes and non-TIR genes (41% to 55%, Table 2).

**Table 2:** **Numbers of TIR and non-TIR NBS-encoding genes**

**Species**	**TIR genes**					**Non-TIR genes**				
	**XN**	**XNL**	**TN**	**TNL**	**Sum**	**CN**	**CNL**	**XN**	**XNL**	**Sum**
Cucumber.V1	1	9	5	12	**27**	4	12	6	10	**32**
Cucumber.V2	0	8	3	15	**26**	1	16	3	16	**36**
Cucumber.VW	4	6	2	21	**33**	2	17	7	12	**38**
Melon	2	8	4	21	**35**	5	14	12	14	**45**
Watermelon	0	5	3	12	**20**	0	8	7	10	**25**
Peach	10	23	15	133	**181**	9	112	27	101	**249**
Mei	10	27	32	155	**224**	22	104	28	93	**247**
Strawberry	6	15	15	117	**153**	9	94	19	71	**193**
Pear	22	54	25	241	**342**	23	152	24	76	**275**
Apple	88	184	91	301	**664**	83	270	81	205	**639**
**Sum**	**143**	**339**	**195**	**1028**	**1705**	**158**	**799**	**214**	**608**	**1754**

To further classify these TIR genes and non-TIR genes, we categorized them into different groups based on N and C terminal domains. Of the TIR-NBS-encoding genes, four sub-types, TIR-NBS-LRR (TNL), TIR-NBS (TN), X-NBS-LRR (XNL), and X-NBS (XN) were identified (Table 2). Over 60% TIR genes had the LRR domains (1028/1705). In each genome, TNL genes made up the greatest proportion of all genes detected. Similarly, non-TIR genes were also classified into four types, including 158 CC-NBS (CN), 799 CC-NBS-LRR (CNL), 214 X-NBS (XN), and 608 X-NBS-LRR (XNL) (Table 2).

Although the number of TIR and non-TIR genes in each species was almost identical, the average exon number was greatly different (Additional file 4: Table S1). TIR genes were predicted to have 6.2 exons in average, which is significantly larger than the average number of non-TIR genes, 2.9 (t-test, *P* < 0.001). For each plant, the average numbers of TIR exons were 1.5–2.8-fold greater than non-TIRs. This was consistent with the results of a previous study in the Arabidopsis, poplar and grapevine genomes, which may support the idea that CNLs tend to be encoded by a single exon while TNLs gravitate towards multiple exons [9,37]. Results showed both the exon numbers of TIR and non-TIR genes in strawberry were the largest.

### Identification of different types of gene families and genome organization analysis

All NBS-encoding genes were classified into families based on the sequence similarity >60% and coverage >60%. A total of 1006 gene families were identified, including 828 species-specific gene families and 178 multi-species gene families (Tables 3, 4 and 5). Different features of species-specific and multi-species gene families were observed in different species. About 70–100% of species-specific gene families are single gene families. All peach-specific and watermelon-specific gene families contained exactly one member each. The average gene number of species-specific gene families ranged from 1–1.7 (Table 3). The proportions of genes in species-specific gene families focused on and mostly resided in 14.2–31.1%. The proportions of species-specific genes were oddly high in strawberry (84.4%) and cannabis (99.1%). Meanwhile, only 9 and 5 large families (family members ≥5) were identified in strawberry and cannabis, respectively. These results indicate that the two species have a relatively distant relationship with other Rosaceae species and have experienced few recent duplication events.

**Table 3:** **Analysis of species-specific gene families in Cucurbitaceae and Rosaceae**

**Species**	**Number of family**	**Number of genes**	**Average gene member**	**Members of largest family**	**Average nucleotide divergence**	**Ave_** ***Ka/Ks***	PAML (* + **|All)
Cucumber	13	14	1.1	2	0.223	0.519	-
Melon	21	26	1.2	2	0.071	0.772	-
Watermelon	14	14	1	1	-	-	-
Apple	215	287	1.3	12	0.087	0.836	11|13
Peach	63	63	1	1	-	-	-
Pear	72	88	1.2	4	0.099	0.617	3|4
Mei	93	95	1	2	0.119	0.453	-
Strawberry	200	292	1.5	22	0.207	0.667	16|16
Cannabis	137	232	1.7	11	0.148	0.501	12|18

*P < 0.01; **P <0.05.

**Table 4:** **Analysis of multi-species gene families in Cucurbitaceae**

**Type of gene family**	**Average number of members**			**Number of gene family**	**Number of genes**	**Members of largest family**	**Average nucleotide divergence**	**Ave_** ***Ka/Ks***	PAML (* + **|All)
	**C**	**M**	**W**						
CM	1.2	1.3	-	11	27	4	0.424	0.548	4|4
CW	1.0	-	1.0	1	2	2	0.231	0.414	-
MW	-	1.0	1.0	1	2	2	0.148	0.295	-
CMW	1.6	2.1	1.5	19	99	18	0.189	0.397	5|10

C: Cucumber; M: Melon; W: Watermelon.

*P < 0.01; **P <0.05.

**Table 5:** **Analysis of multi-species gene families in Rosaceae**

**Type of gene family**	**Average number of members**						**Number of gene family**	**Number of genes**	**Members of largest family**	**Average nucleotide divergence**	**Ave_** ***Ka/Ks***	PAML (* + **|All)
	**A**	**M**	**Pc**	**Pr**	**S**	**C**						
APc	1	-	1	-	-	-	1	2	2	0.341	0.463	-
APcPr	2	-	1	1	-	-	1	4	4	0.169	0.660	1|1
AMPcPr	17.8	5.6	5.3	9.2	-	-	28	1060	189	0.251	0.525	14|18
AMPcPrS	11.1	5.9	6.3	6.1	3	-	14	453	119	0.269	0.396	7|10
APcPrS	1	-	1	1	1	-	1	4	4	0.157	0.216	0|1
AMPc	1.5	1	1	-	-	-	2	7	4	0.197	0.481	1|2
APr	8.1	-	-	3.5	-	-	37	427	131	0.150	0.649	20|26
AMPr	1.7	1.3	-	1.7	-	-	3	14	6	0.167	0.374	1|3
APrS	49	-	-	45	5	-	1	99	99	0.534	0.577	-
PcPr	-	-	1	1	-	-	1	2	2	0.238	0.534	-
MPcPr	-	5	3.5	1	-	-	2	19	12	0.310	0.529	1|2
MPc	-	2.4	2.5	-	-	-	48	235	36	0.109	0.637	19|20
MPcS	-	2	1.7	-	1	-	3	14	6	0.234	0.499	1|3
MPr	-	1	-	1	-	-	1	2	2	0.228	0.374	-
AMPcPrSC	1	1	1	1	1	1	1	6	6	0.207	0.091	0|1
MPcSC	-	1	1	-	1	1	1	4	4	0.225	0.199	0|1

A: Apple; M: Mei; Pc: Peach; Pr: Pear; S: Strawberry; C: Cannabis.

*P < 0.01; **P <0.05.

For Cucurbitaceae, most lineage-specific families contained genes from all the three species, suggesting that most NBS-encoding genes in multi-species gene families are present in the ancestor and retained after the split of the three species (Table 4). Then, the cucumber-melon-lineage-specific gene families occupied the second largest proportions, far exceeding to the other types. This is consistent with the fact that cucumber is more similar to melon than to watermelon.

Although 16 types of multi-species families were classified in Rosaceae and Cannabis, only 12 types had no more than three families (Table 5). The four main types of gene families are Apple-Mei-Peach-Pear-Strawberry (AMPcPrS, 14), Apple-Mei-Peach-Pear (AMPcPr, 28), Apple-Pear (APr, 37), and Mei-Peach (MPc, 48). The 14 AMPcPrS-type gene families containing 453 genes are relatively conserved and ancient. The average number of genes for 14 families is 11.1 in apple, 5.9 in mei, 6.3 in peach, 6.1 in pear and 3 in strawberry. In these ancient gene families, gene duplication and gene loss events have occurred frequently in these species. Similar results could be inferred from other three types of families. There are 28 AMPcPr-type large families that lack any genes from strawberry and the mean number of genes in each family of the four species ranged from 5.3–17.8, which suggested that these families were produced in the progenitor of the four species but after the divergence from strawberry. The average number of genes per family in apple was always at least 2-fold larger than other species, and the average number in mei and peach was similar. Together with the fact that more than 1000 NBS-encoding genes were found in apple but their sister species, pear, only contained about 600 NBS genes, it is reasonable that large scale of gene duplications have occurred in the apple genome after it is raised up. Additionally, there is a family of Apple -Pear-Strawberry (APrS)-specific, containing 99 members, 49 apple genes, 45 pear genes, and 5 strawberry genes. It indicates that some ancient genes had been lost in mei and peach, and new genes emerged and spread in the progenitors of apple and pear.

To estimate and compare the evolutionary characters of genes in different types of families, the average nucleotide divergence was calculated and their selection force was estimated. Families that had fewer than 3 members were excluded from further study. On the whole, the average nucleotide divergence of genes in each species-specific gene family was much lower than in multi-species gene families (Table 4). In species-specific gene families, melon and watermelon-specific genes have lowest nucleotide divergence and strawberry and cannabis, which have large number of species-specific gene families, show higher nucleotide divergence. The average nucleotide divergence of multi-species gene families ranged from 0.109 to 0.548. Although all the average values of *Ka/Ks* <1 were observed, according to the result of PAML, 117 of 154 (76.0%) gene families were detected with significant positive selection sites and about 82.4% species-specific gene families and 72.8% multi-species gene families were significant under a positive selection (*P* < 0.05).

Gene expansions are common in NBS-encoding genes. Here, the phenomenon is also observed in Rosaceae species. It is reported that both tandem and large-scale block duplication contributed to the expansion of this gene group [38]. To check the genome organization of these expanded genes in Rosaceae species, tandem duplicated and segmental duplicated NBS-encoding genes were identified (see Methods for details). The apple genome has not been assembled into chromosomes or large scaffolds but into metacontigs and small scaffolds. Therefore, according to our definitions of tandem duplication, only 34 of 1100 NBS-endoing genes were identified as tandem duplication genes, because most genes in a gene family reside in the different scaffolds. Under the same reason, it is impossible to identify the segmental duplication events in the apple genome. Except the apple genome, we successfully identify the tandem duplication and segmental duplication in other four Rosaceae species. If the definition of physical length for tandem duplication is 100 kb, about 83.6, 73.3, 74.3 and 54.1% of NBS-encoding genes in peach, pear, plum and strawberry were respectively detected in tandem duplicated manners. These values become slight lower (75.8, 65.6, 65.9 and 49.7%, respectively) when 50 kb is used for defining the tandem duplicated genes. Conversely, in peach, pear, plum and strawberry, only 22, 36, 29 and 11 segmentally duplicated blocks with syntenically homoelogous NBS-encoding genes and their flanking genes were detected, containing 22.9, 31.7, 32.6 and 27.5% NBS-encoding genes. These results suggested that tandem duplication, but not segmental duplication, played a major role in NBS-encoding gene expansion in the four Rosaceae species.

### Phylogenetic analysis of NBS-encoding genes in Cucurbitaceae and rosacea

To analyze the evolutionary relationships of NBS genes in these relatives, three phylogenetic trees were constructed, one cucurbitaceae-specific tree, one tree containing genes from peach, mei and strawberry and an apple-pear tree (Figures 2, Additional file 1: Figure S1, Additional file 2: Figure S2, and Additional file 3: Figure S3). TIR NBS genes and non-TIR NBS genes were clearly separated in all of the three trees. To survey how many NBS genes were produced after each species splitting, the species-specific clades were defined if the gene number of species-specific genes are larger than 2, the minimum nucleotide similarity >80% and the bootstrap value >50%. These defined clades indicated the genes experienced recent expansion in each species.

No cucumber-specific clade was detected in cucurbitaceae-specific tree (Figure 2 and Additional file 1: Figure S1). Except for several clades that contained exactly two cucumber genes each, cucumber genes always clustered with melon genes. Two watermelon-specific clades, each containing three genes, were found and all the three copies were very similar (nucleotide similarity >95%), suggesting that genes in the two clades expanded recently. One large melon-specific clade was found to have eight members, which was the largest species-specific clade in the cucurbitaceae tree. Only 0–13.0% of all the NBS genes in cucurbitaceae were supposed to expand recently, which states that there have been very few duplications of NBS genes after speciation of cucumber, melon and watermelon. Another 13 clades of single-copy alleles retained gene order in three-way genome comparisons were defined. All the cucumber genes in these clades were found to be closer to melons genes. This was consistent with the genetic relationship among these three species. Outside of these 13 clades, others were present in only one or two genomes, showing presence and absence polymorphism among different species. This low number of species-specific clades in cucurbitaceae tree demonstrated that the three species split not long ago.

At the bottom of the cucurbitaceae tree (Figure 2), more than 10 melon NBS genes, including TIR and non-TIR genes, with long branch were found. This and the phenomena that no genes were very similar between cucumber and watermelon, these results indicated that the melon genes were relative ancient and retained from ancestors but lost in cucumber and watermelon. For the phylogenetic tree of peach, mei and strawberry, 8 peach-specific, 15 mei-specific and 26 strawberry-specific clades were defined (Additional file 2: Figure S2). The average member of genes in the three types of clades was 3.4, 3.1, and 4.8. The two largest clades both contained strawberry-specific genes. One had 12 members and the other had 13 genes. Strawberry had more new produced NBS genes. Although strawberry had the lowest number of NBS genes of any of the least in the three genomes, there were many other large strawberry-specific gene clades that showed considerable higher nucleotide divergence. The species-specific gene clades found in apple-pear tree were much more numerous (Additional file 3: Figure S3). Here, 84 apple-specific clades and 30 pear-specific clades were identified, including 330 (25.3%) and 113 (18.3%) genes, respectively. There were more apple-specific clades but they had lower bootstrap values due to the large number of sequences. Apple contained more than twice as many NBS genes as pear. This very strongly indicated that after the split of apple and pear, apple experienced a large gene duplication event.

## Discussion

### Small numbers of NBS-encoding genes in Cucurbitaceae

Compared with other reported reports, the numbers of cucurbitaceous NBS-encoding genes are relatively small [21]. In the current study, the three cucurbitaceous genomes, cucumber, melon and watermelon only harbor 45-80 NBS-encoding genes (0.19–0.27% of total genes). All the sequenced plant genomes except *Carica papaya* were found to contain more than 100 NBS-encoding genes [11]. The average percentage of NBS genes among all the genes in surveyed plant genomes ranged from 0.6% to 1.8% [16]. Compared the genome size and the whole genome gene number of the cucurbitaceous species with the other plants, the cucurbitaceous species did not stand out in either genome size or number of genes in the genome. Their lack of NBS-encoding genes is most possibly due to the loss of NBS-encoding genes after their split from other species.

To Cucurbitaceae, grape is an outer group species and Rosaceae is a parallel group, whereas all these plants have many more NBS-encoding genes (>300) than Cucurbitaceae. The Cucurbitaceae species are annual herbaceous plants, having short generation time. Herbaceous species are often regarded as faster evolving than woody species. Compared with wood perennial plants, short life history might benefit these annual plants to catch the evolutionary rates of pathogens [37,39,40]. Unlike the perennial species, for instance, the Rosaceae species, few recent gene duplications of *R* genes are found in Cucurbitaceae. This indicates that few duplication events of NBS genes have happened after speciation of cucumber, melon and watermelon. Whole-genome sequences of cucumber, melon and watermelon revealed that the three genomes are absence of recent whole-genome duplications [29-31]. These duplications are very common in angiosperms and this process provides raw materials for gene genesis. However, the evolutionarily important recent and recurrent whole-genome duplication is absent in the three Cucurbitaceae species. Due to a mass of loss and little duplication of NBS genes, the Cucurbitaceae have very low copy numbers of NBS-encoding genes. It is reported that there is fitness cost of resistance gene [41]. High copy numbers of resistance genes might be not benefit for plants in absence of corresponding pathogens. As a cost, the plants might grow slowly, have low seed productions or taste not good enough. Cucumber, melon and watermelon are all economically important crops and products of human selection. In order to cater to human needs, these cucurbitaceous plants are reserved as what they now look like.

Actually, cucumber, melon and watermelon suffer from a range of fungal and viral diseases, such as downy mildew, angular leaf spot, bacterial wilt, and anthracnose. The Cucurbitaceae may have other specific defense mechanisms beyond NBS-encoding genes. One possible mechanism is the lipoxygenase (LOX) genes. The LOX gene family creates the oxidized fatty acid catalyzer and is considered involved in plant defense and pest resistance [42]. Usually, plant LOXs provide front-line defense against pathogens in plant immunity. Recent studies have shown that the LOX gene family in rice plays an important role in blast pathogen infection [43]. It is reported that the LOX gene family has been notably expanded in the cucumber and watermelon genomes [30,31]. This indicates that the expanded LOX gene family may be a complementary or candidate mechanism by which plants to deal with pathogens. However, expansion of the LOX gene family in the melon genome has not been found [29]. The number of NBS genes in melon is larger than in cucumber and watermelon. It is not necessary for melon to produce large number of LOX genes as cucumber and watermelon.

The LOX gene family cannot completely replace NBS-encoding genes with respect to disease resistance. Rice, grapevine, poplar and many other plants also have some LOX genes [30], but they still have more than 400 NBS-encoding genes. The reason for the deficient NBS-encoding genes in cucurbitaceous plants needs further study.

### Expansion of NBS-encoding genes in the apple genome

Although Rosaceae has a worldwide range and is thriving, it is subject to many various pathogens, such as the bacterial disease fire blight, and the fungal diseases, rust and powdery mildew. Genome-wide analysis of *R* genes in Rosaceae revealed that the rose family contained a relatively large number of *R* genes. Meanwhile, the number of genes and the proportions of *R* genes in the five surveyed Rosaceae species were not totally identical.

According to the species relationships of the five species, peach and mei are similar to each other, pear and apple are more closer and strawberry is relatively more distant to them. Peach and mei have numbers of genes and similar proportions of NBS genes. There are 48 peach-mei lineage gene families, containing 235 genes. It is obvious that these two species have the identical evolutionary patterns in *R* genes after they split from common ancestor. Among the five species, strawberry is the most different from the others. This is because it is a woodland and herbaceous species with a short generation time while the others are tree species. Strawberry has the fewest *R* genes of any Rosaceae species, which might owe to the specific characters of strawberry plants, especially the short generation time. Strawberry might rely on their rapid breeding and reproduction to escape from the invading of pathogens. The strawberry genome is the only plant genome sequenced to date with that shows no evidence of whole genome duplication, which is found in all other rosid genomes. This might be the direct reason for the small number of the strawberry genome.

A recent whole genome duplication (WGD) event was shared by apple and pear, but peach, mei and strawberry has not undergone recent WGD (Figure 1). It is therefore not strange that the genomes and number of genes of apple and pear are much larger than those of the three relatives. The *R* gene numbers found in pear and apple are also larger than the other three species. However, the number of *R* genes found in pear is much lower (2 fold lower) than that of the closely related apple genome. Even though the sequenced genome of apple is larger than that of other plants within the Rosaceae, the relative number of NBS-encoding gene is still highest (2%), which is the largest proportion reported so far in any plant, except bread wheat [44].

Based on the phylogenetic tree of pear and apple, large number of apple-specific clades was found. And the results of classification of gene families show high number of apple-specific gene families while for apple-pear lineage gene families, the average numbers of gene in apple was 2.3 times higher than that of pear. It can reasonably be inferred that many NBS-encoding genes in the apple genome might be produced after apple-pear differentiation. It has been reported that the WGD events and tandem duplications are responsible for the high number of NBS genes in the apple genome [8,10]. These and the present results suggest that recent WGD might contribute to the expansion of R genes in the common ancestor of apple and pear, resulting in more R genes in Maloideae. After the split between apple and pear, more small-scale duplications have taken place in the apple genomes, leading to a great increase in the number of *R* genes. It is not clear why so many more *R* genes were retained in apple because pear and apple are close cousins species and they might have diverged from each other 5.4–21.5 million years ago (MYA). Pear has a cultivation history of 3000 years and domesticated apple appeared around 4000 years ago. Their habitats are also similar. The retention of so many R genes in apple might be the result of selection during domestication. Apple might encounter more diseases than pear, such as some rust. These *R* genes might be kept as a library to cope with uncertain and unknown pathogens. The real reason for the huge number of *R* genes in apple requires more materials and evidence.

## Conclusion

This study provides a genomic framework for the identification of NBS-encoding genes in Rosaceae and Cucurbitaceae through comparative genomics. Considerable differences in the copy number of NBS-encoding genes were observed between Cucurbitaceae and Rosaceae species. In Rosaceae species, a large number and a high proportion of NBS-encoding genes were observed in peach (437, 1.52%), mei (475, 1.51%), strawberry (346, 1.05%) and pear (617, 1.44%), and apple (1303, 2.05%). The number of apple NBS genes might be the largest number in all of the reported diploid plants. However, only 45-80 NBS-encoding genes (0.19–0.27%) were identified in Cucurbitaceae. Comprehensive analysis of NBS-encoding genes, including phylogenetic analyses, calculation of nucleotide divergence and estimation of selection forces, indicates that NBS-encoding genes in Rosaceae crops, especially in apple, have undergone extreme expansion and rapid adaptive evolution. This research could contribute to a better understanding of the evolutionary history of NBS-encoding genes in Rosaceae.

## Methods

### Sequence retrieval and identification of NBS-encoding genes

Nine whole-genome sequenced plants were used in the present study, including three Cucurbitaceae, cucumber (*Cucumis sativus*, http://www.icugi.org/cgi-bin/ICuGI/index.cgi, http://csgenome.sggw.pl/, http://phytozome.jgi.doe.gov/pz/portal.html#!info?alias=Org_Csativus), melon (*Cucumis melo*, https://melonomics.net/files/Genome/) and watermelon (*Citrullus lanatus*, http://www.icugi.org/cgi-bin/ICuGI/index.cgi); five Rosaceae, peach (*Prunus persica*, http://www.rosaceae.org/peach/genome), mei (*Prunus mume*, http://prunusmumegenome.bjfu.edu.cn/), strawberry (*Fragaria vesca*, ftp://ftp.jgi-psf.org/pub/compgen/phytozome/v9.0/Fvesca/), pear (*Pyrus bretschneideri Rehd*, http://peargenome.njau.edu.cn), and apple (*Malus domestica*, ftp://ftp.jgi-psf.org/pub/compgen/phytozome/v9.0/Mdomestica/. Cannabis (*Cannabis sativa*, http://www.ncbi.nlm.nih.gov/nuccore/JP449145) served as an outgroup.

A three-step process was used to identify the greatest possible number of candidate NBS-encoding genes in the surveyed species. First, the predicted protein sequences in the given annotation data were used. All the candidate genes that presented NB-ARC domains (Pfam: PF00931) from Pfam results (E value cut-off of 10^-4^) were selected and considered as candidate NBS-encoding genes. Second, to find NBS genes that might be ignored in the intergenic regions, the amino acid sequence of the NB-ARC domain was used as a query to BLASTp against the genome sequences. All BLAST hits, together with flanking regions of 5000 base pairs on both sides, were annotated using the gene-finding programs FGENESH with the training set of the closest species (http://www.softberry.com/). To exclude potentially redundant candidate NBS-encoding genes, all candidate NBS genes were orientated by BLASTn, and sequences located in the same location were eliminated. Last, all non-redundant NBS-encoding genes were surveyed to further confirm whether they encoded NBS or LRR motifs using the Pfam database v23.0 (http://pfam.janelia.org/), SMART protein motif analyses (http://smart.embl-heidel-berg.de/). CC motifs were detected using COILS program with a threshold of 0.9 in the first 200 amino acids (http://www.ch.embnet.org/software/COILS_form.html).

### Alignment and analysis of gene families

To facilitate calculation of genetic parameters and identify the different characteristics of various genes, all NBS-encoding genes were classified into families based on the sequence similarity >60% and coverage >60%. Multiple alignments of amino acid sequences were performed using ClustalW with default options. The resulting alignments were then used to guide the alignment of nucleotide coding sequences using MEGA 6.0 [45].

For each gene family, the average nucleotide diversity or divergence (π or Dxy) was estimated with the Jukes and Cantor correction using DnaSP v5.0. The number of nonsynonymous substitutions per nonsynonymous site is here denoted by Ka while the number of synonymous substitution per synonymous site is denoted by Ks. The ratio of nonsynonymous to synonymous nucleotide substitutions (*Ka/Ks*) among paralogs were evaluated using MEGA 6.0 based on the Nei-Gojobori method with Jukes–Cantor correction. Diversifying selection or positive selection was investigated using PAML [46,47]. Models M7 and M8 in program ‘codeml’ of PAML were run for all gene families with more than two members. Positive selection was confirmed using a likelihood-ratio test by comparing the likelihood calculated using models M8 and M7.

### Determining tandem duplication genes and segmental duplication genes

Tandem duplicated NBS-encoding genes are defined as those closely related genes in the same family falling with 50 kb or 100 kb of one another. To investigate the segmental duplication events containing NBS-encoding genes, all NBS-encoding genes in a gene family were oriented on the chromosomes or scaffolds by using BLASTn. Thirty genes on the same chromosomes or scaffolds, including the NBS-encoding gene and 15 flanking genes on each side, were then compared by pairwise BLAST analysis to identify duplicated genes between two independent segmental blocks. If more than five gene pairs with syntenic relationships (BLAST E-value < 10^-10^) were detected, the two blocks were defined as segmentally duplicated regions.

### Phylogenetic analysis of NBS-encoding genes

Generally, for NBS-encoding genes, the regions that follow the NBS, such as LRR regions, have high variability and not included for phylogenetic construction. For this reason, only the NBS regions were used to build phylogenetic tree. All proteins of NBS-encoding genes were trimmed to extract the NBS domain sequences according to Pfam results. Then, multiple alignments of these amino acid sequences were performed using ClustalW with a default option. The aligned amino acid sequences were transferred to nucleotide sequences again and used to construct a phylogenetic tree using MEGA 6.0, based on neighbor-joining (NJ) method. A Kimura two-parameter model and the internal node stability were explored with 1000 replicates.

### Availability of supporting data

The phylogenetic data has been deposited in TreeBase (http://purl.org/phylo/treebase/phylows/study/TB2:S17504).

## Additional files

Additional files 1: Figure S1. Phylogenetic tree based on NBS domain of NBS-encoding genes in cucumber, melon and watermelon. Additional files 2: Figure S2. Phylogenetic tree based on NBS domain of NBS-encoding genes in peach, mei and strawberry. Red lines represent TIR genes and black lines represent non-TIR genes. NBS-encoding genes in peach, mei and strawberry are shown as pink circles, purple circles, and dark green circles, respectively. The vertical bars with different colors beside the tree are used to represent species-specific gene clades of three different species. Additional files 3: Figure S3. Phylogenetic tree based on NBS domain of NBS-encoding genes in apple and pear. Red lines represent TIR genes and black lines represent non-TIR genes. Apple NBS genes are shown as red circles and pear NBS genes are shown as green circles. The red brackets and green brackets respectively indicate the apple-specific gene clades and pear-specific gene clades. Additional files 4: Table S1. Exon statistics in TIR and non-TIR NBS-encoding genes.
