# Supplementary figures and images for: Extreme expansion of NBS-encoding genes in Rosaceae
Source: BMC Genet. 2015 May 3;16:48. doi: 10.1186/s12863-015-0208-x (PMC4417205; doi:10.1186/s12863-015-0208-x)

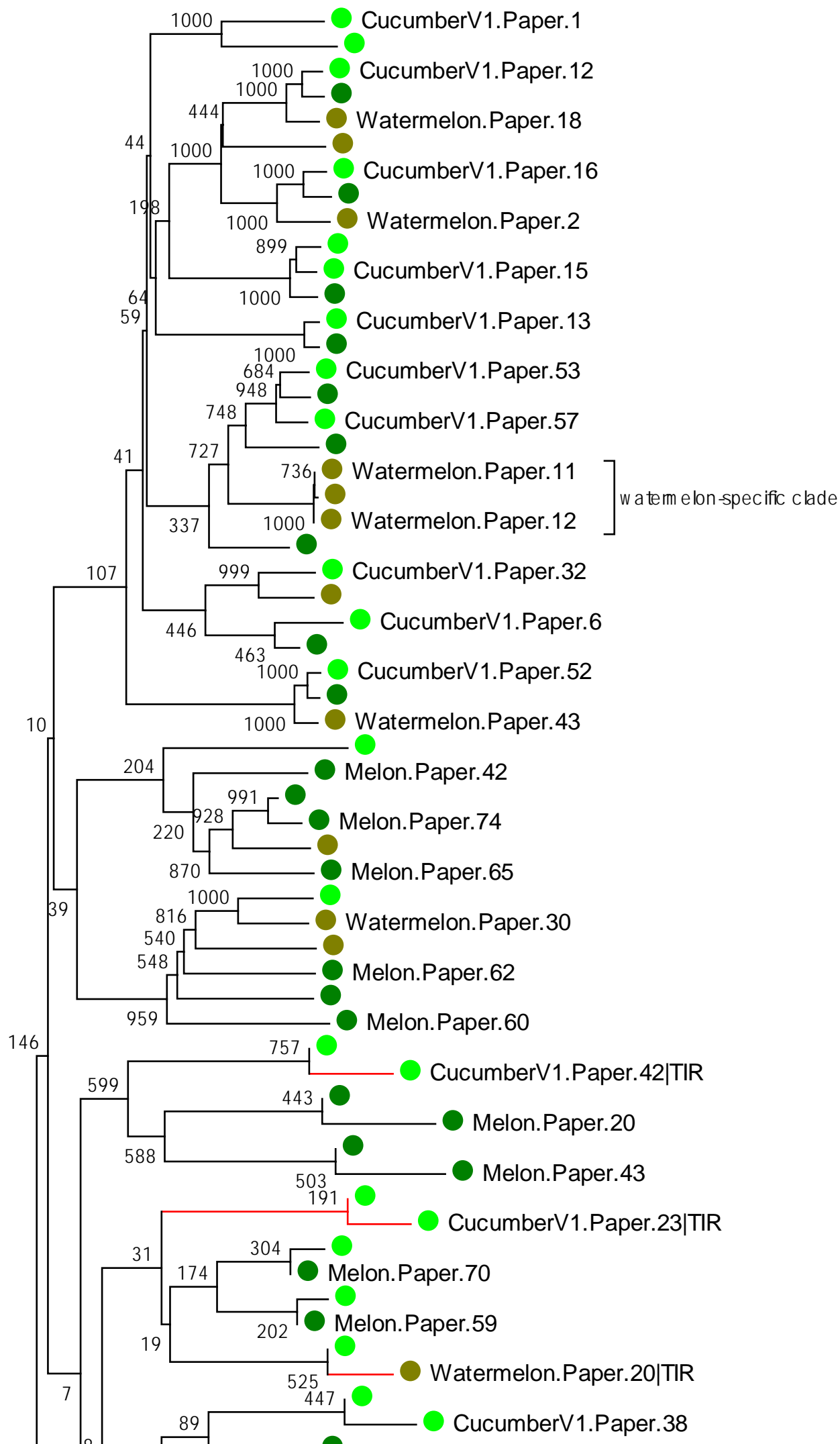

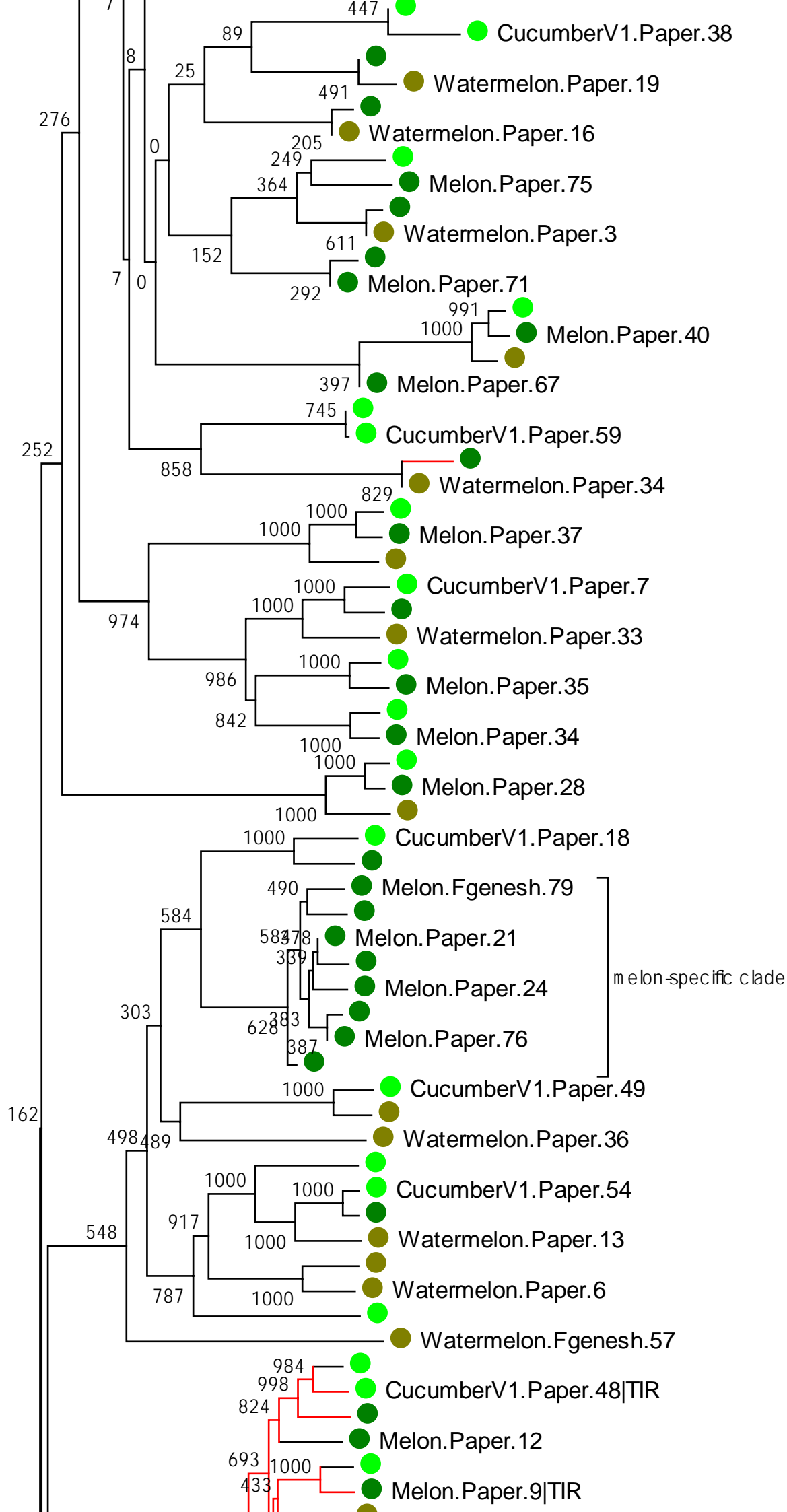

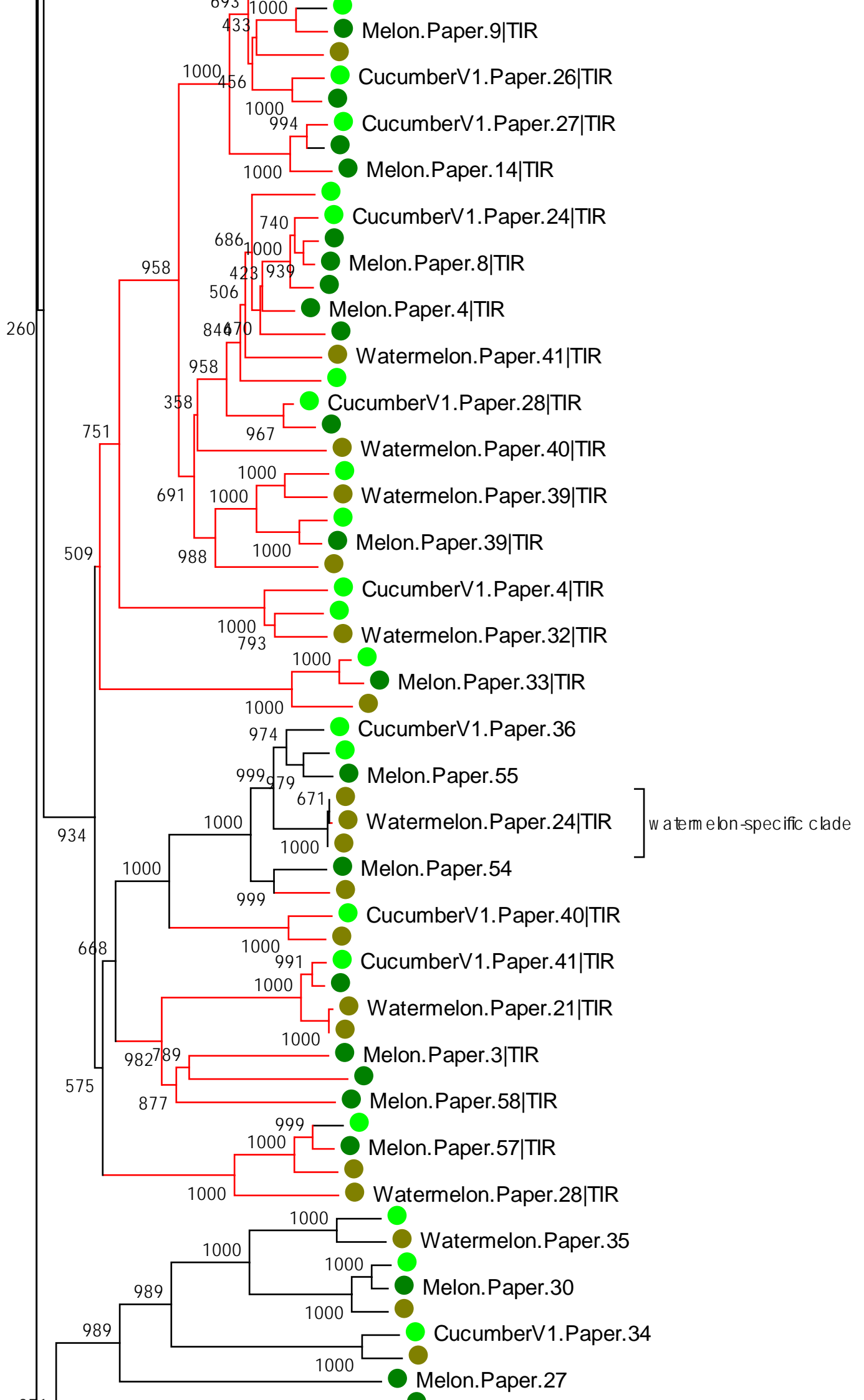

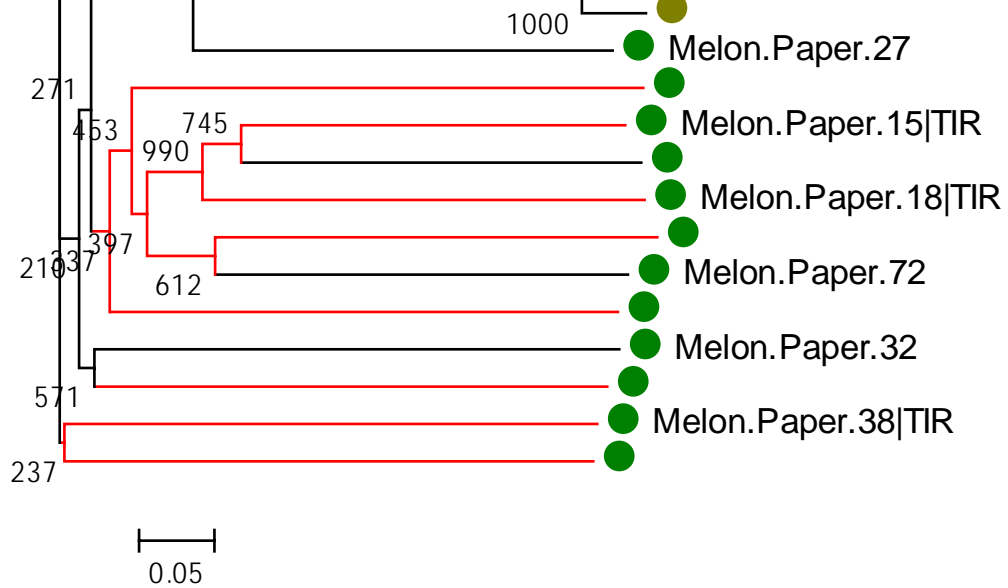

Supplement: Additional files 1: Figure S1. — Phylogenetic tree based on NBS domain of NBS-encoding genes in cucumber, melon and watermelon. [file 12863_2015_208_MOESM1_ESM.pdf]

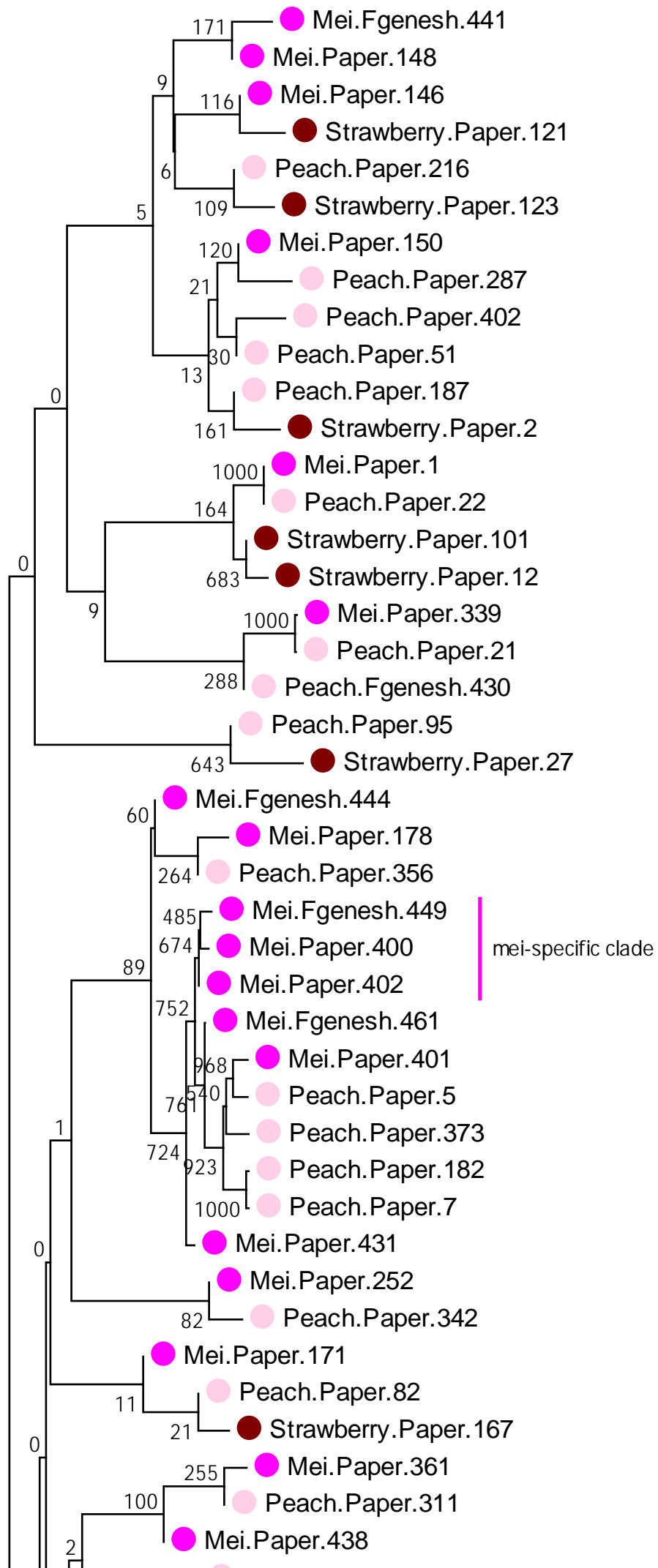

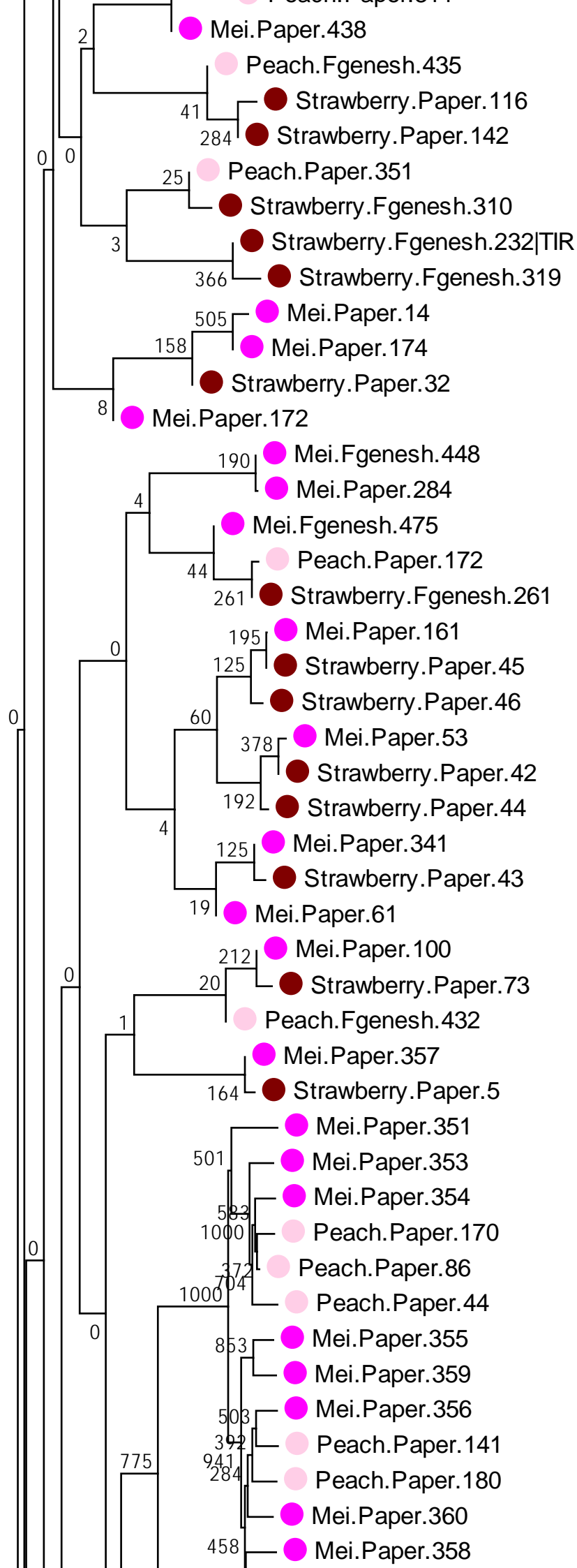

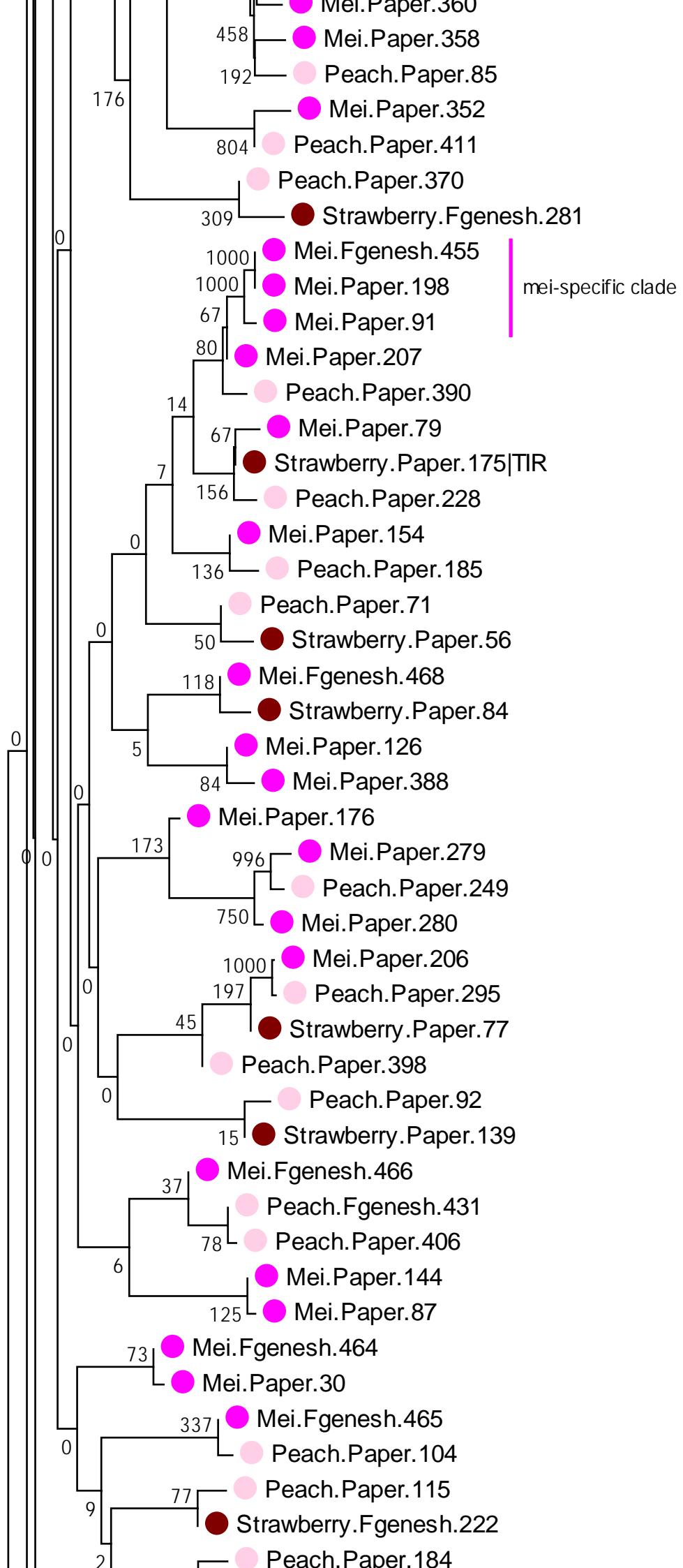

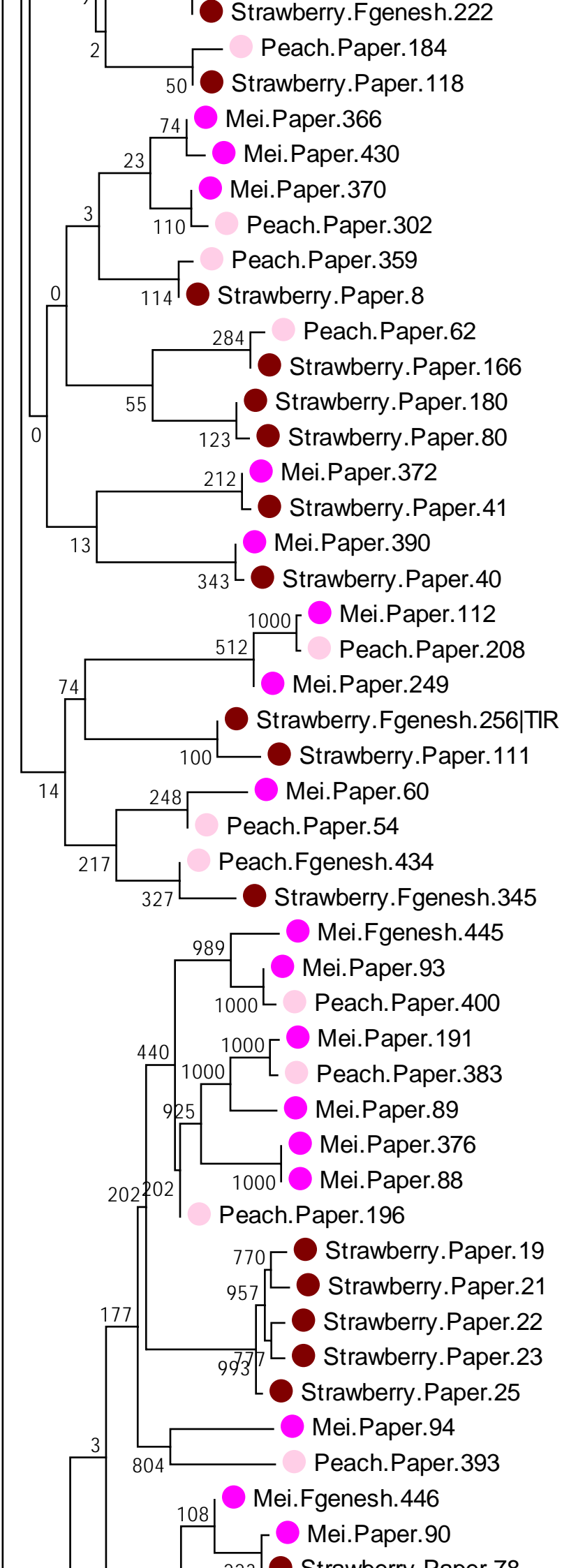

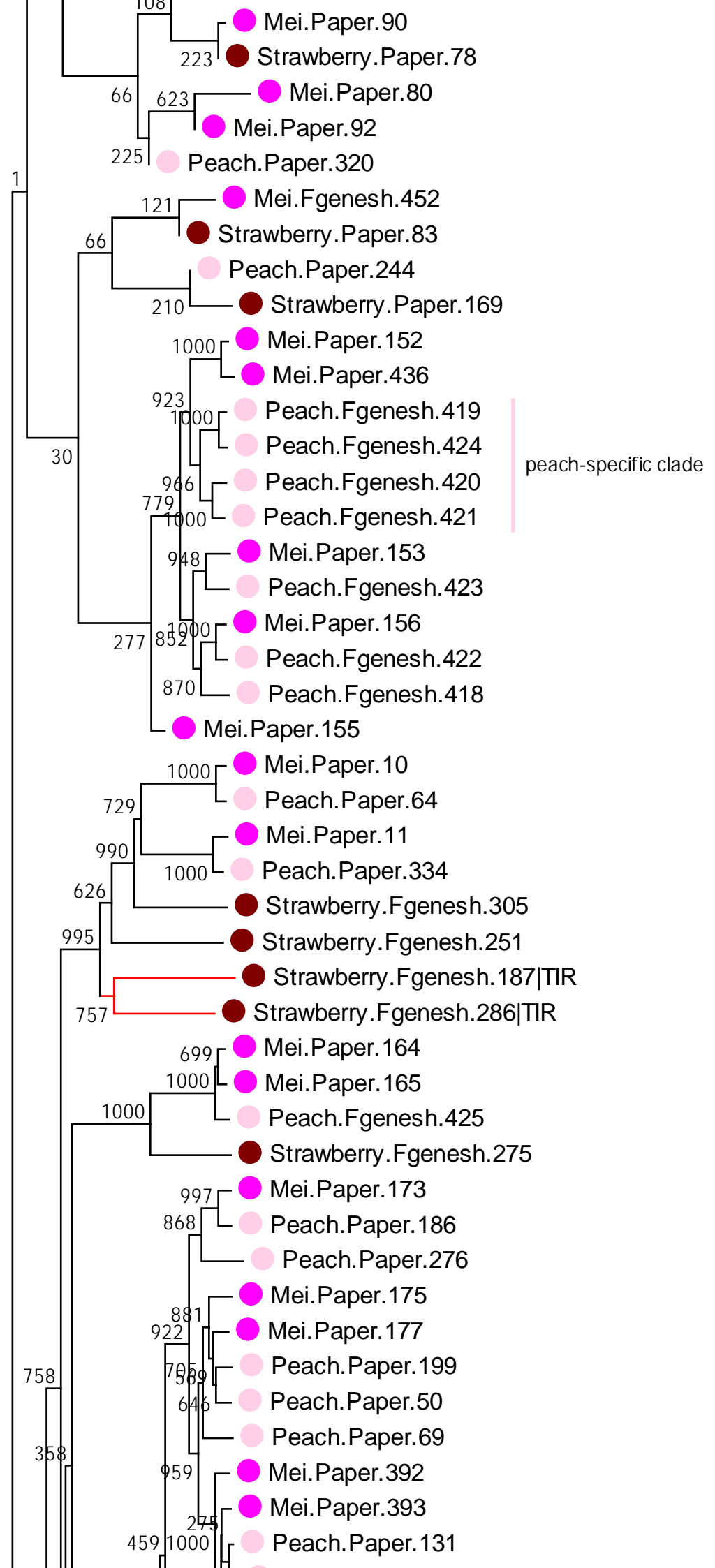

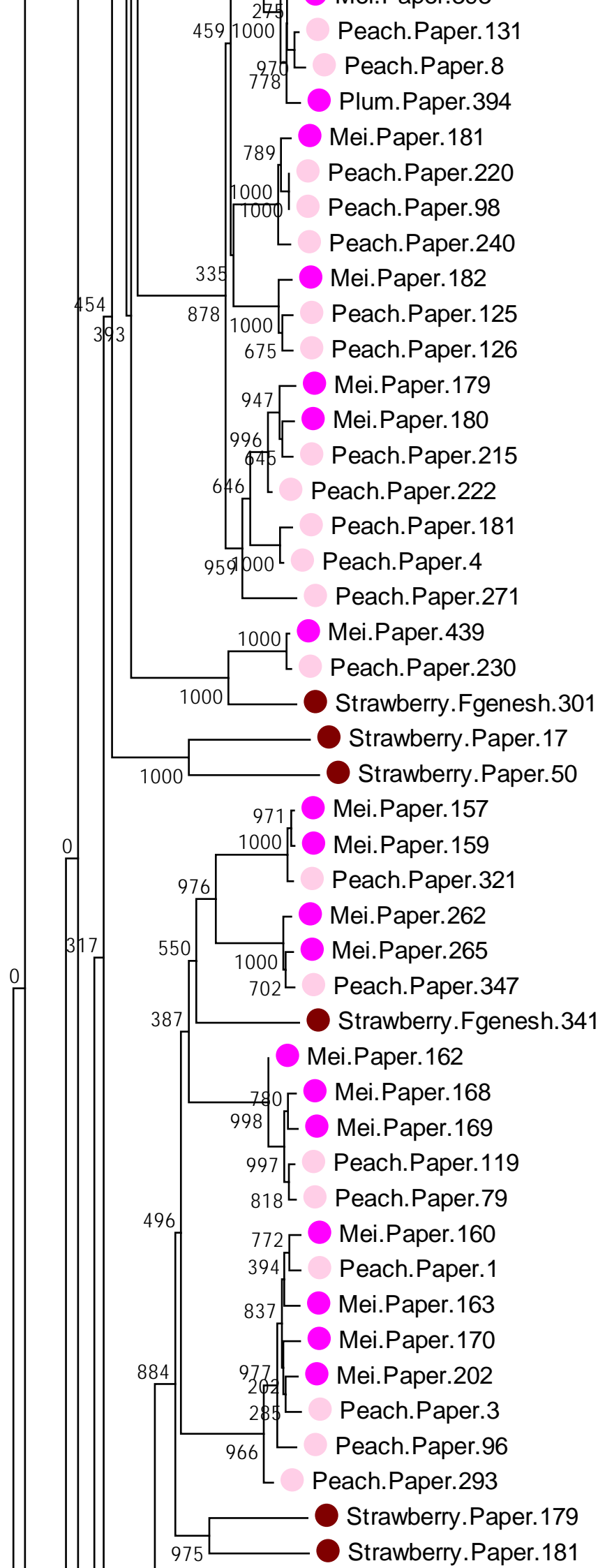

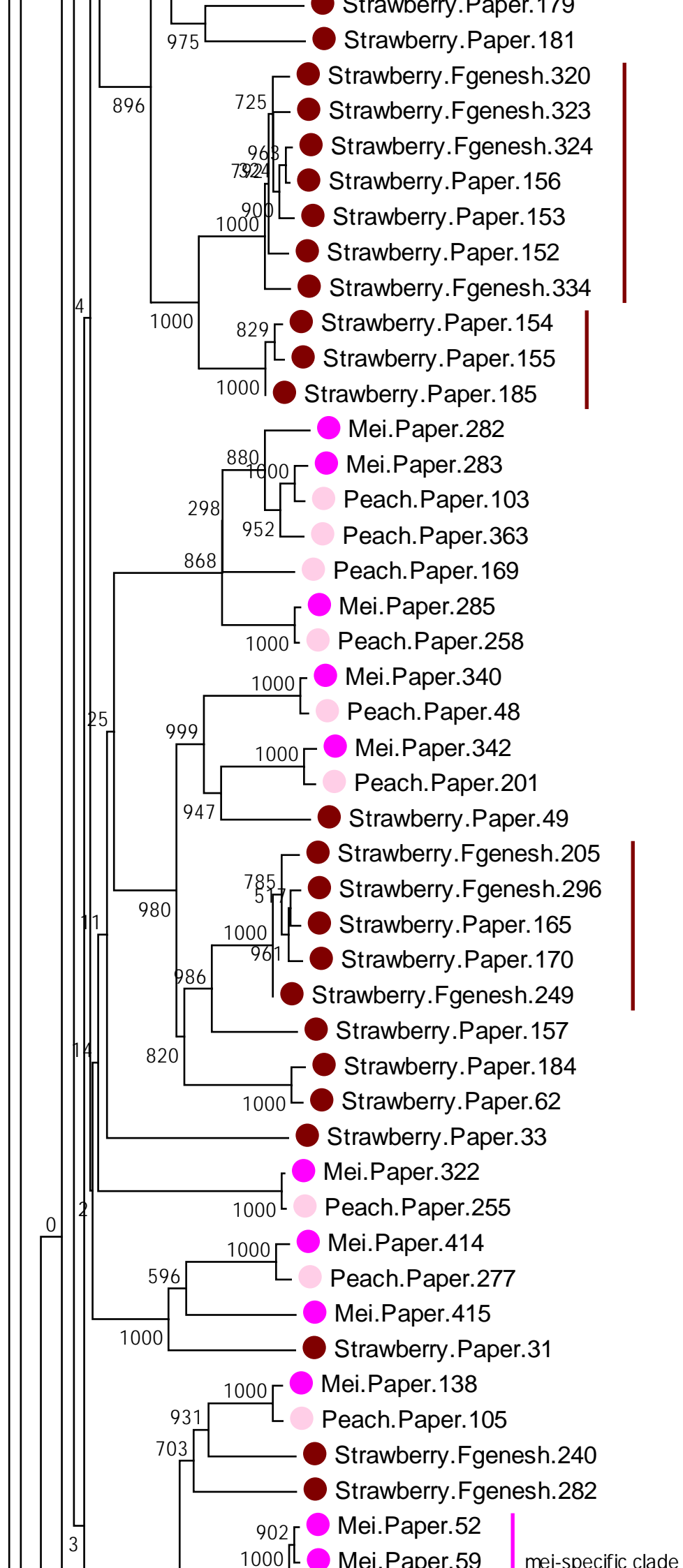

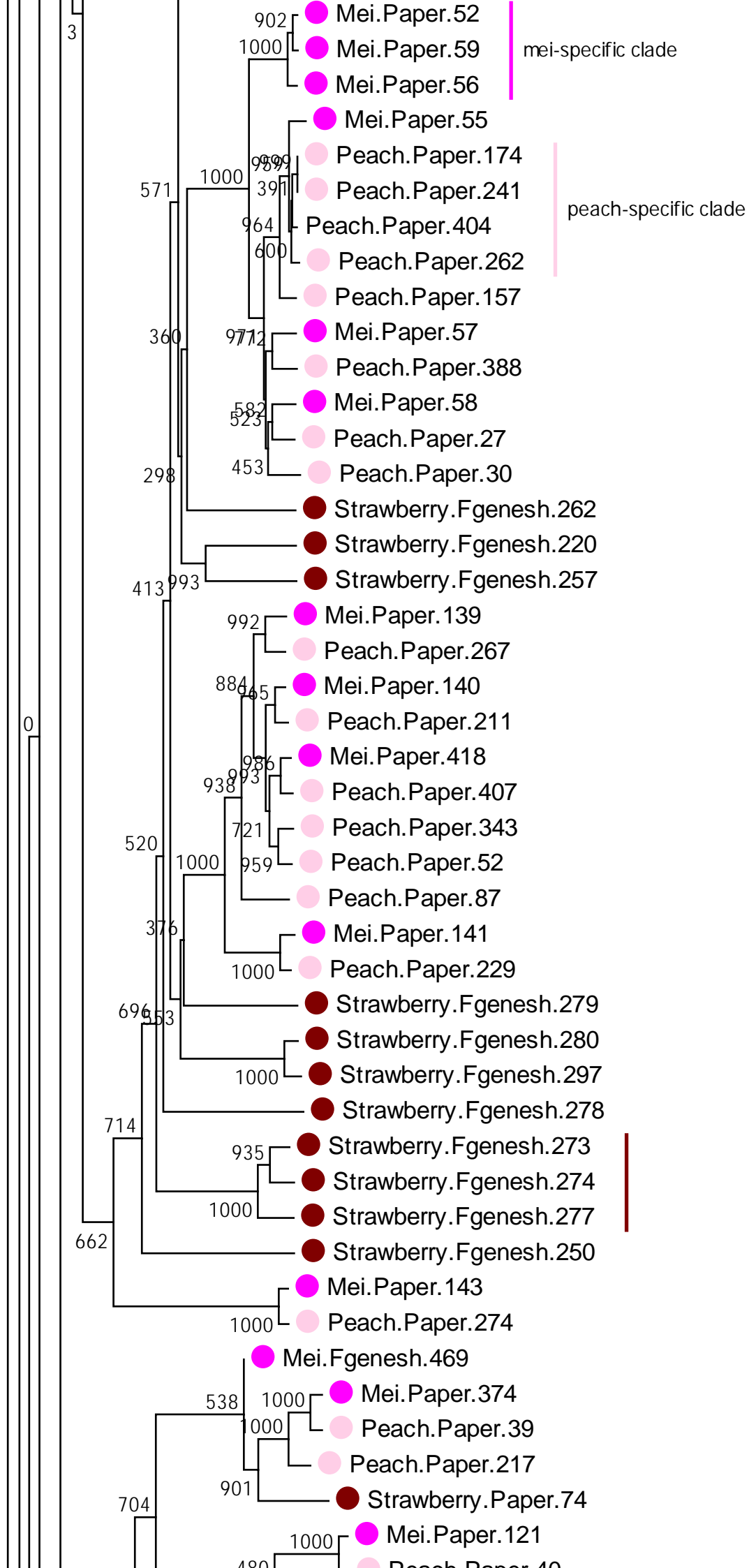

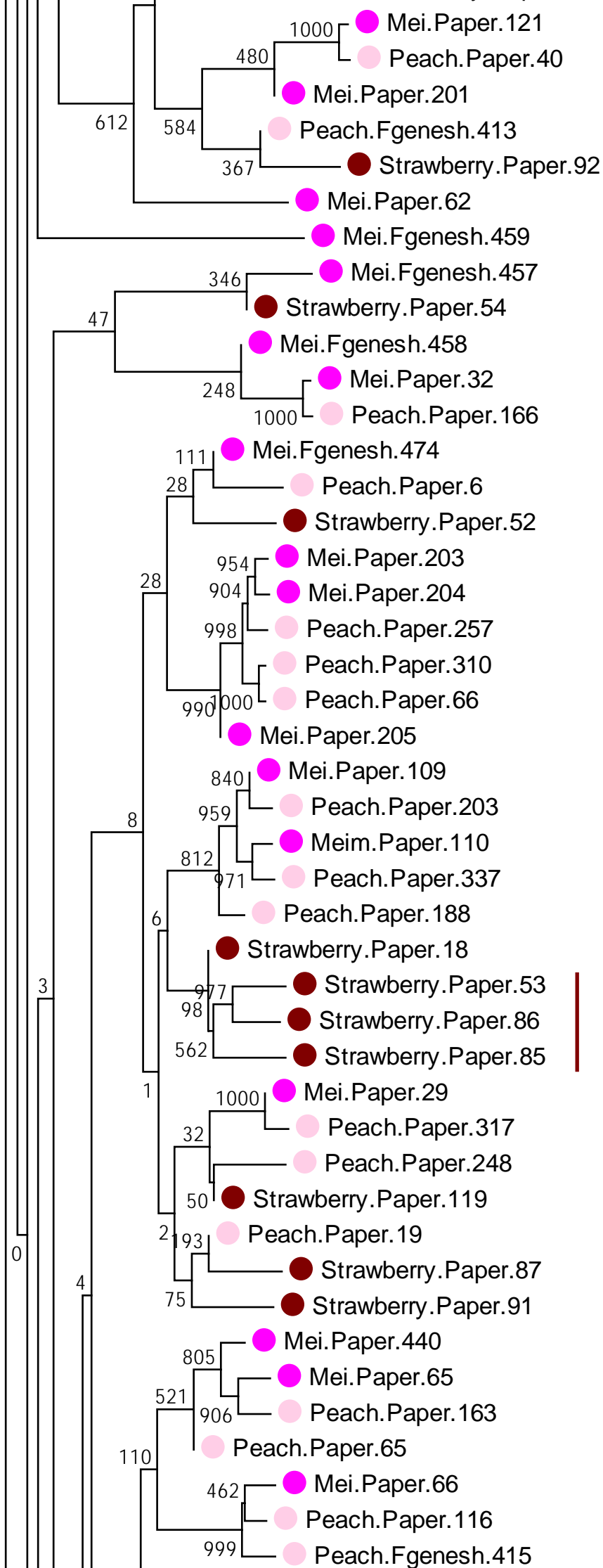

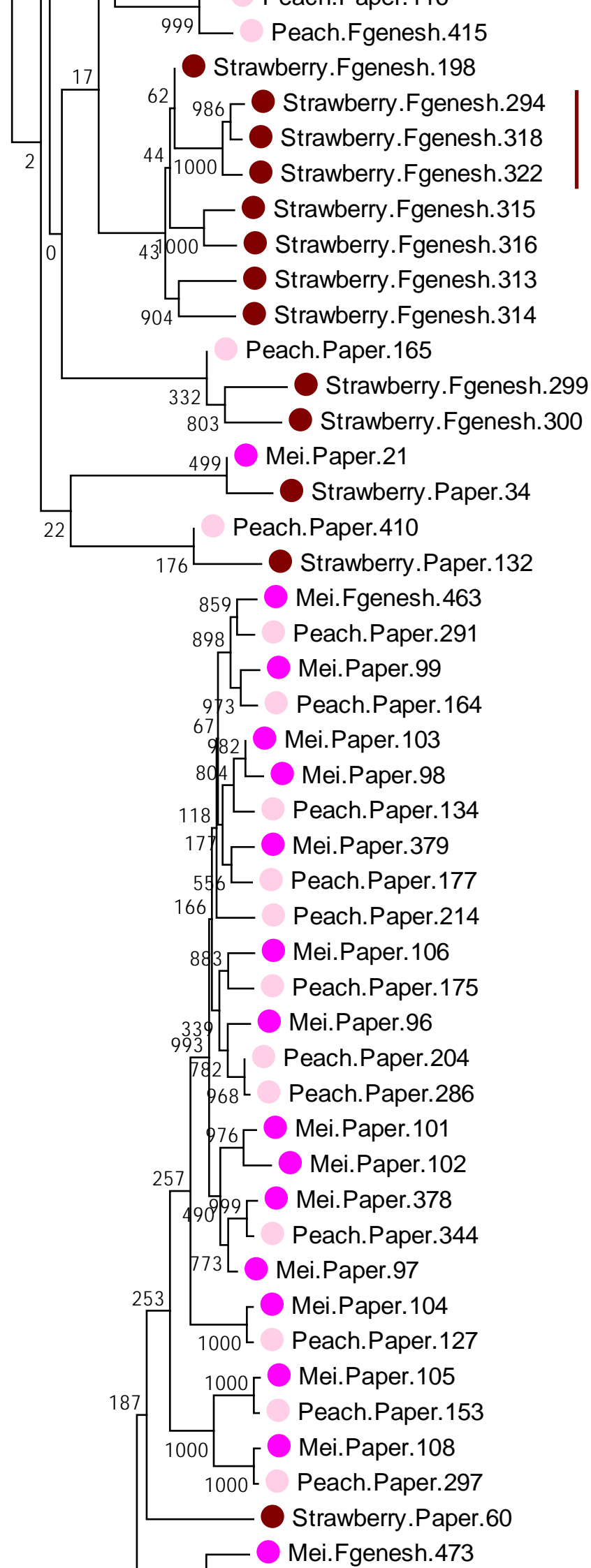

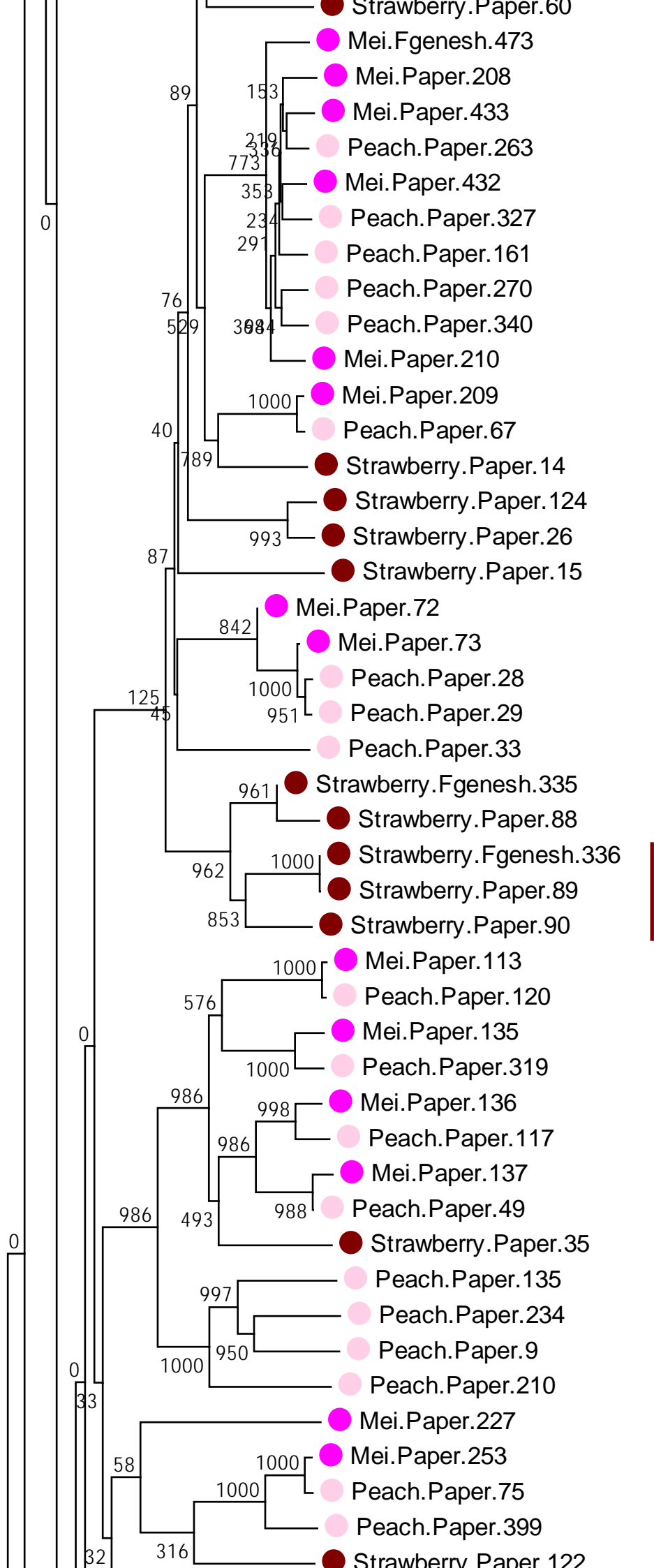

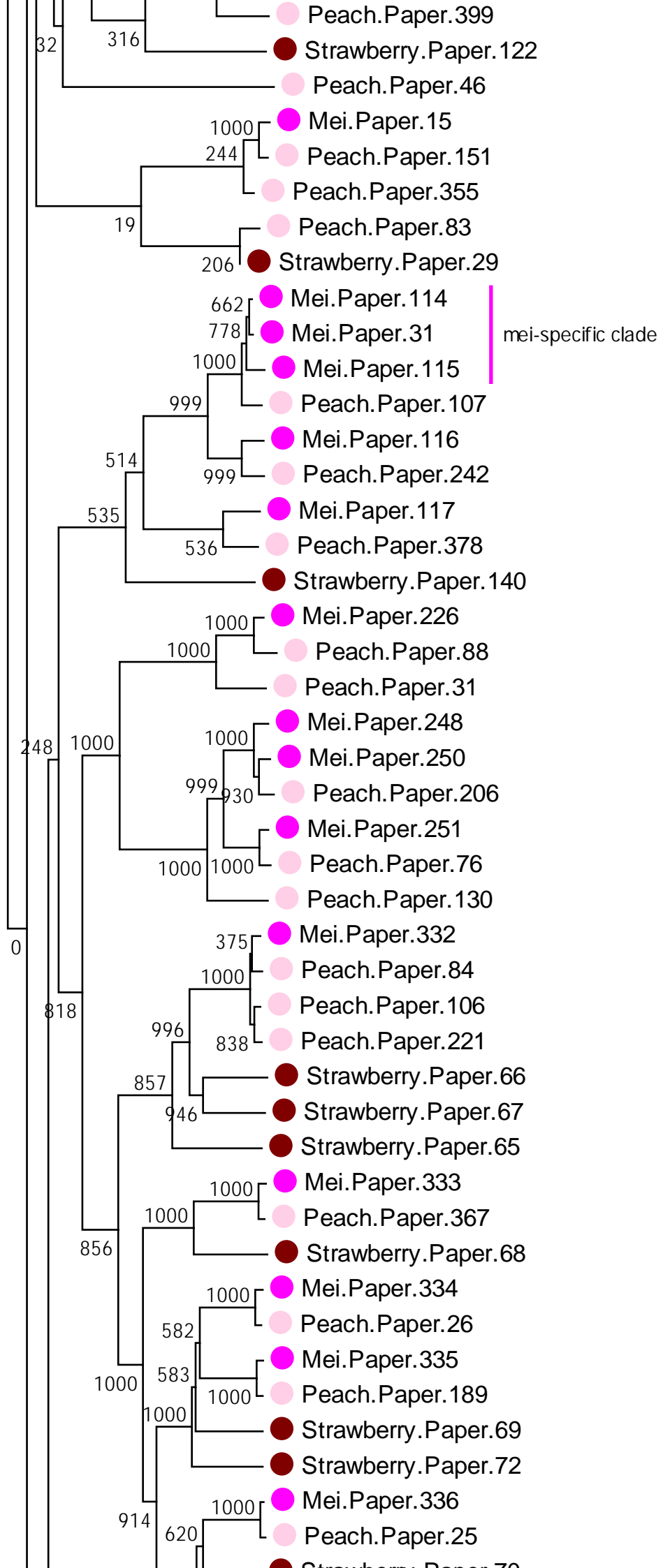

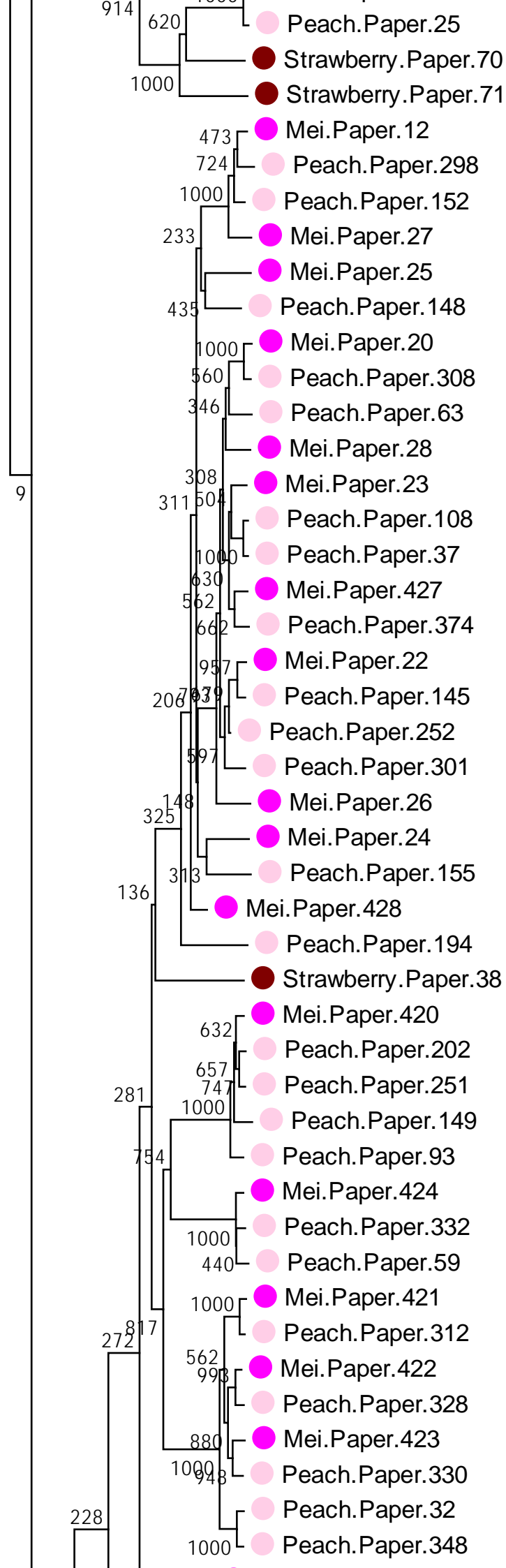

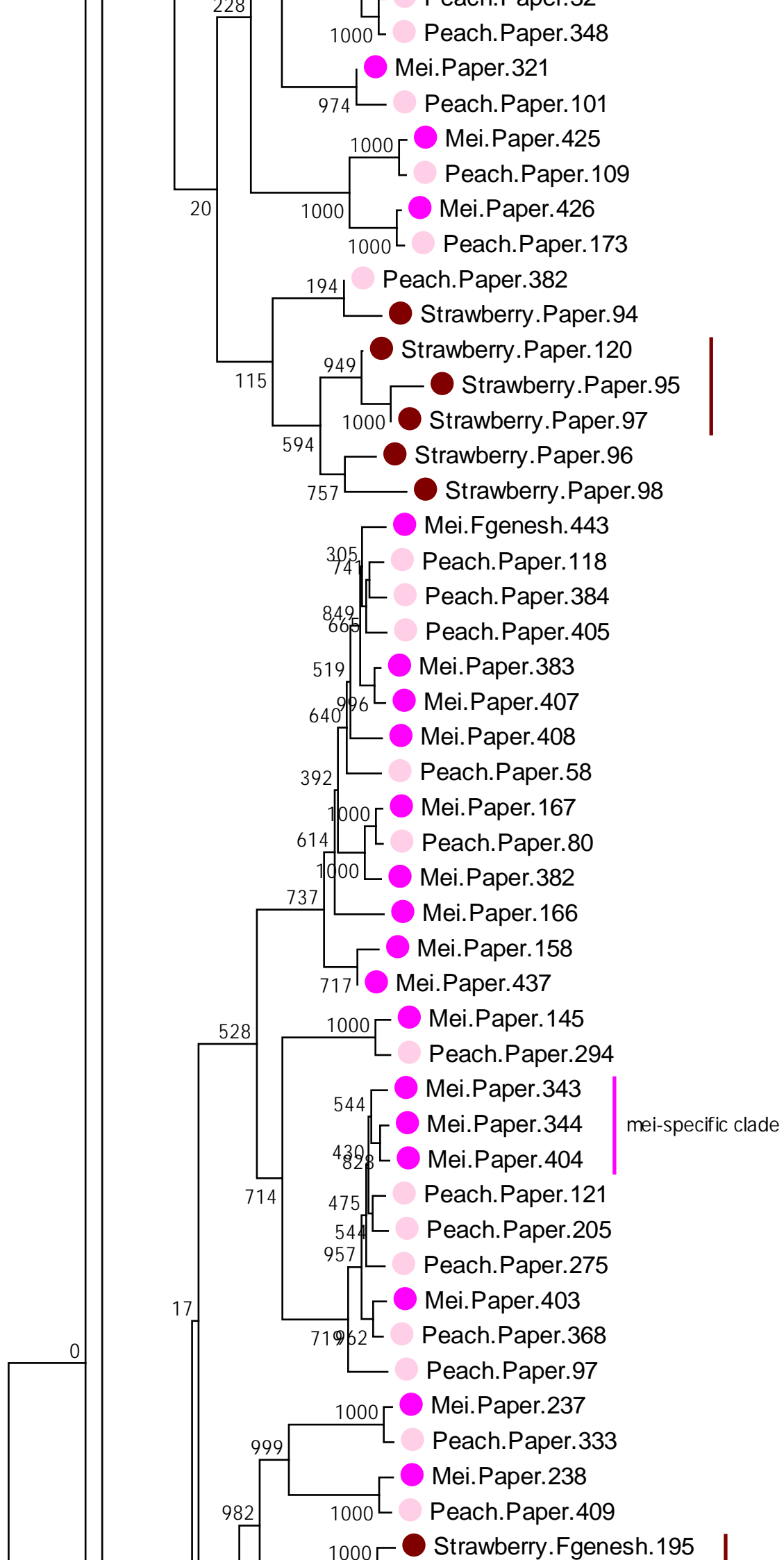

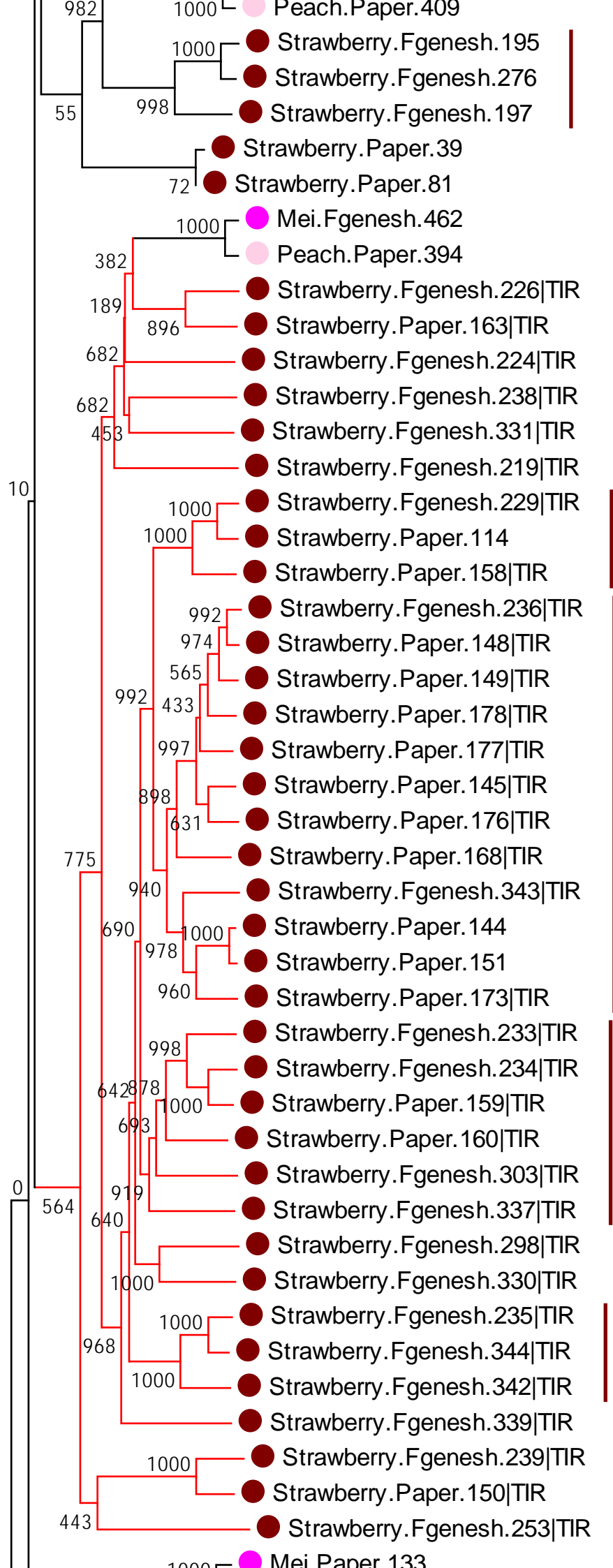

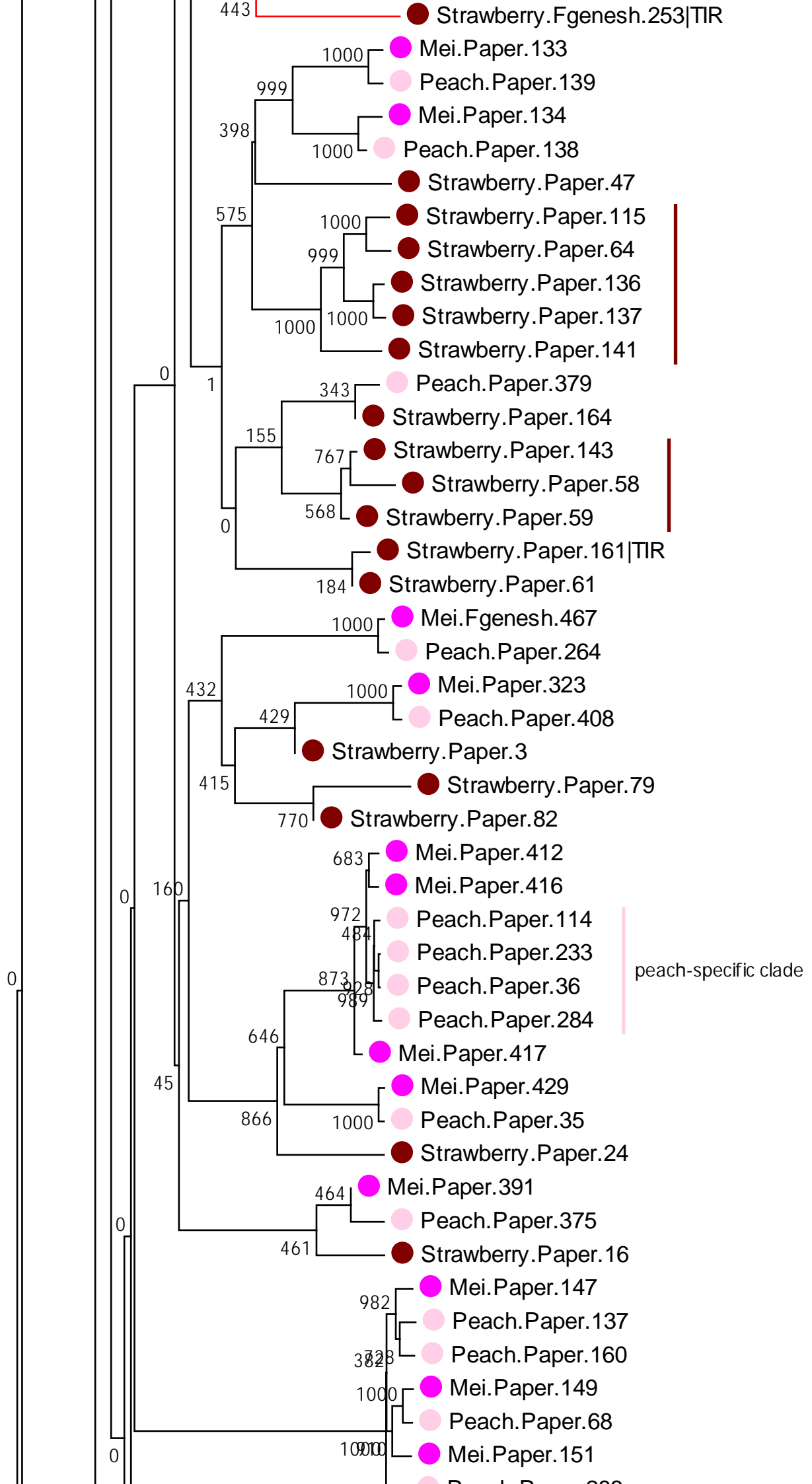

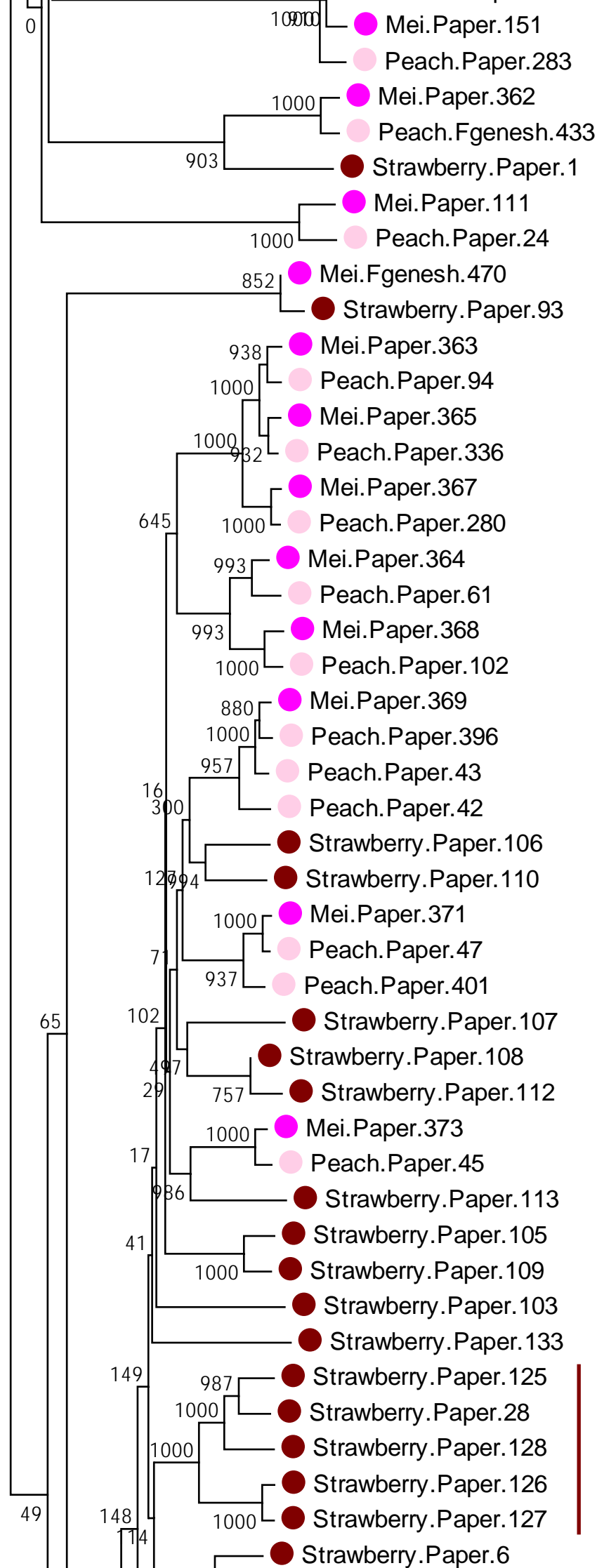

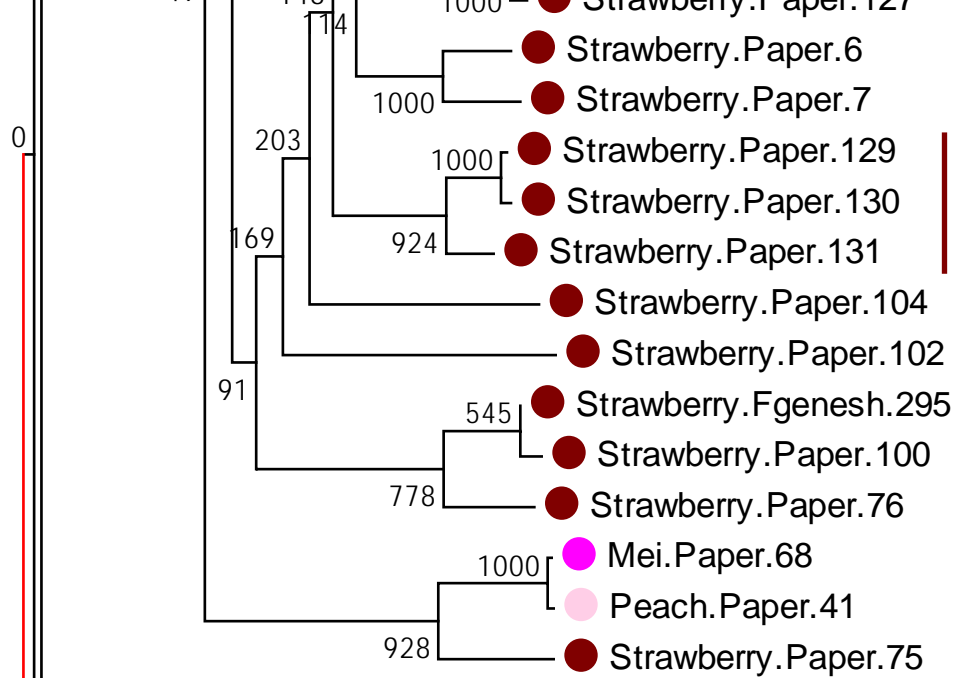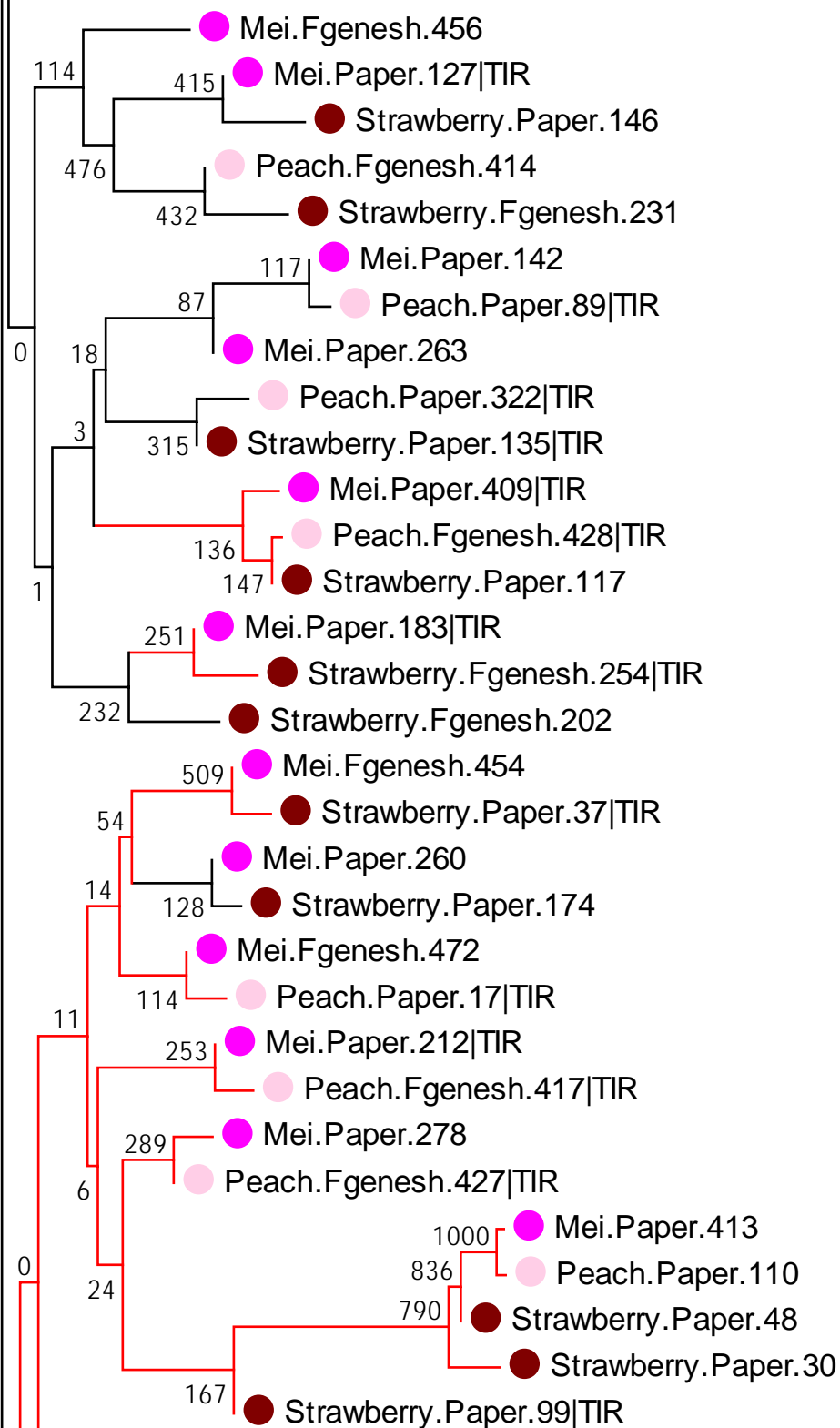

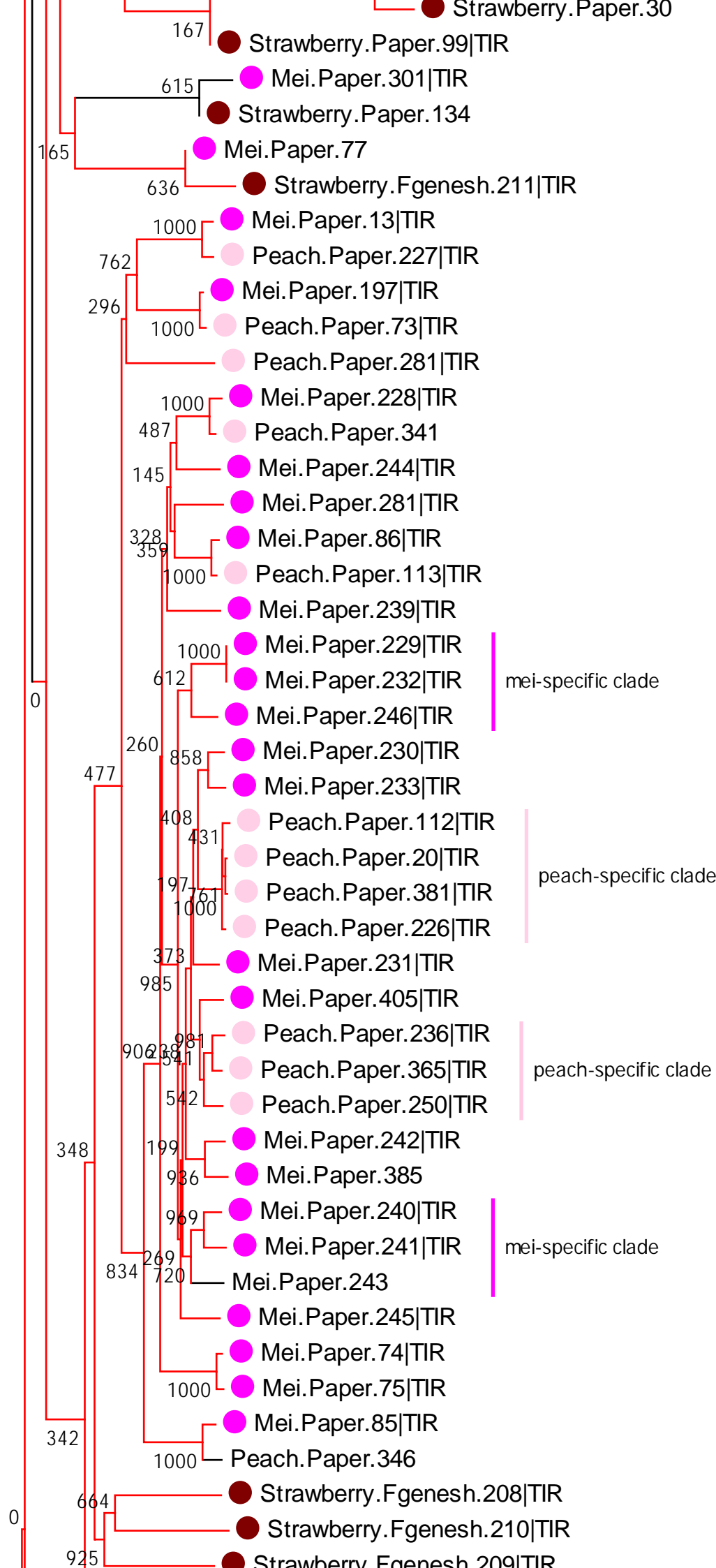

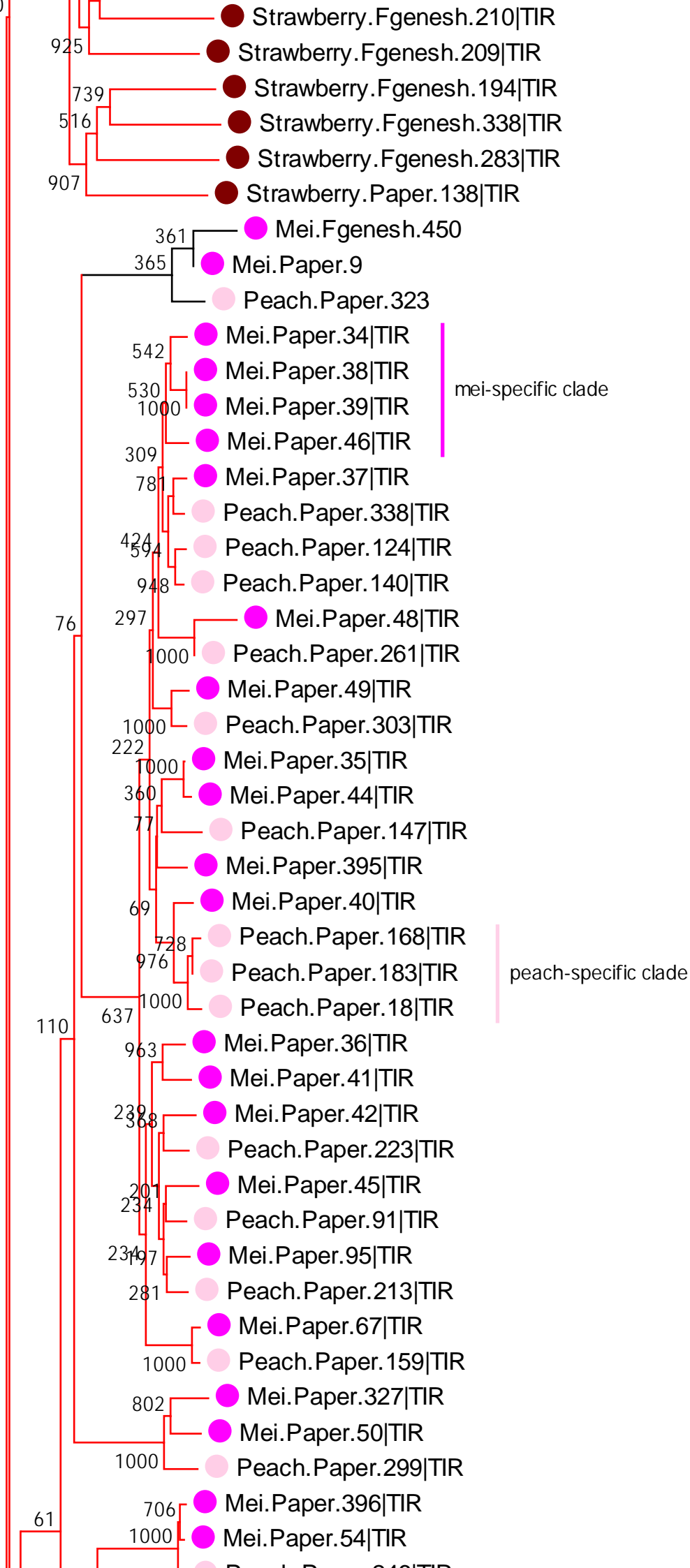

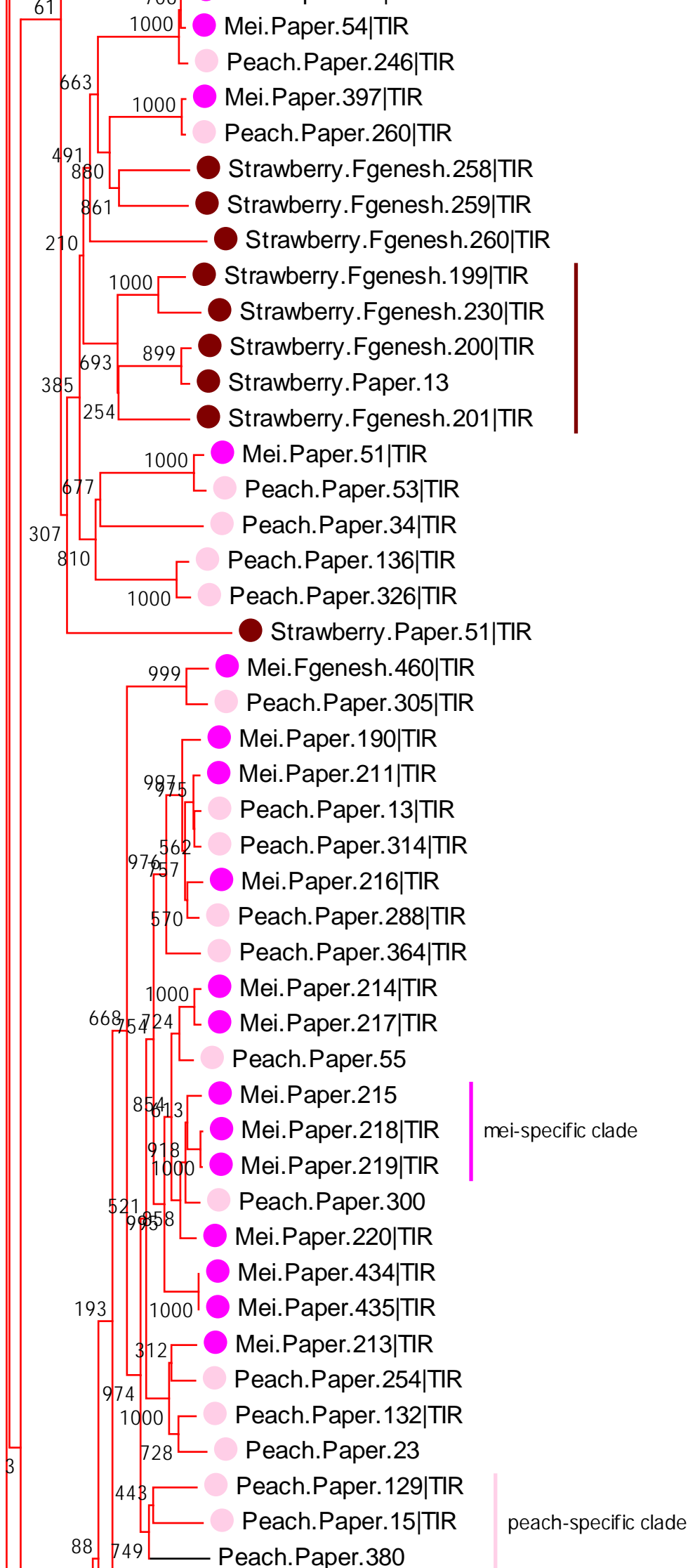

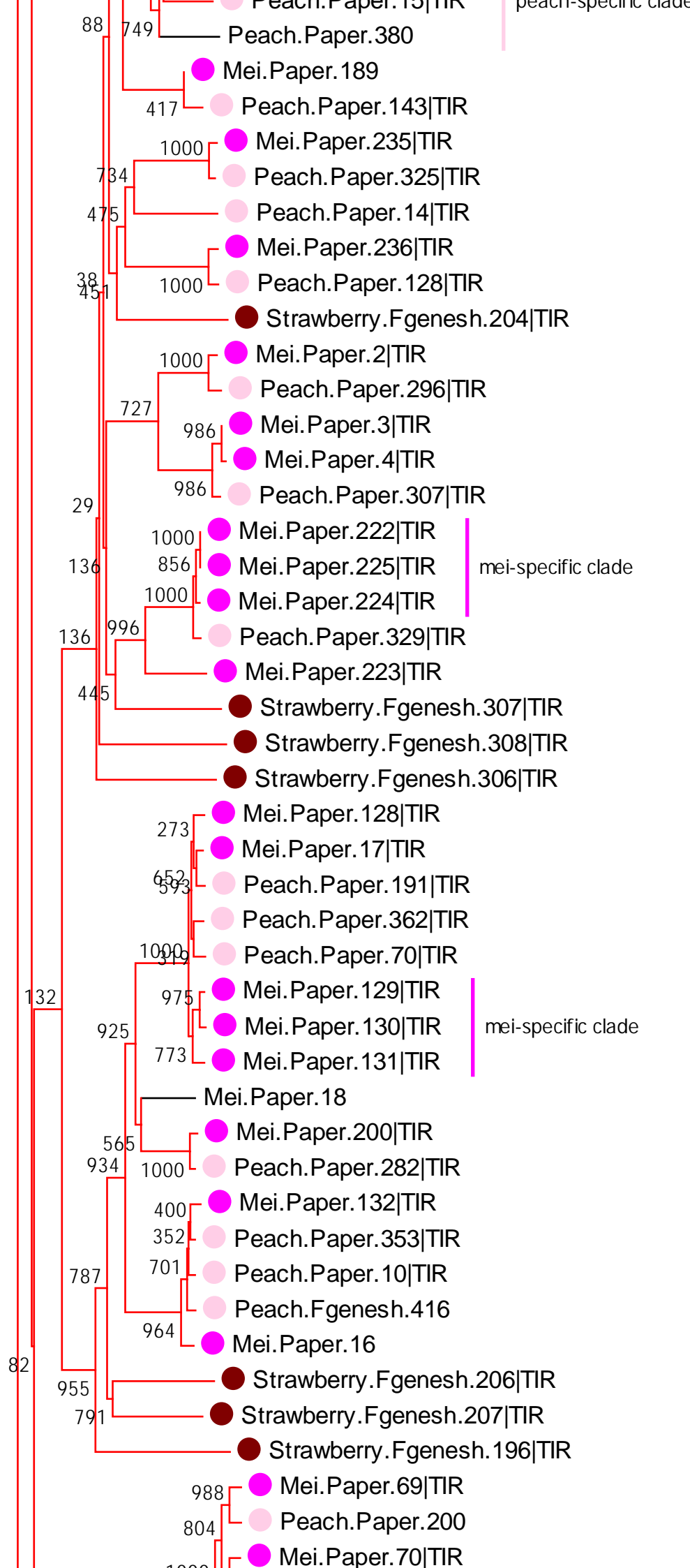

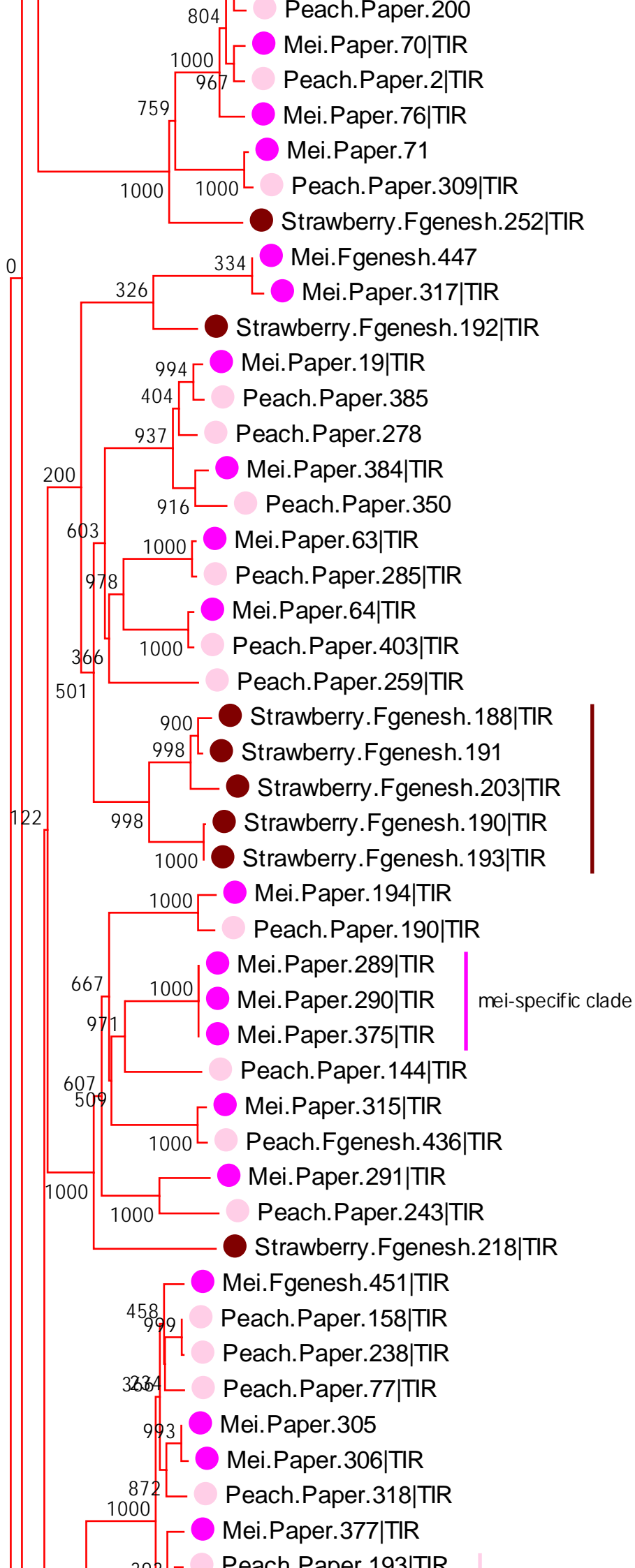

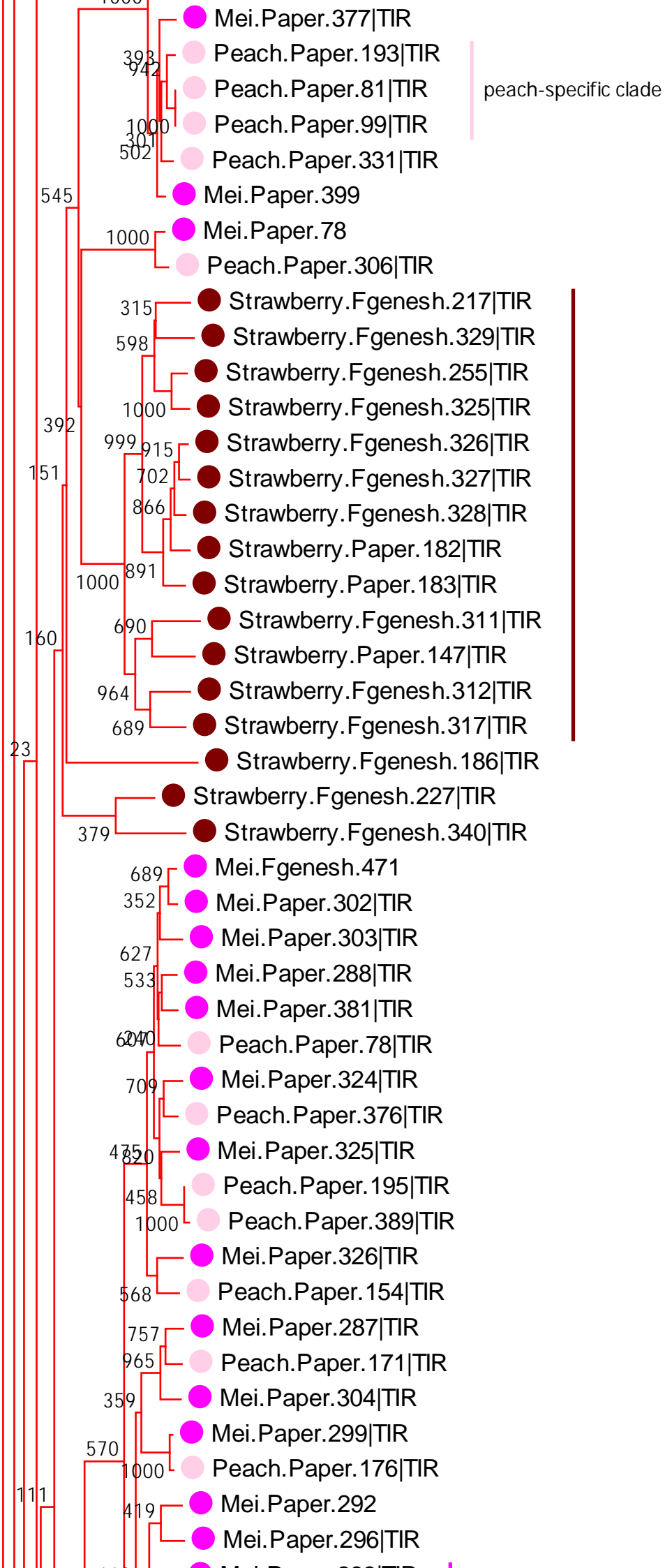

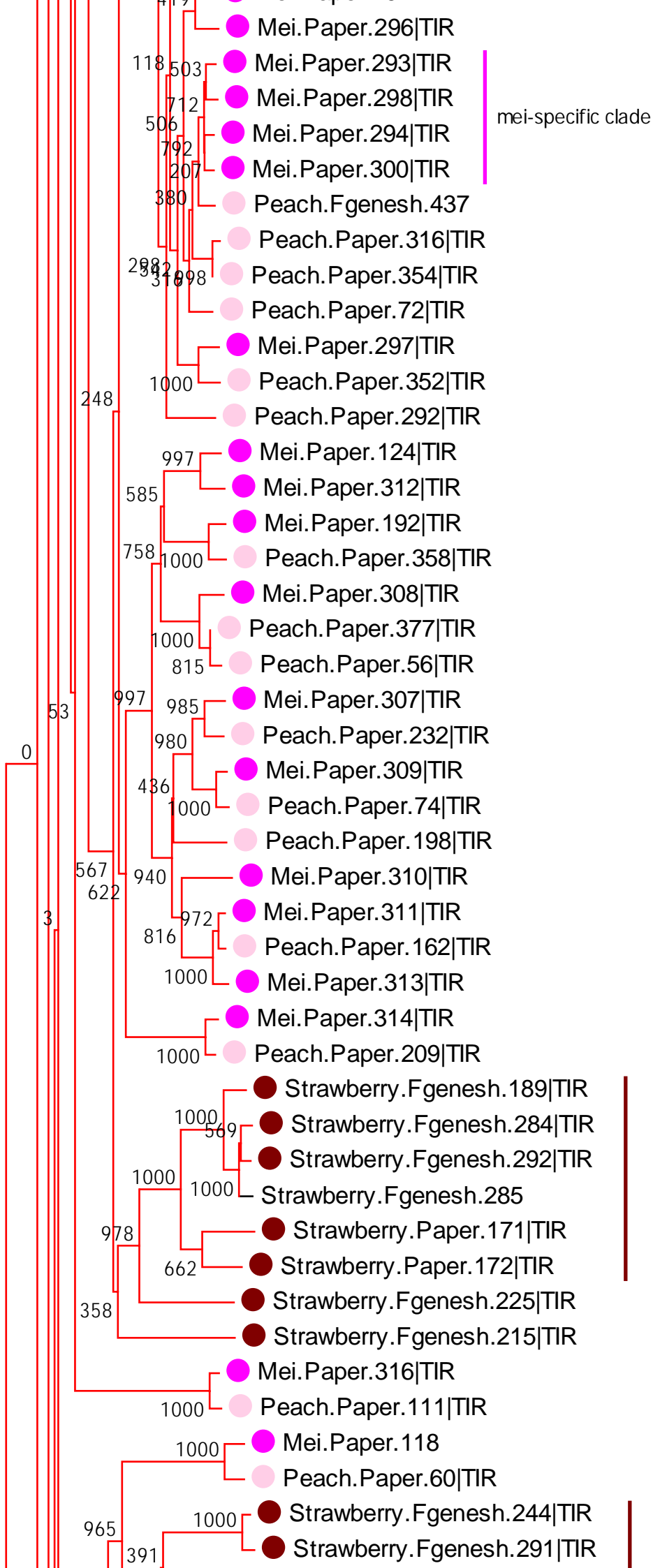

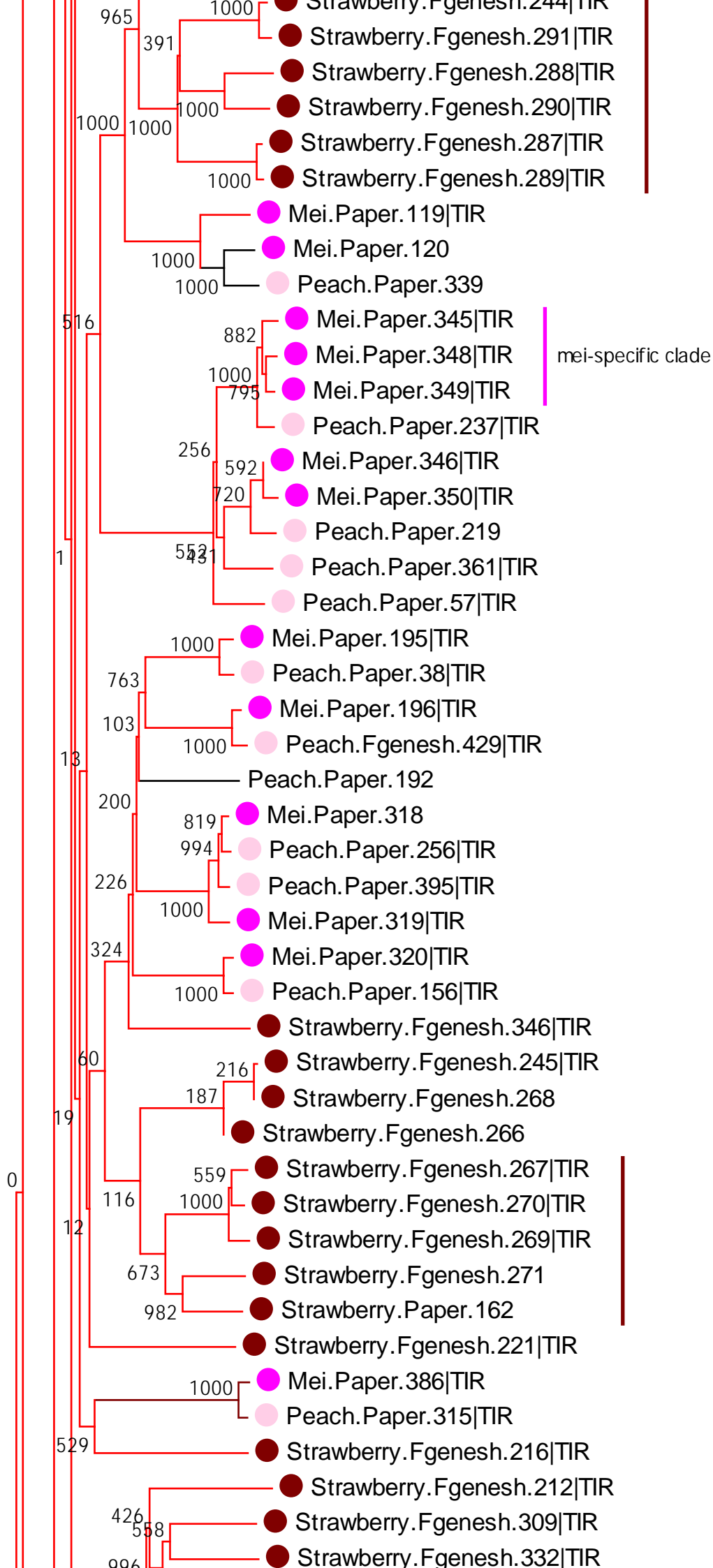

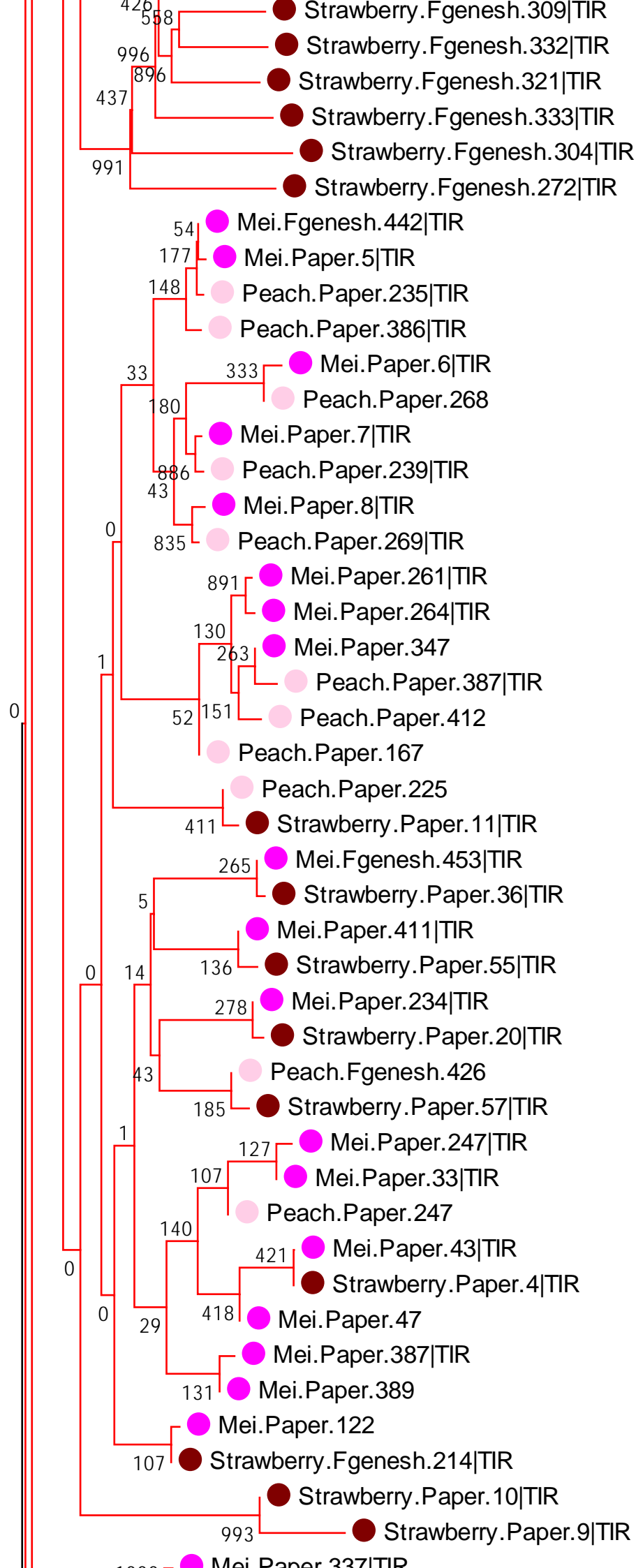

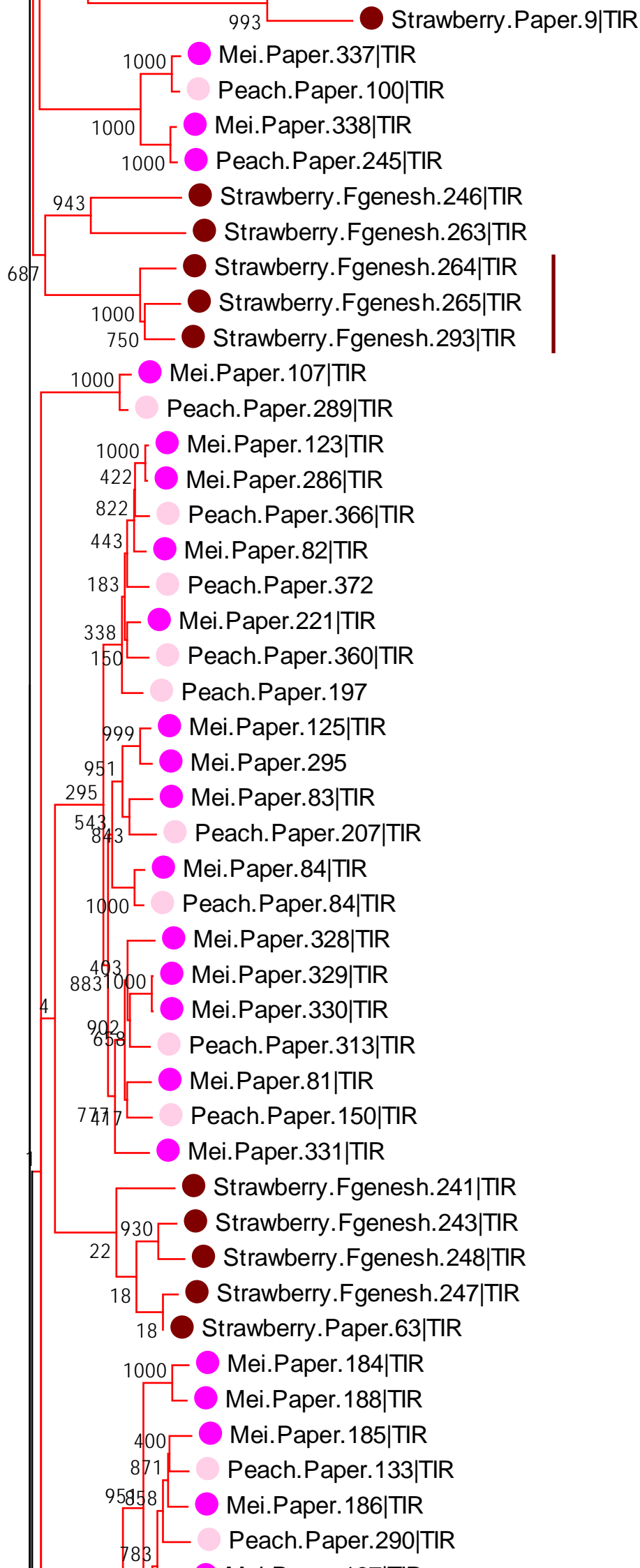

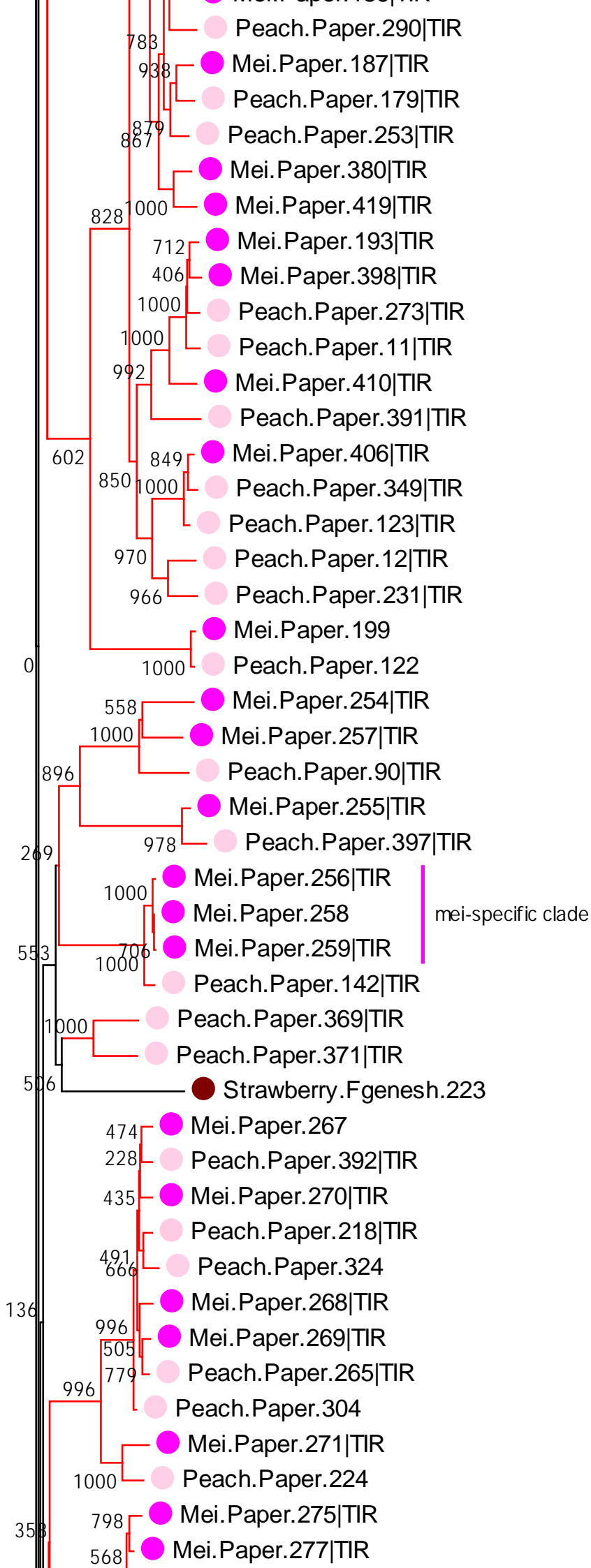

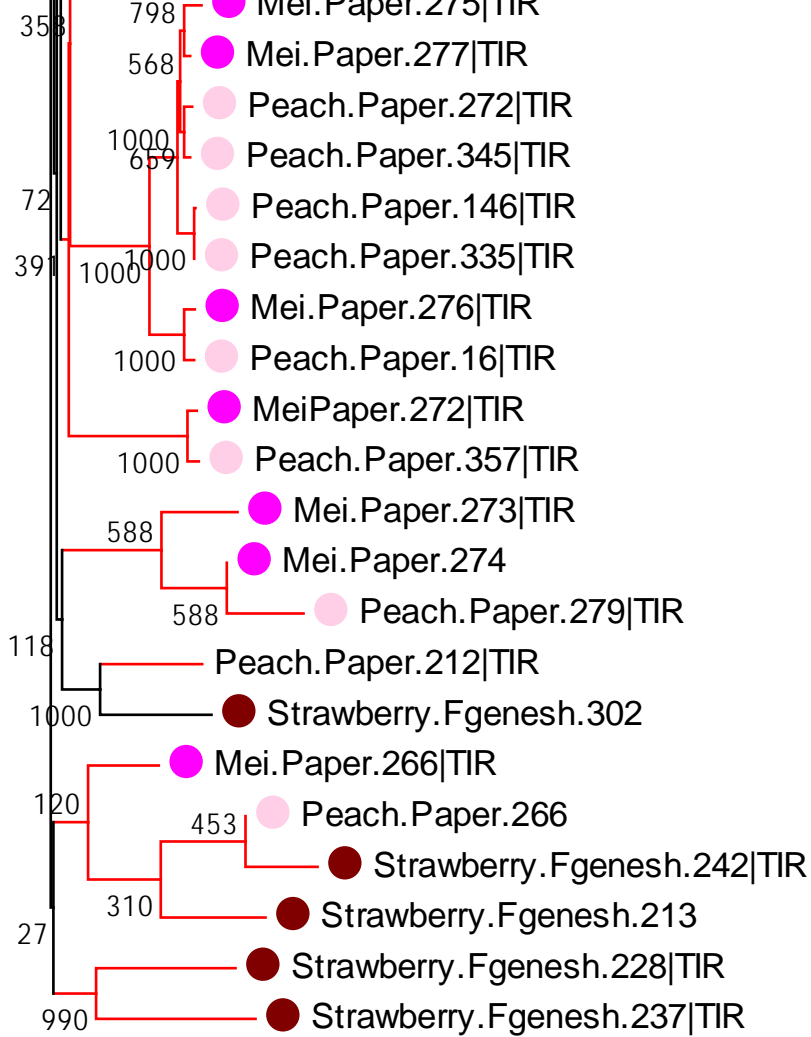

Supplement: Additional files 2: Figure S2. — Phylogenetic tree based on NBS domain of NBS-encoding genes in peach, mei and strawberry. Red lines represent TIR genes and black lines represent non-TIR genes. NBS-encoding genes in peach, mei and strawberry are shown as pink circles, purple circles, and dark green circles, respectively. The vertical bars with different colors beside the tree are used to represent species-specific gene clades of three different species. [file 12863_2015_208_MOESM2_ESM.pdf]

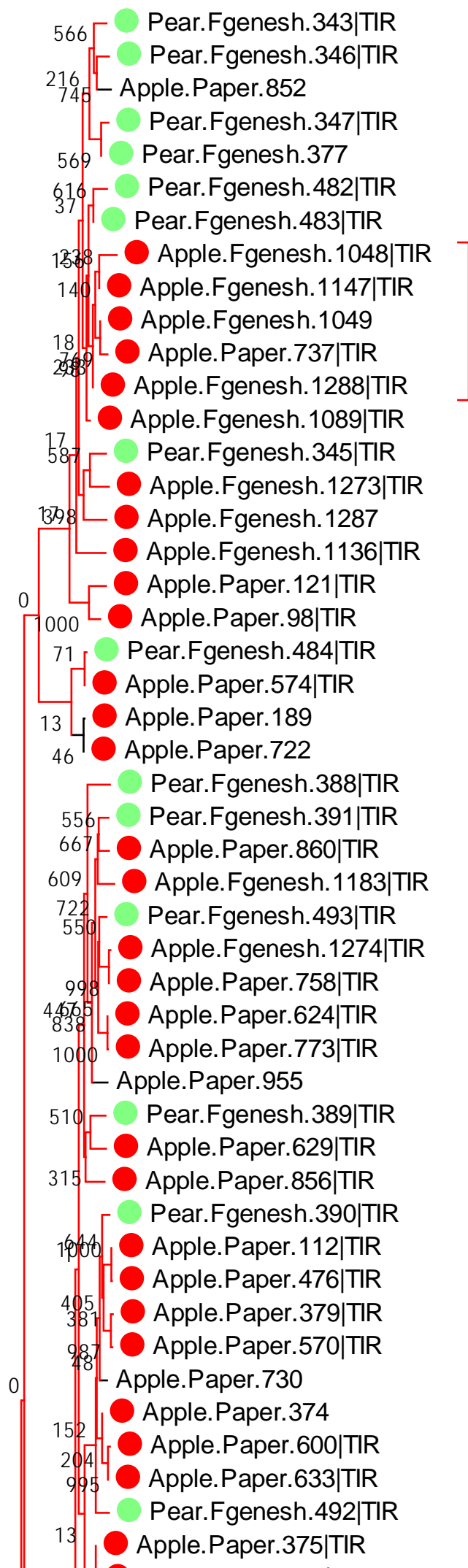

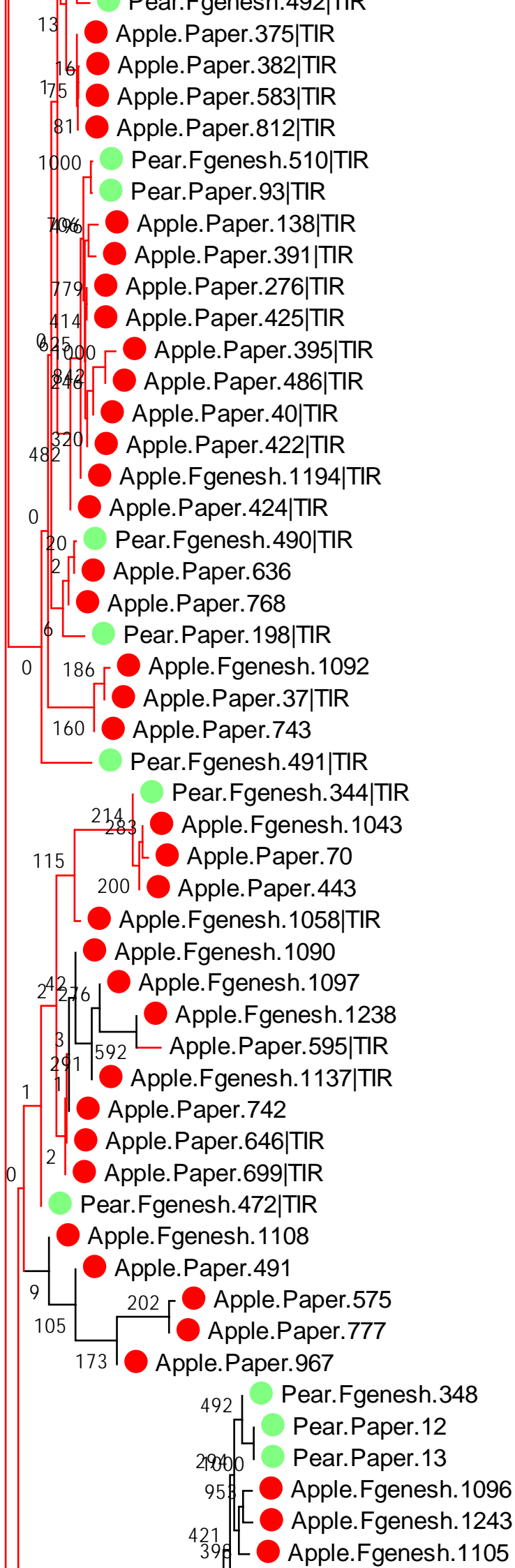

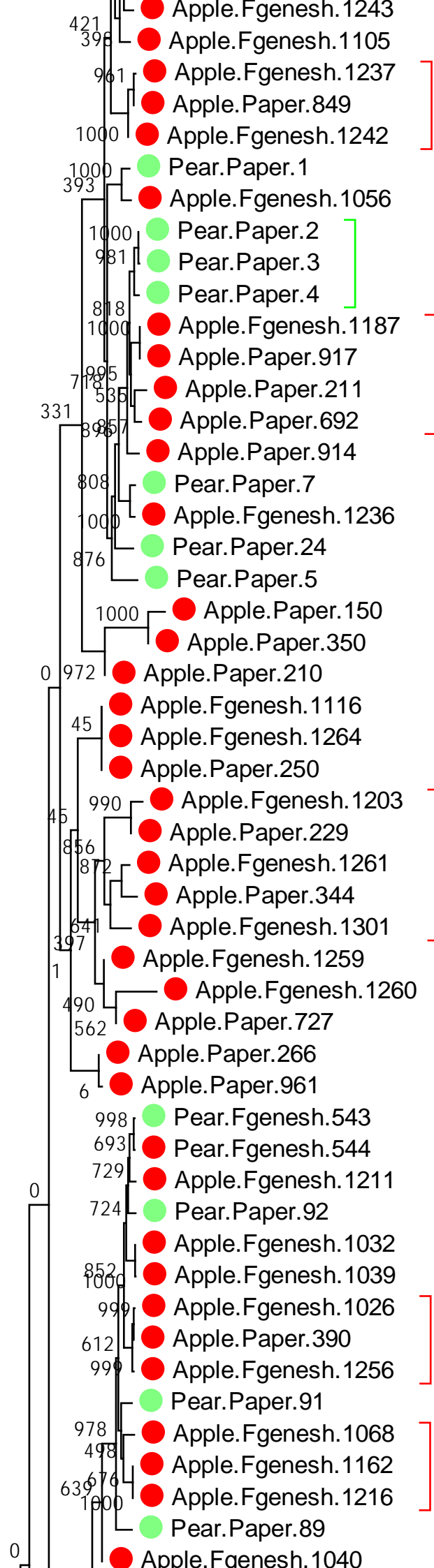

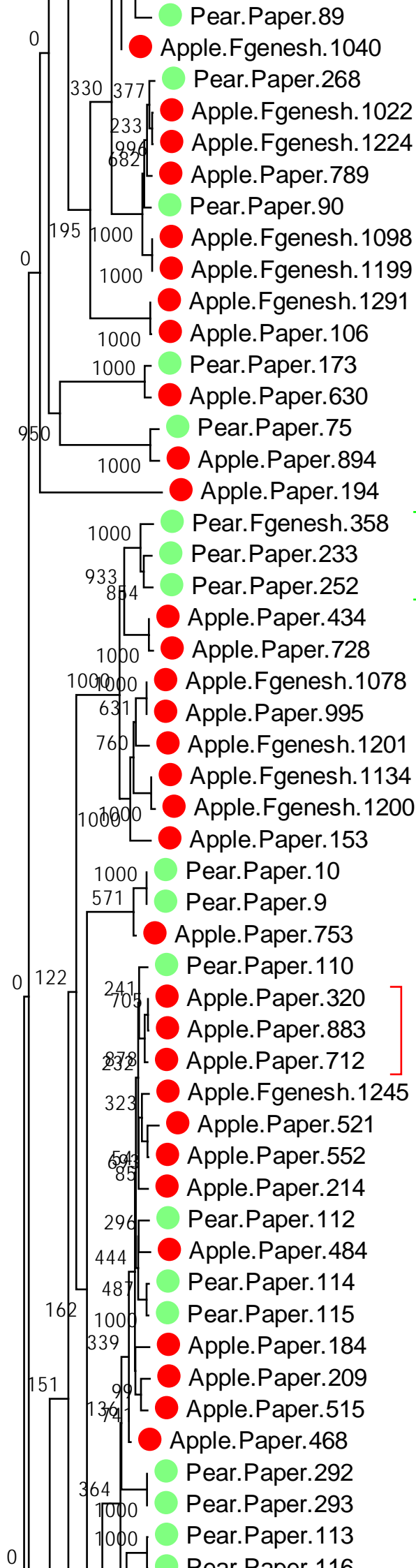

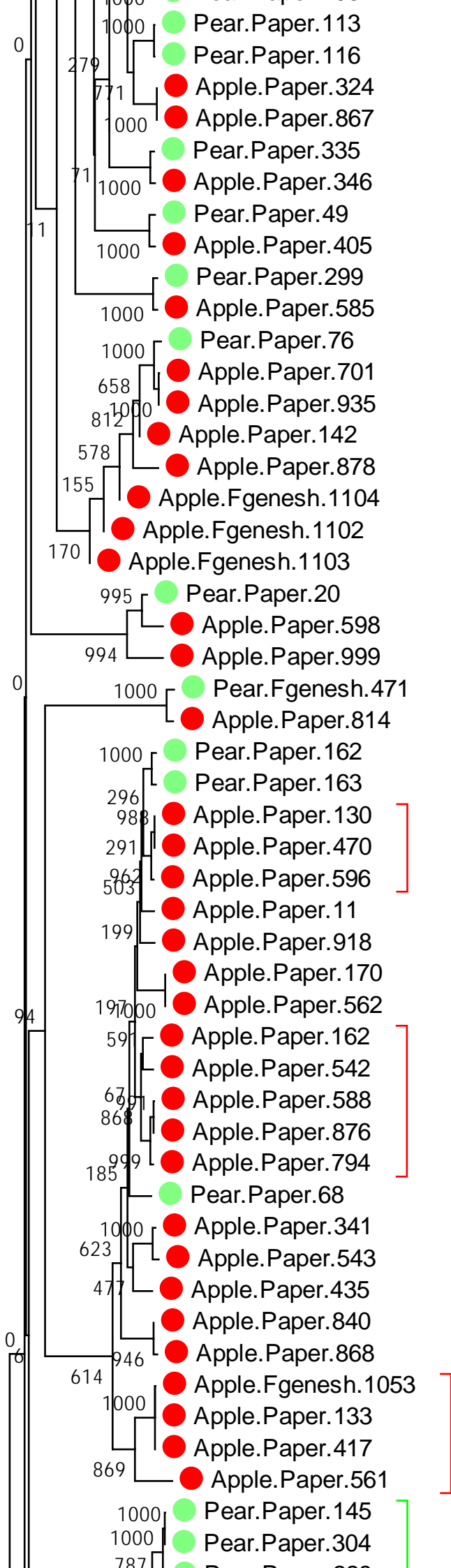

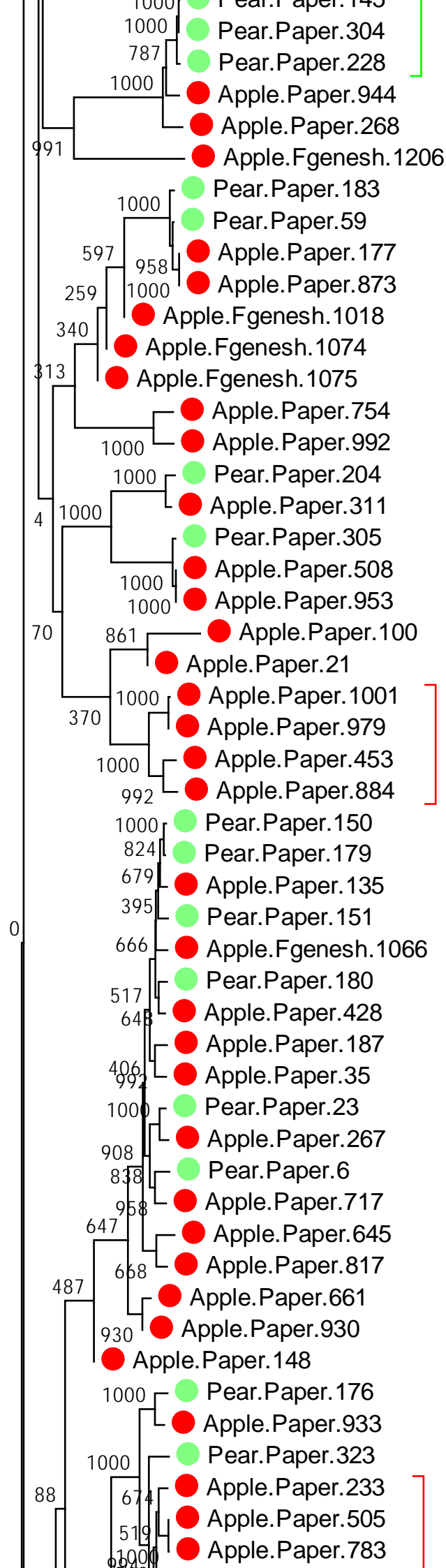

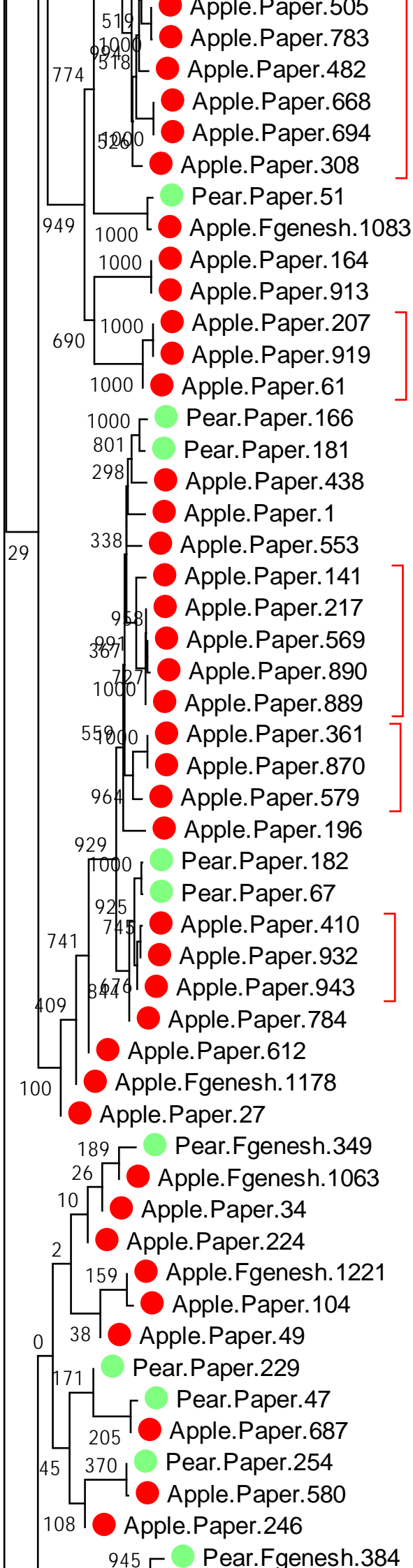

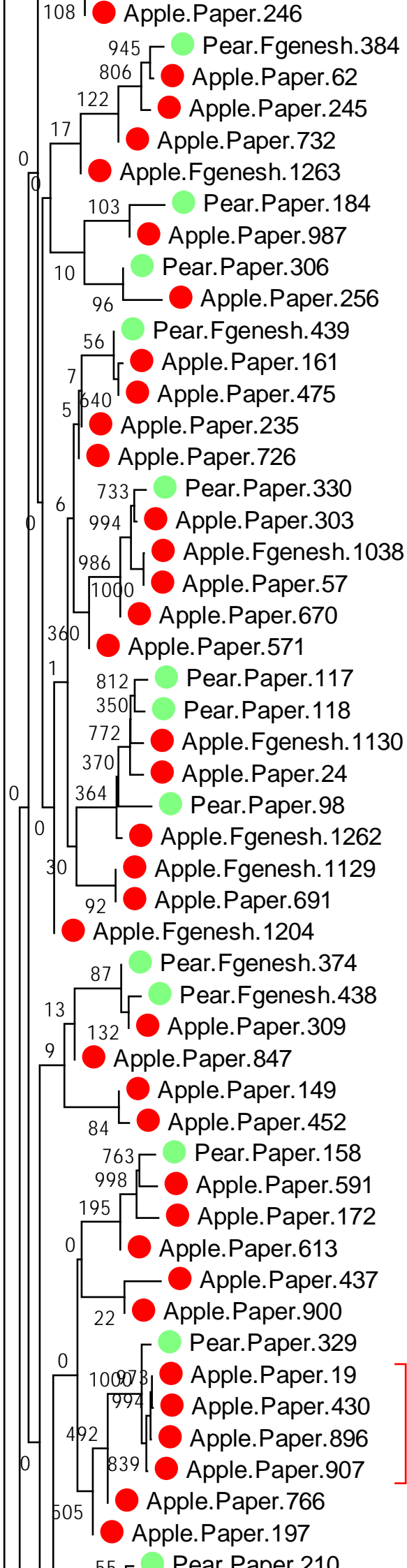

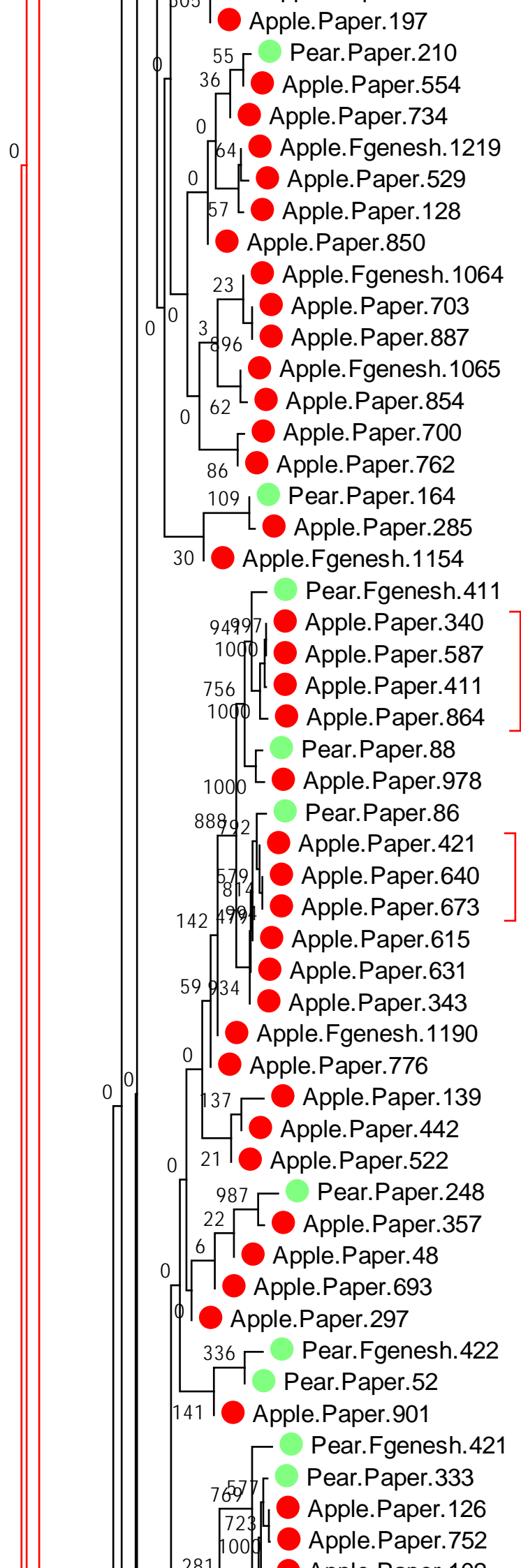

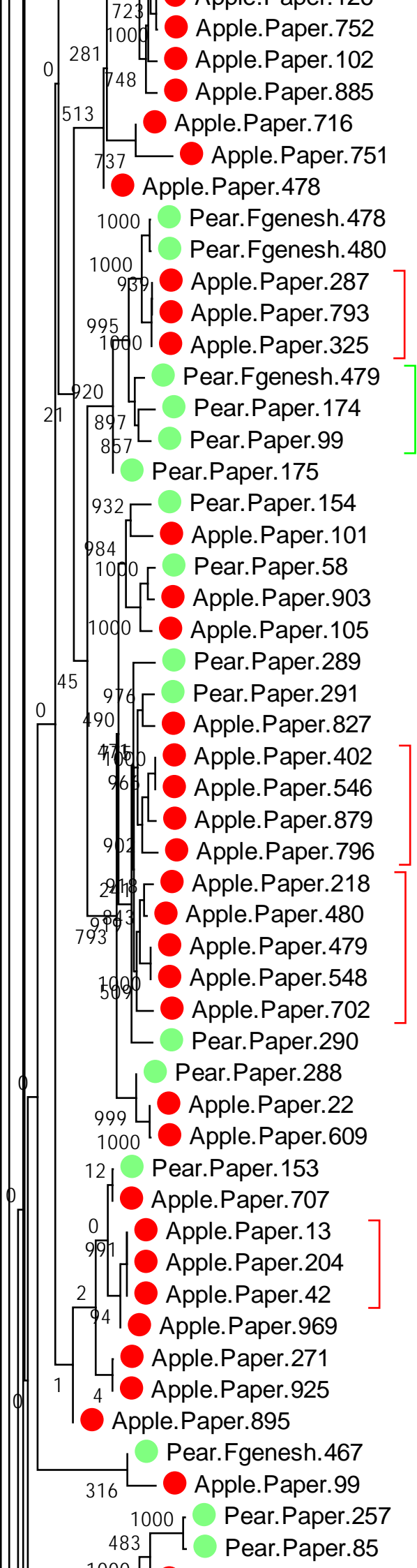

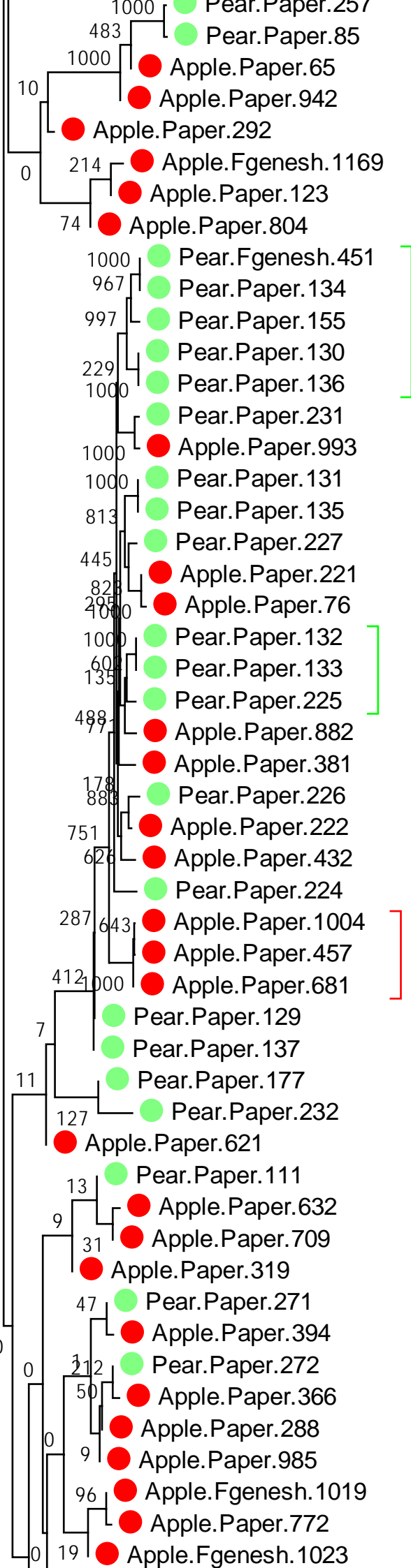

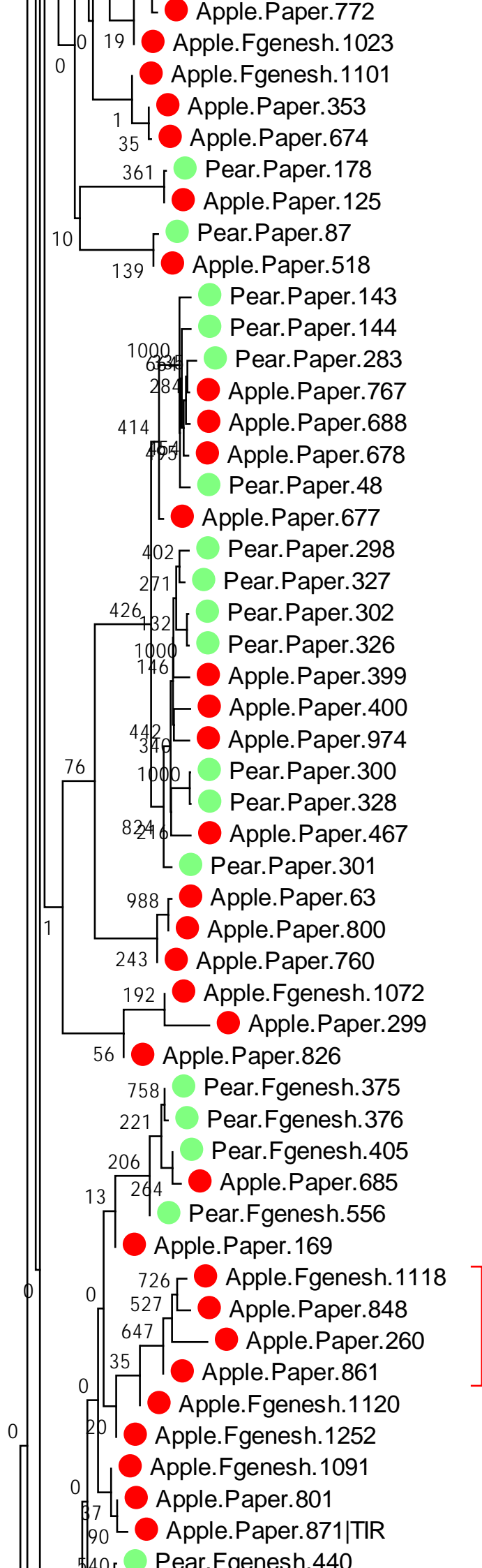

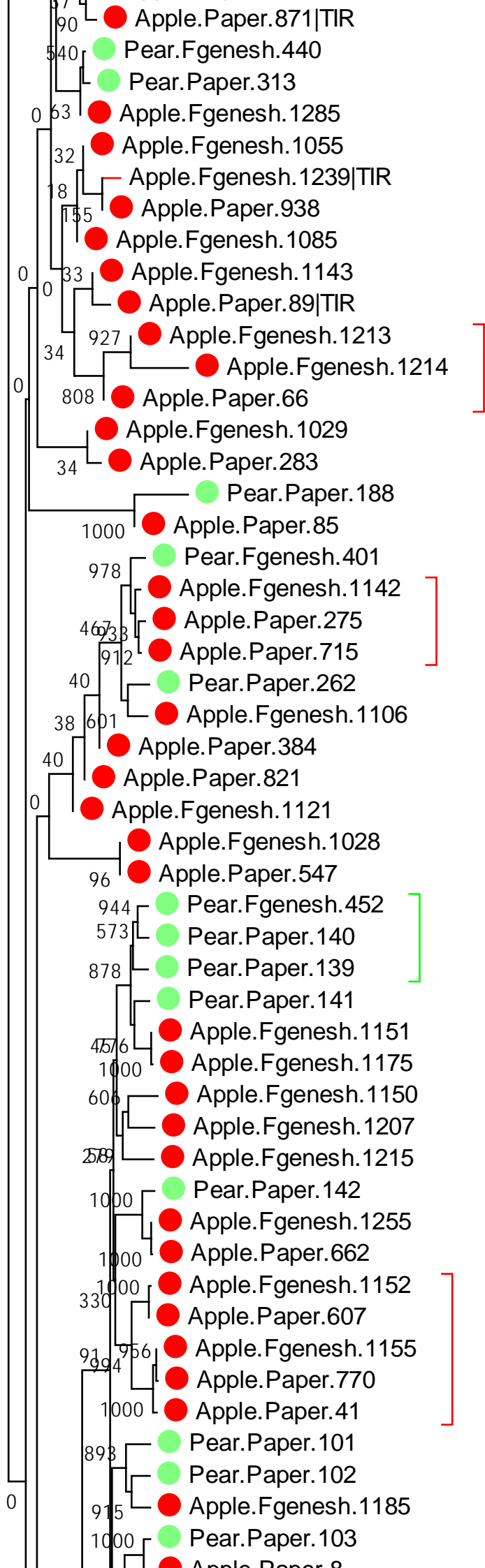

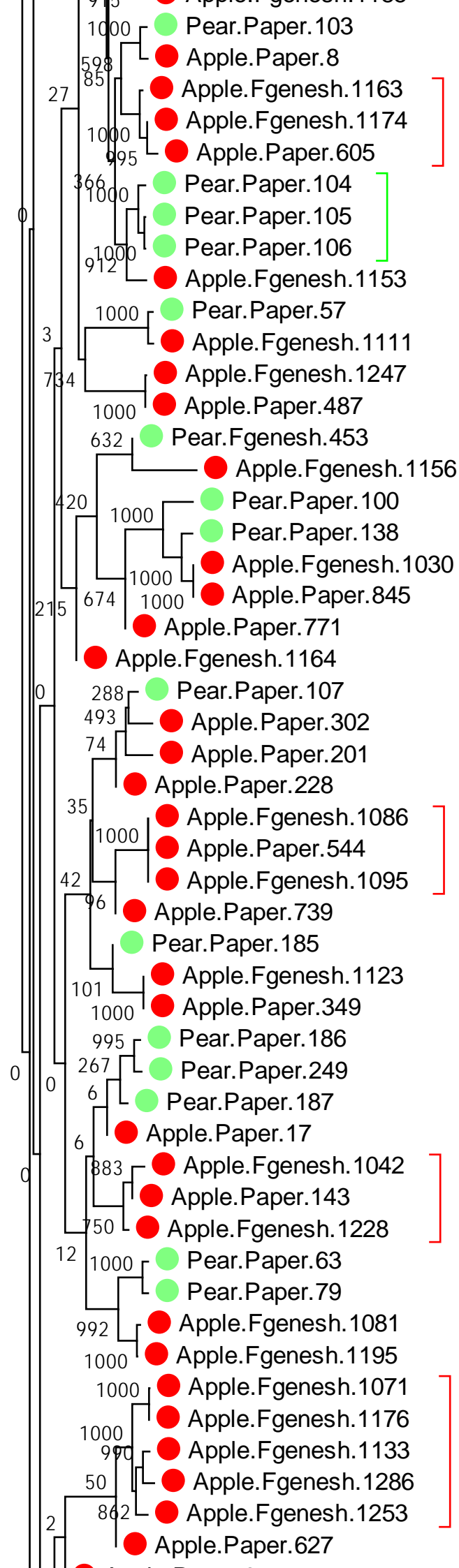

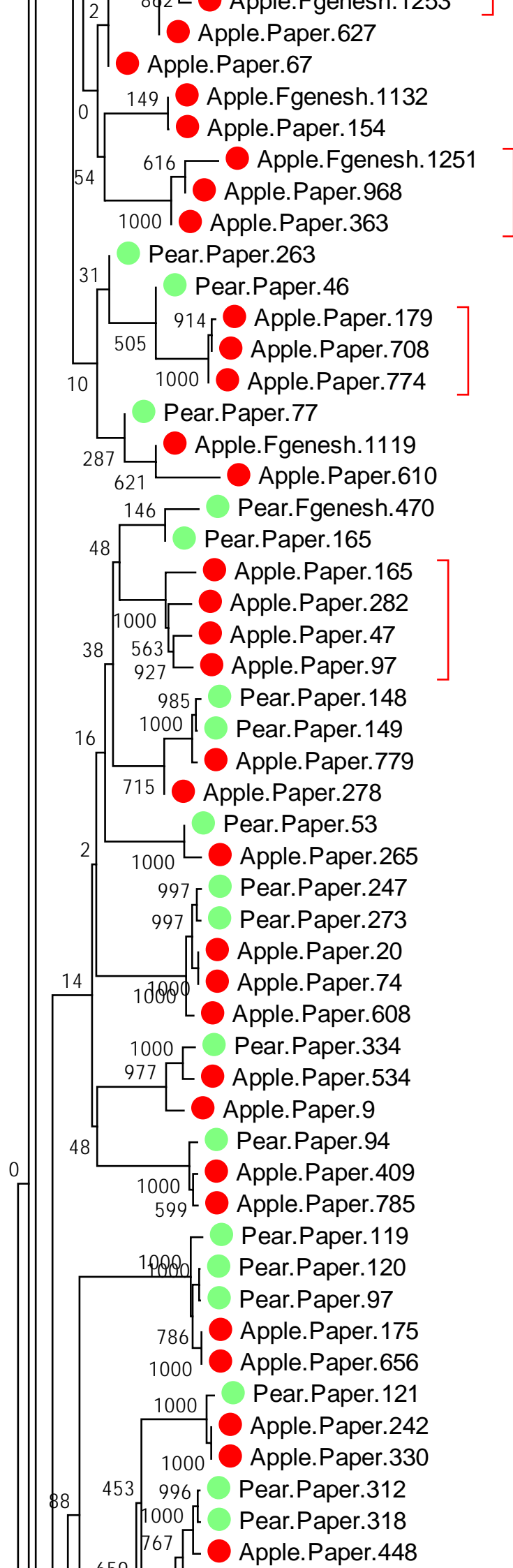

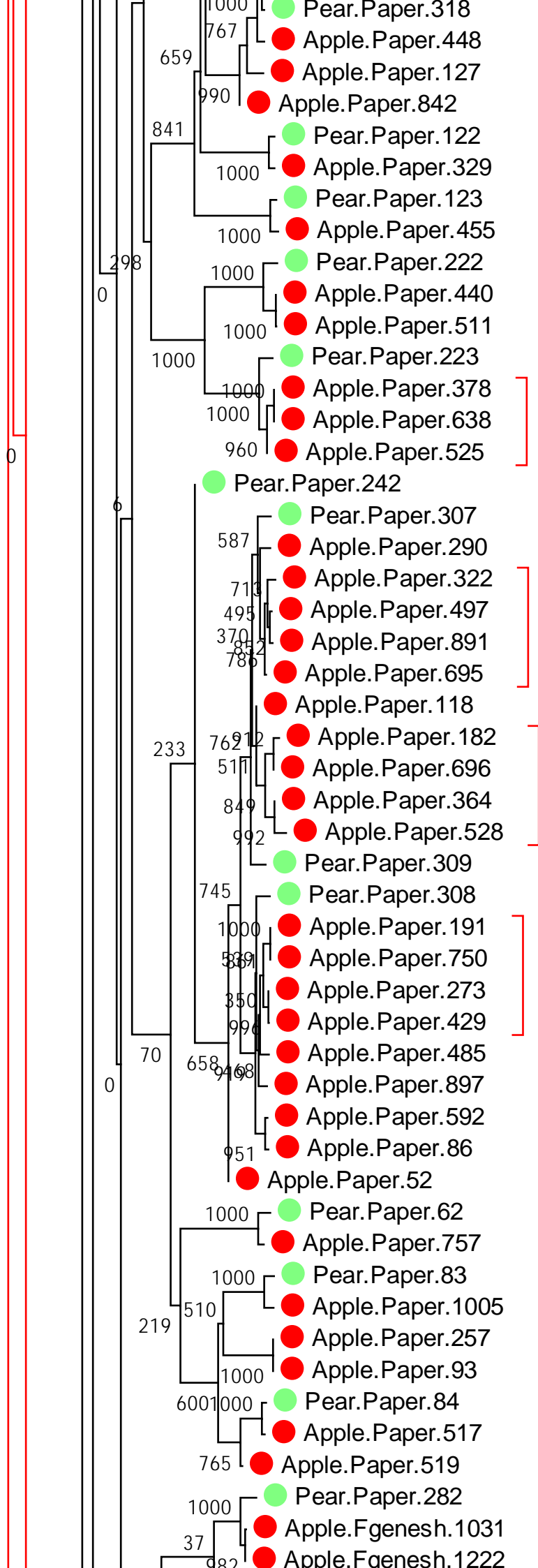

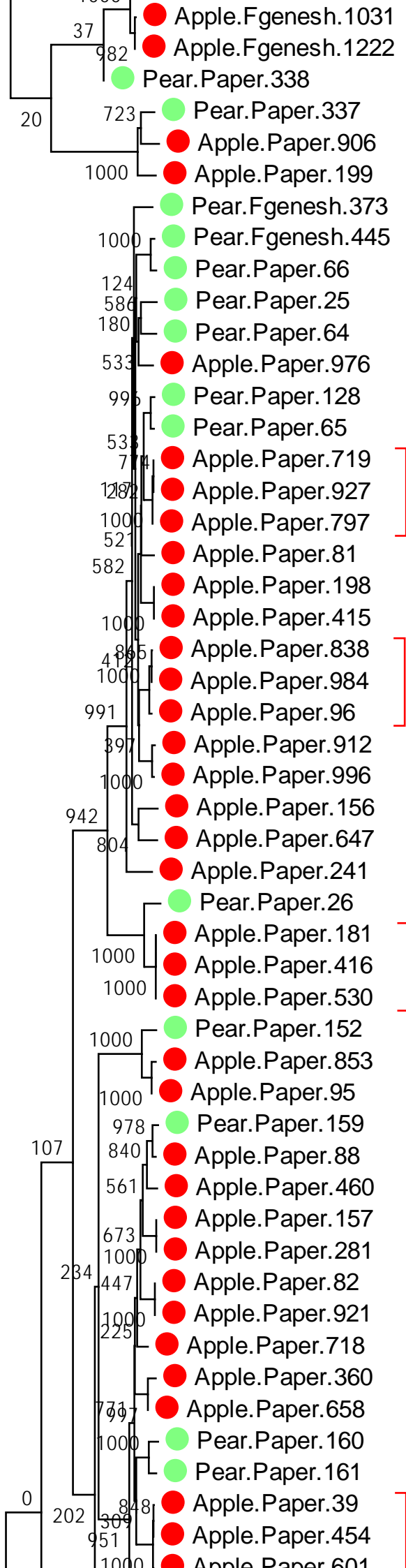

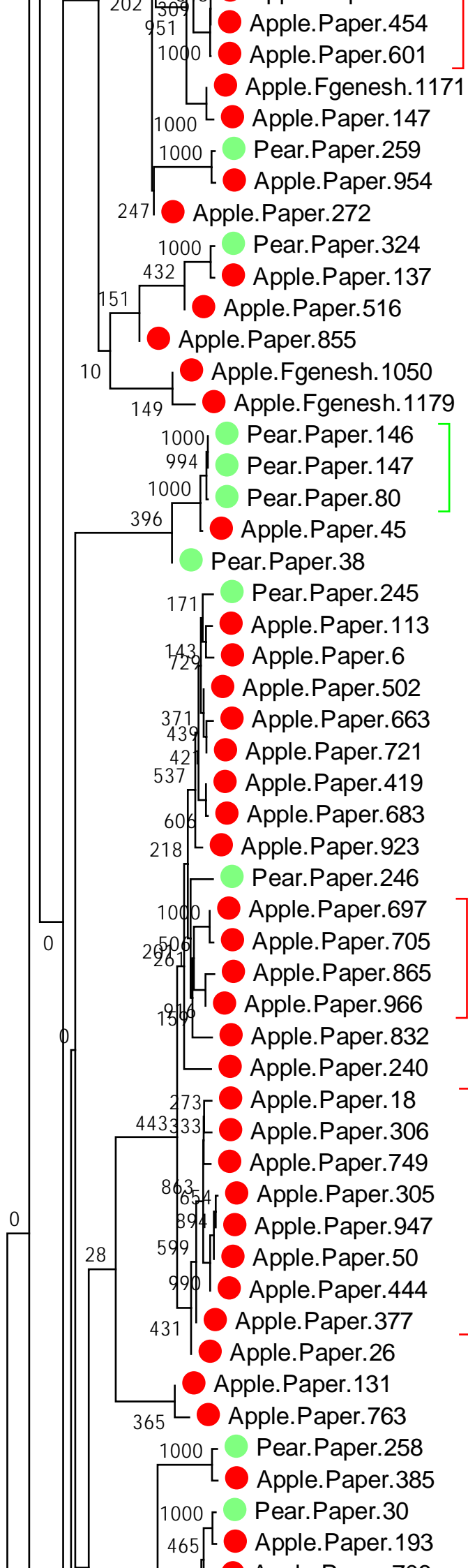

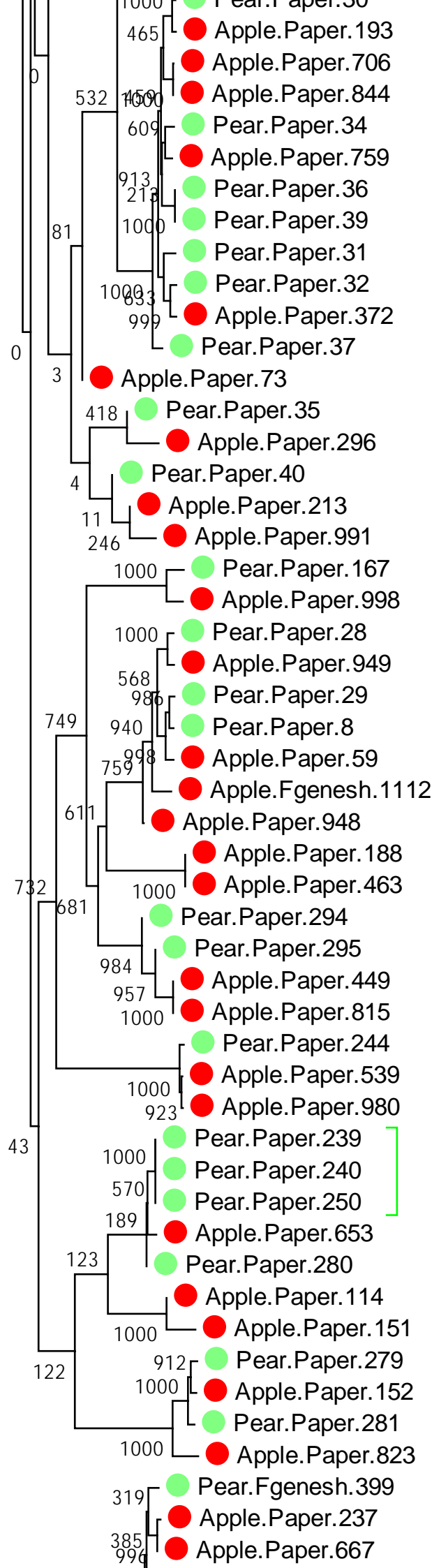

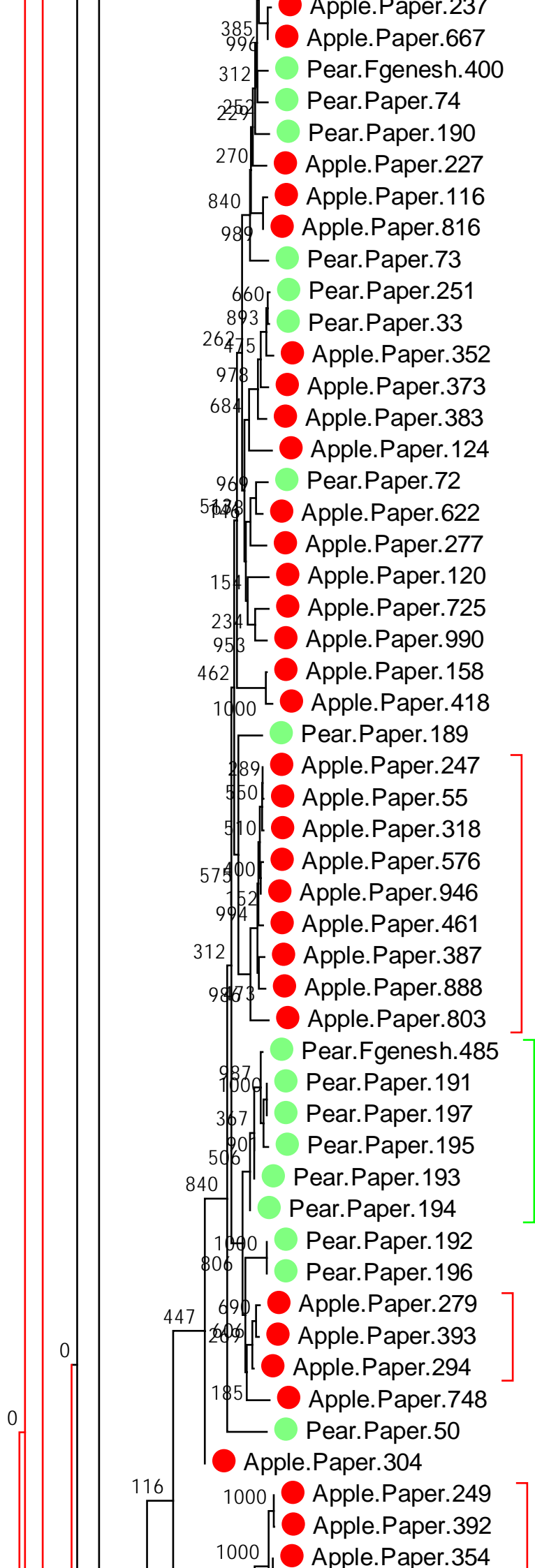

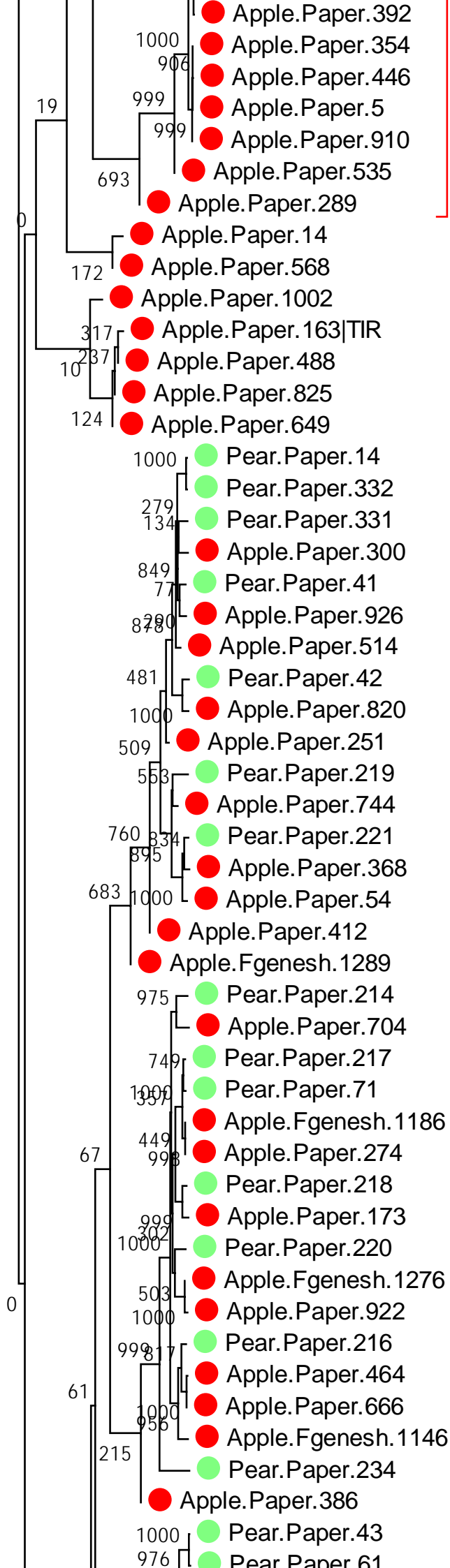

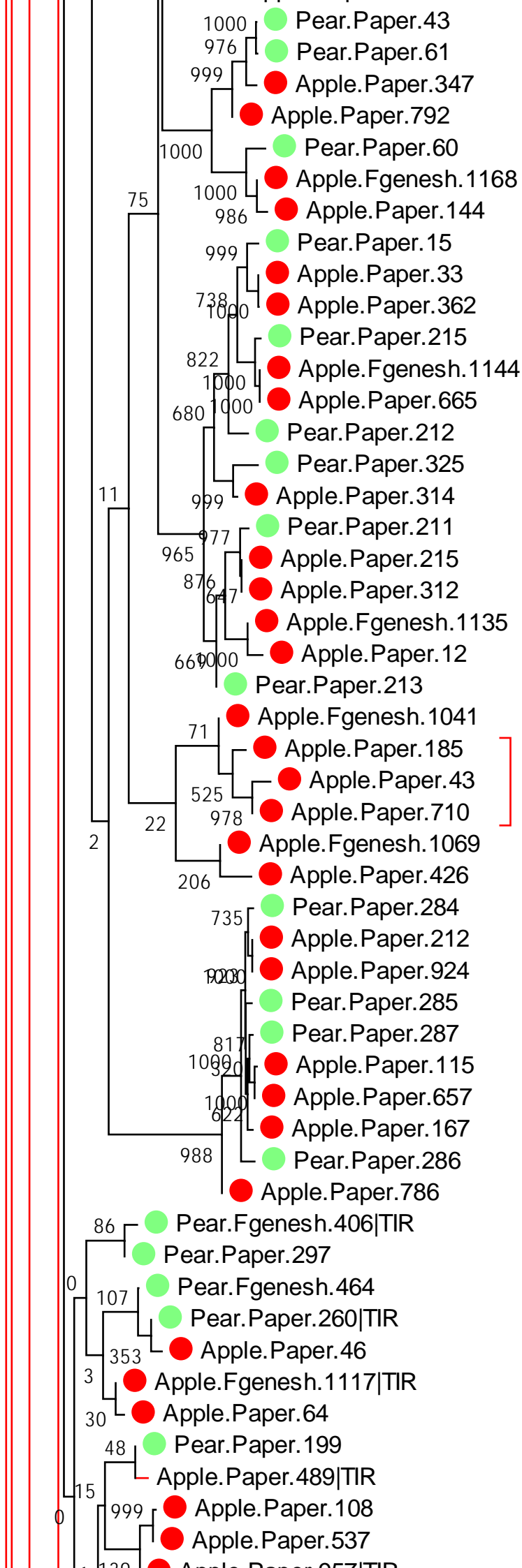

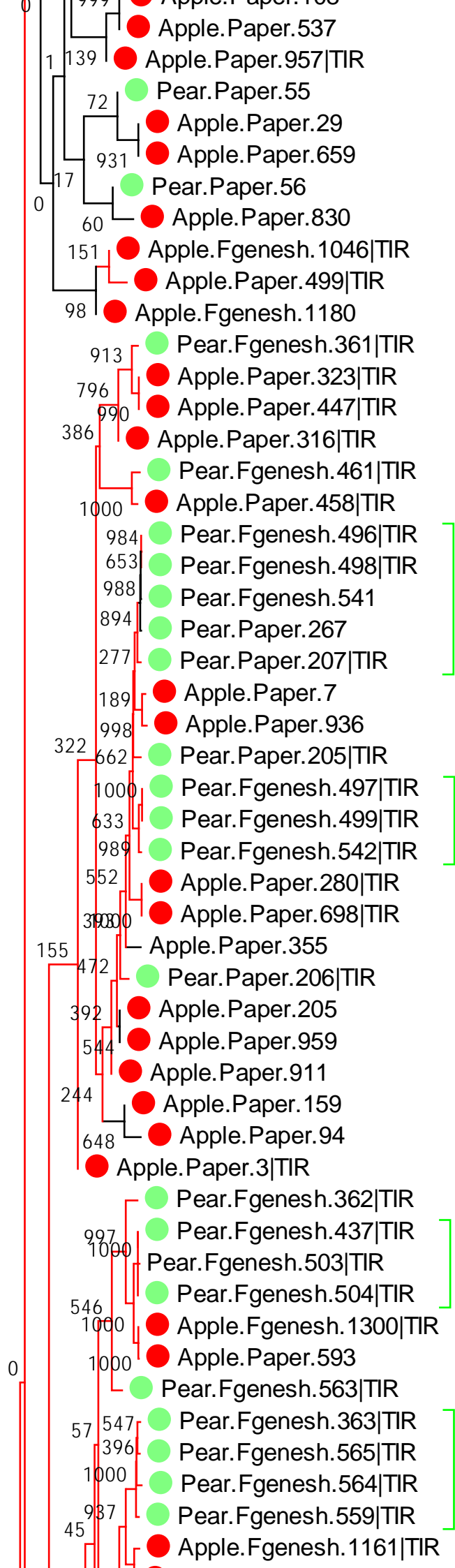

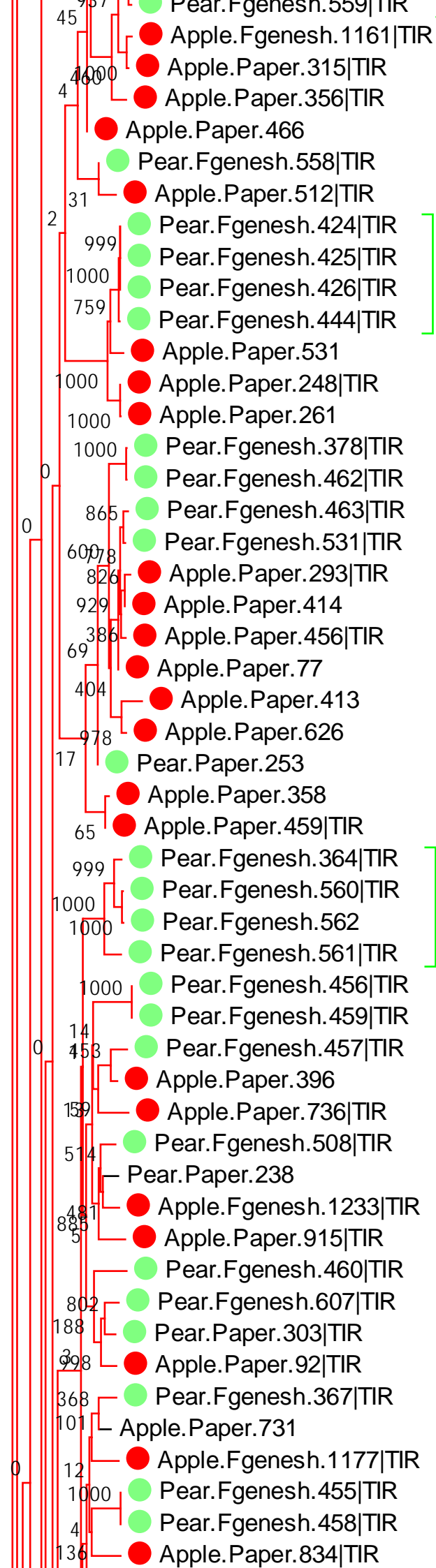

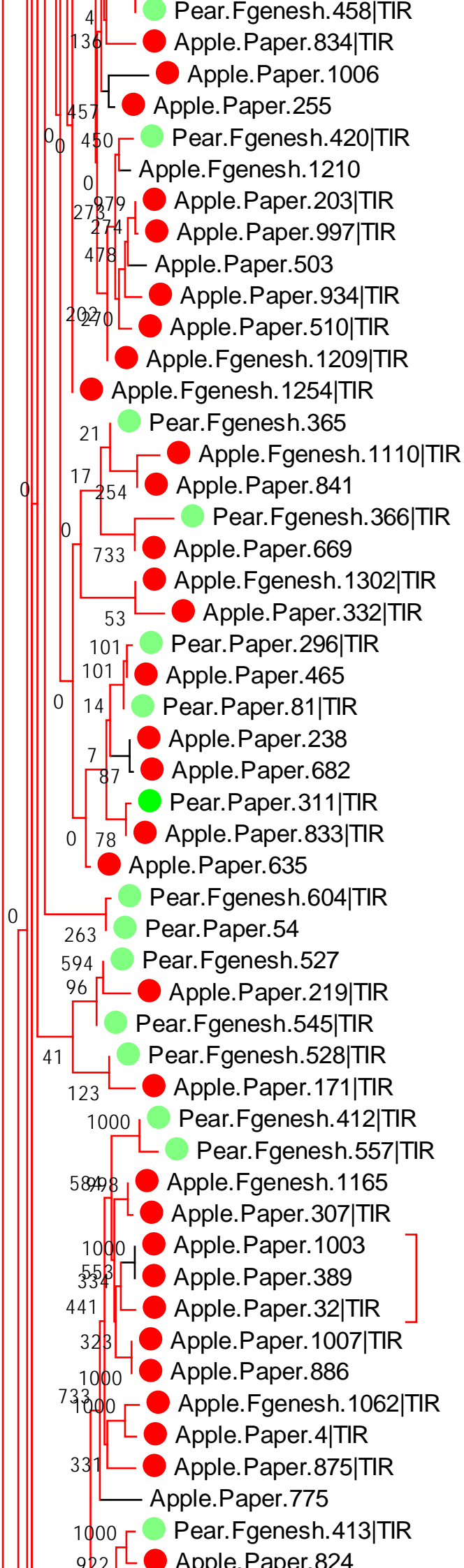

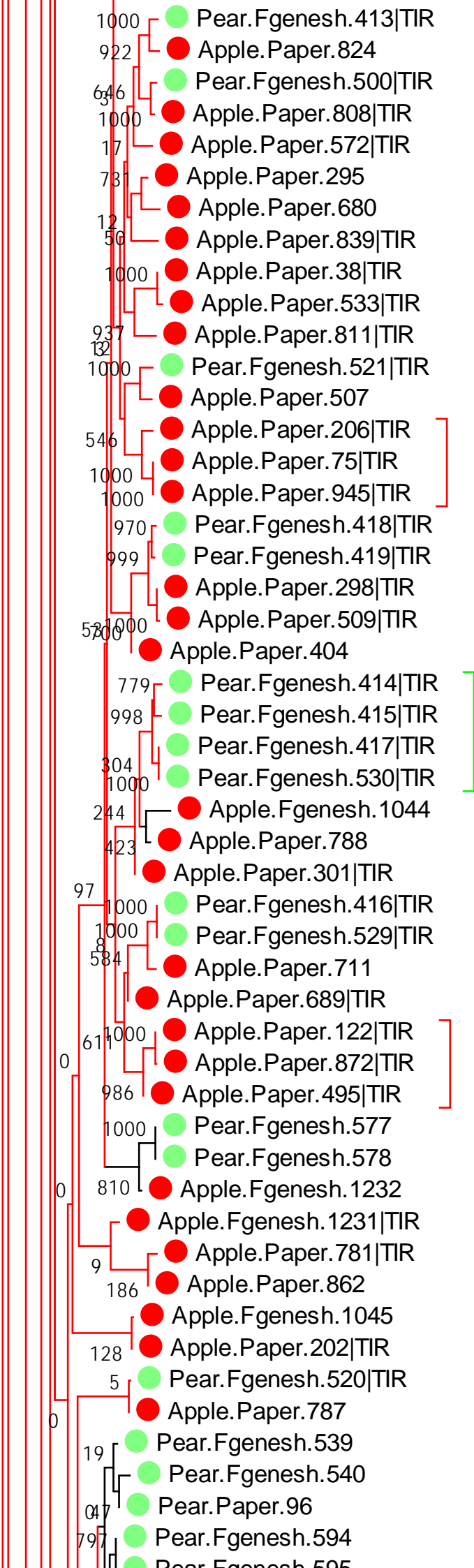

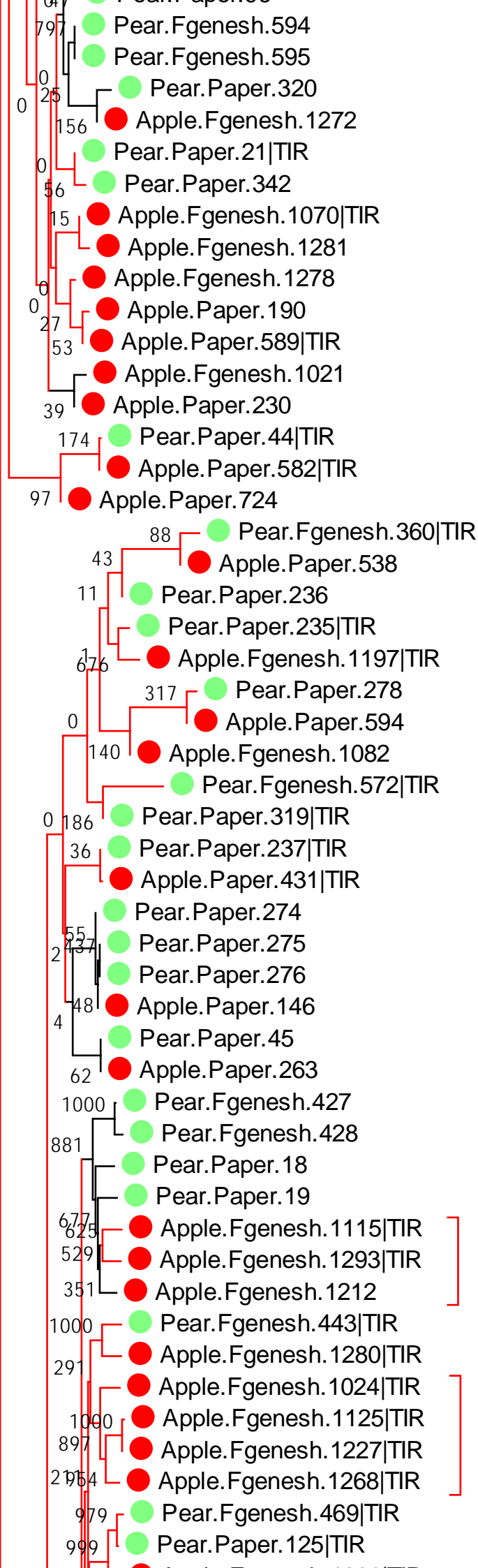

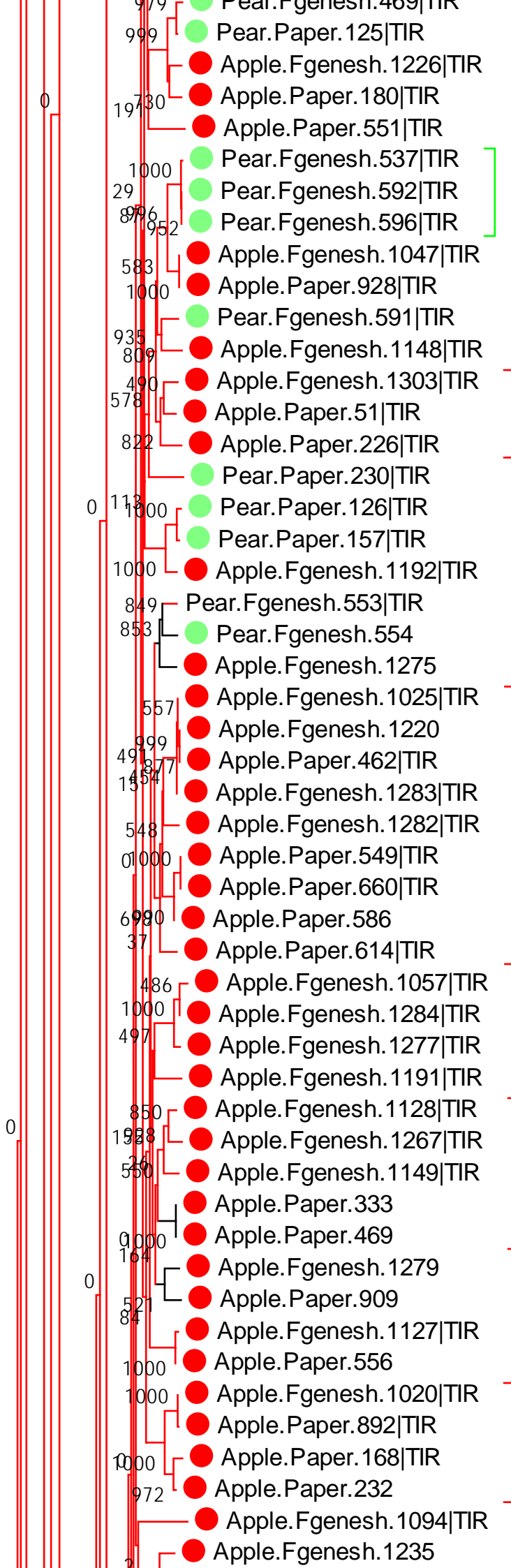

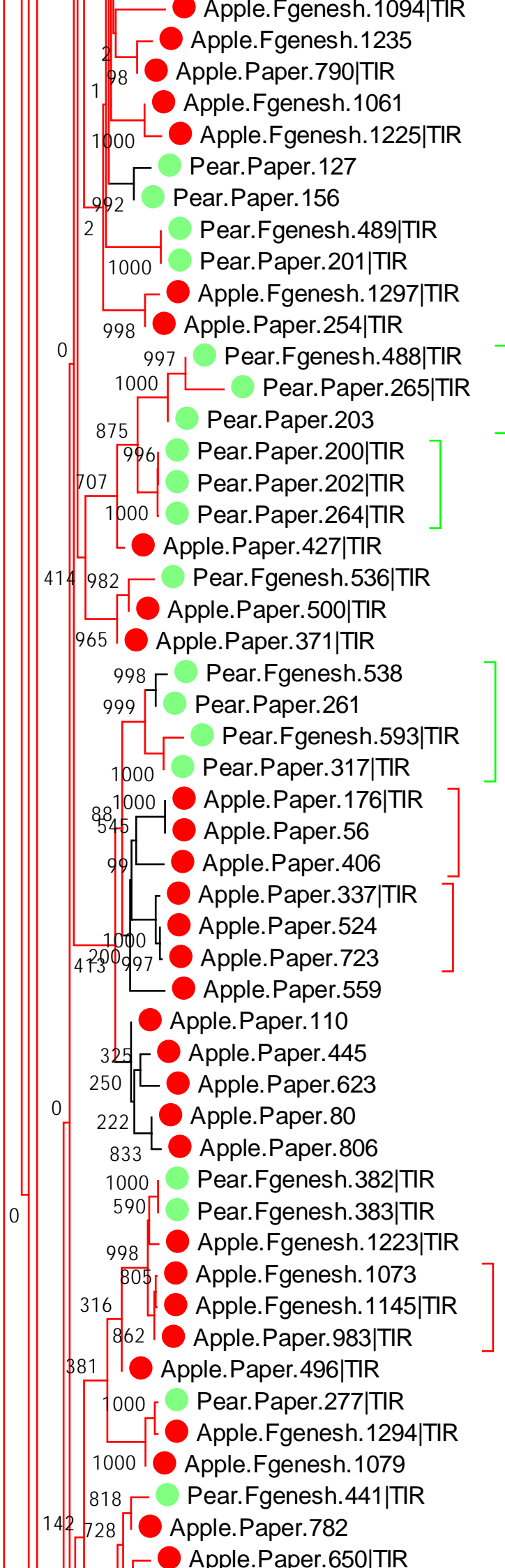

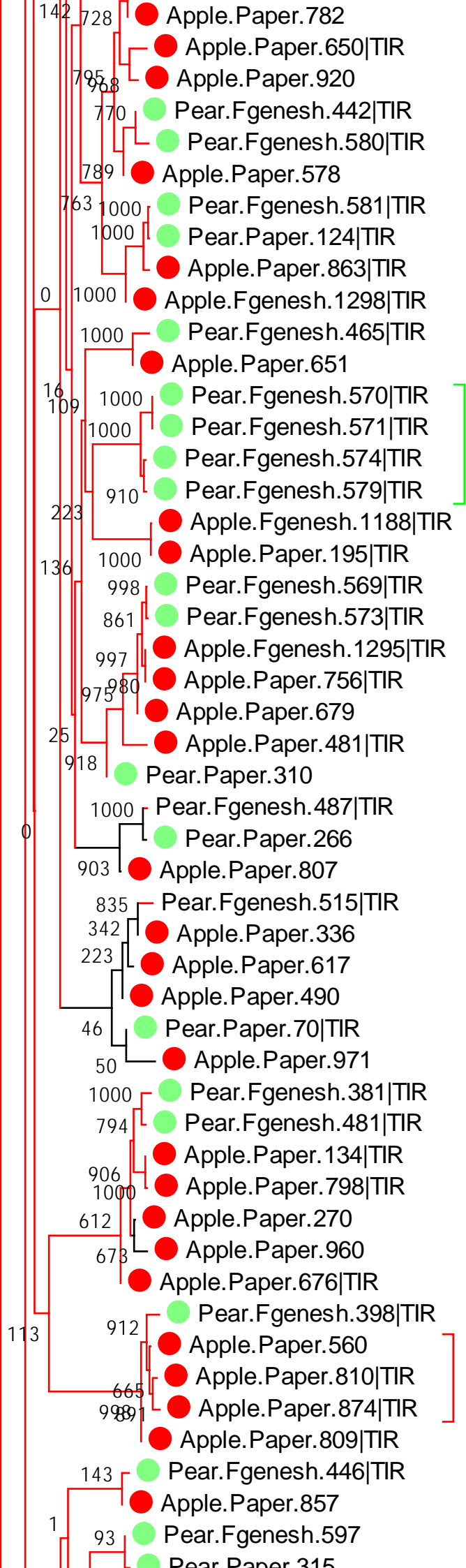

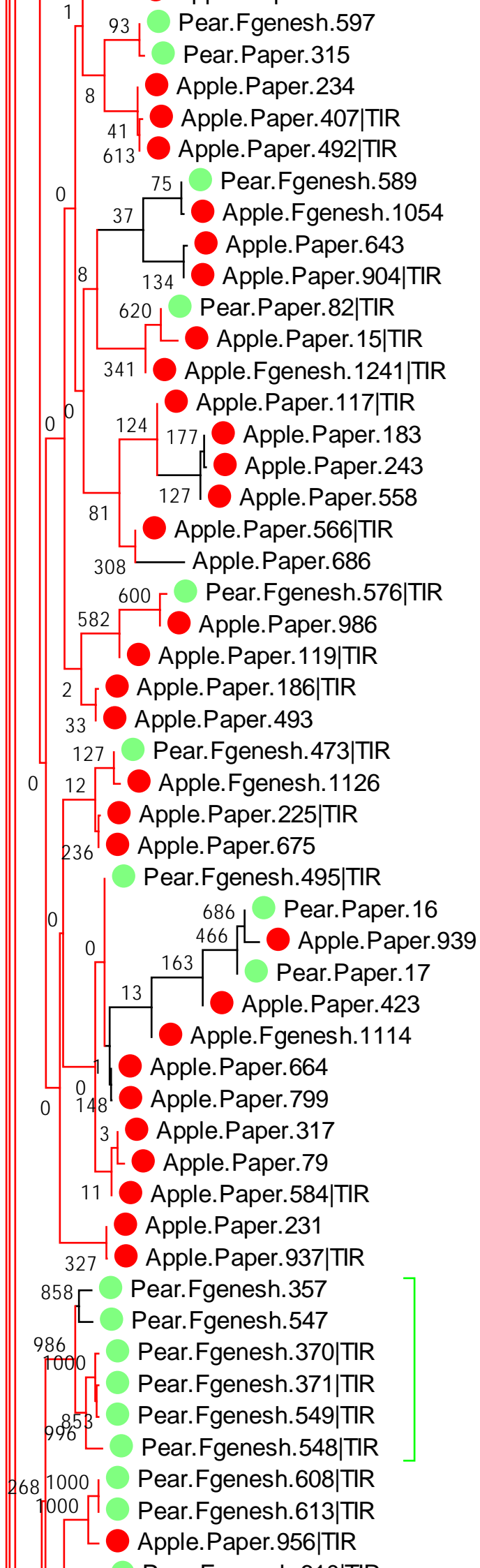

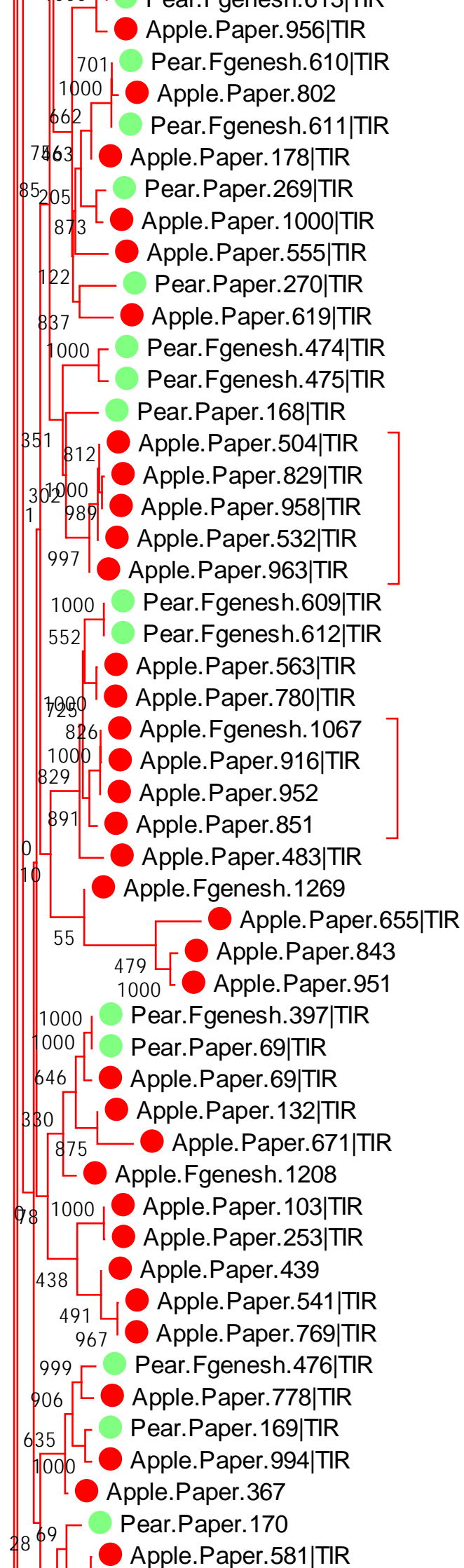

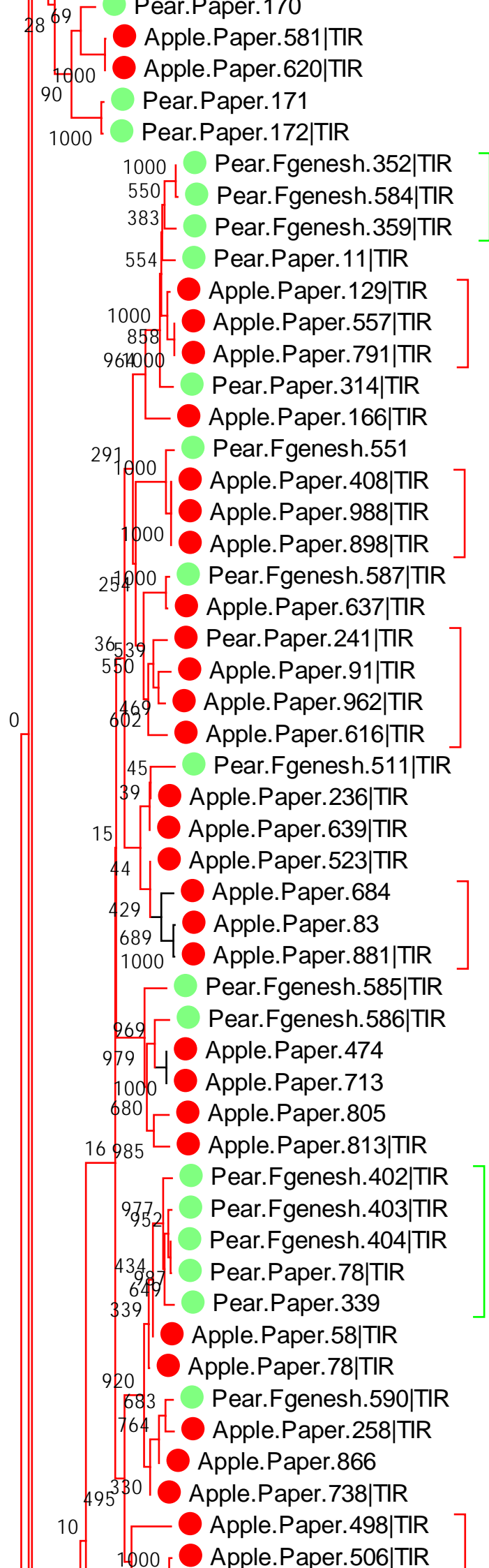

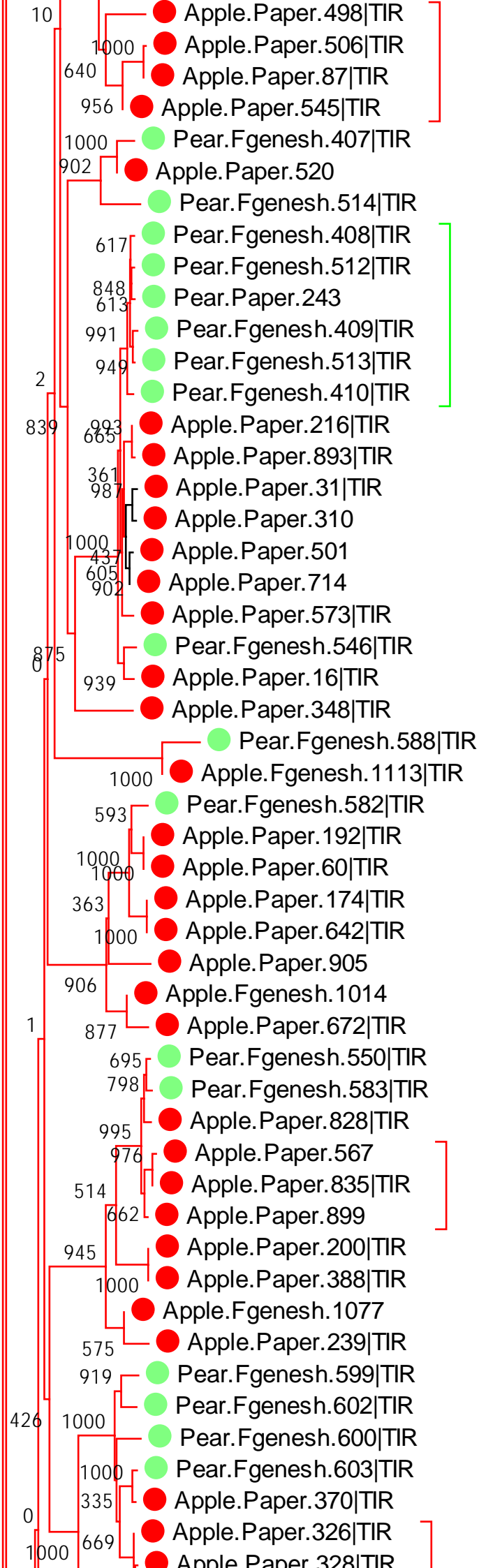

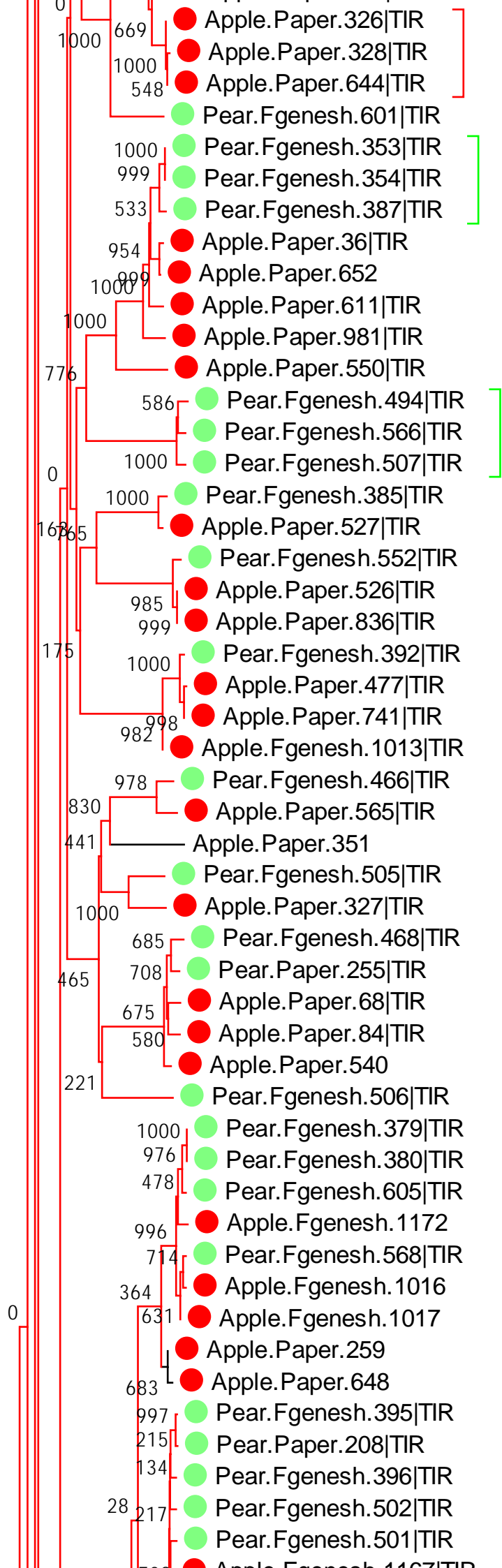

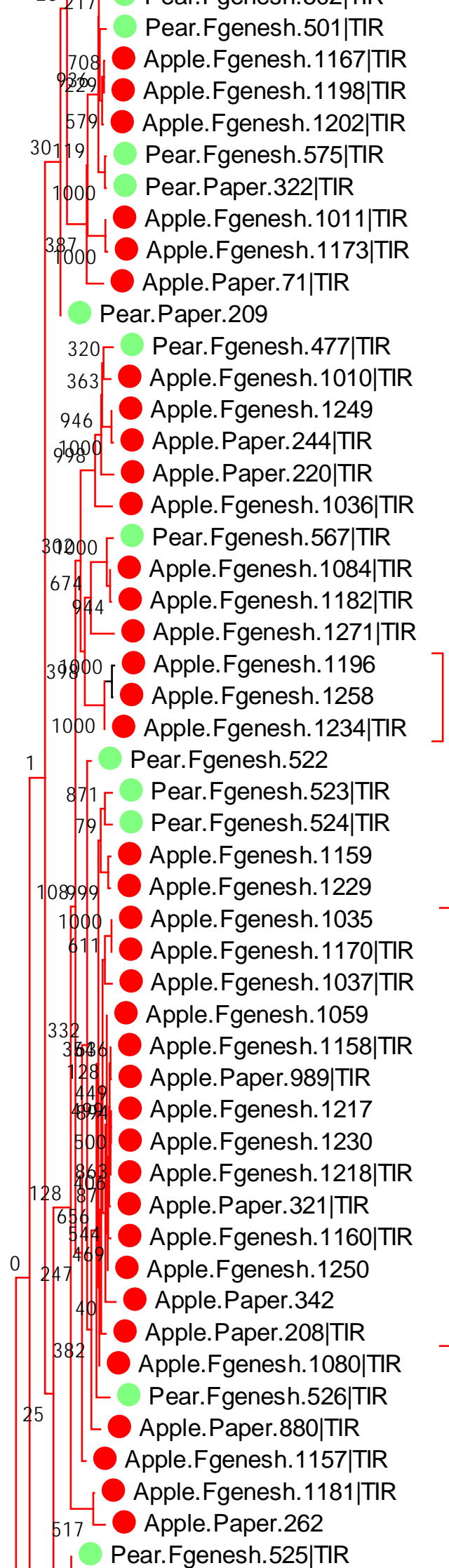

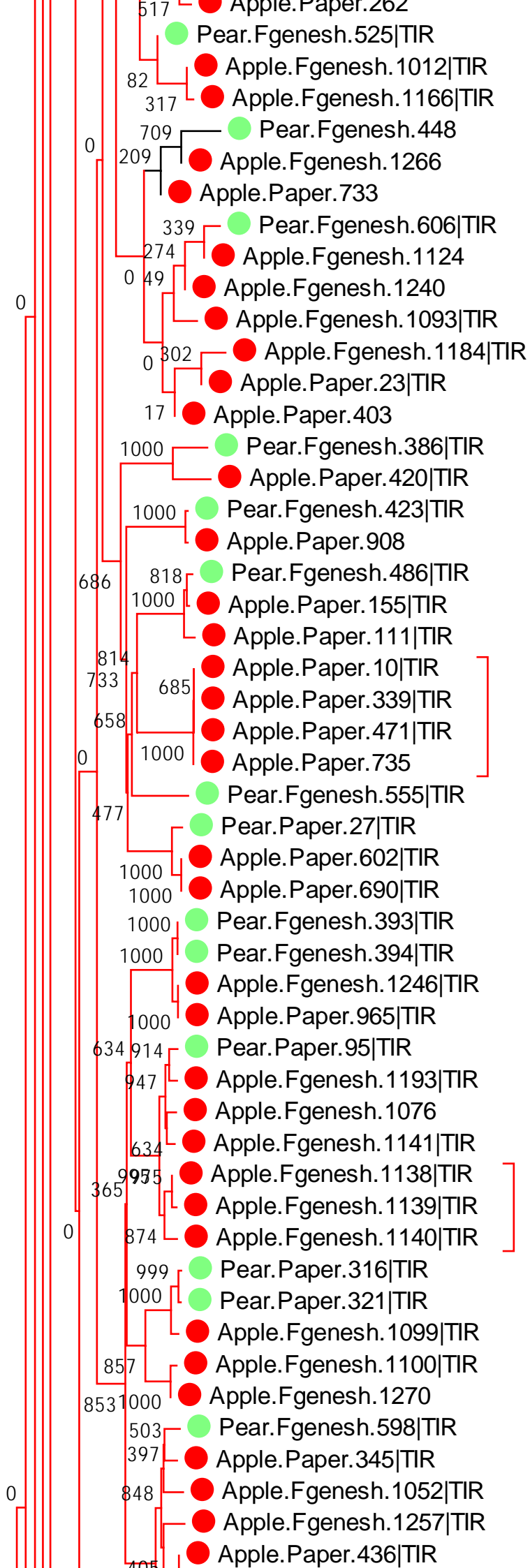

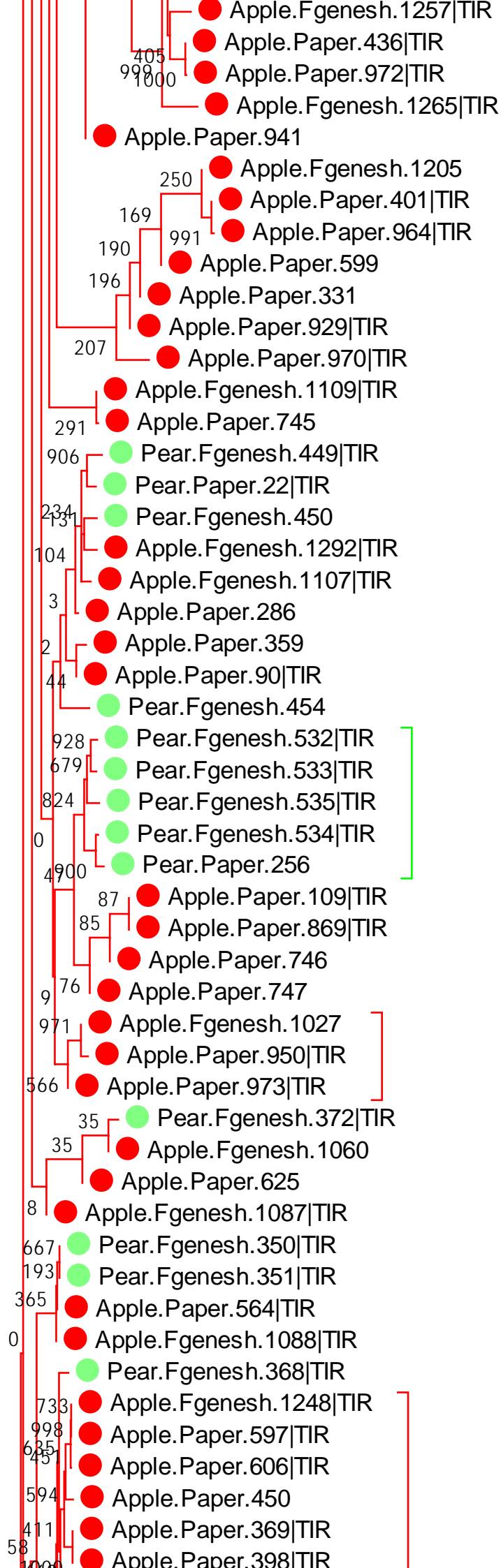

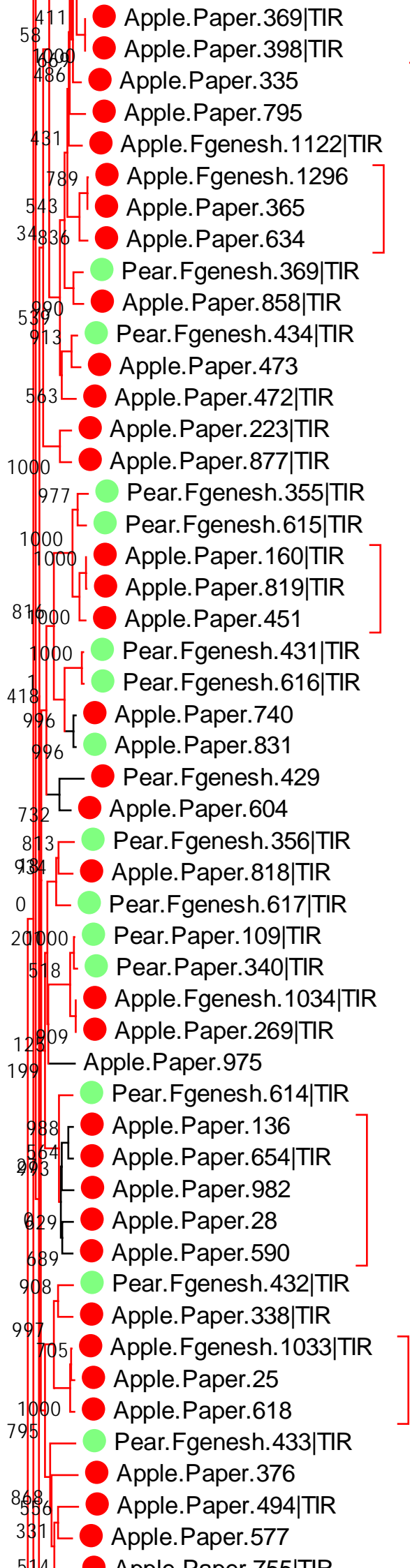

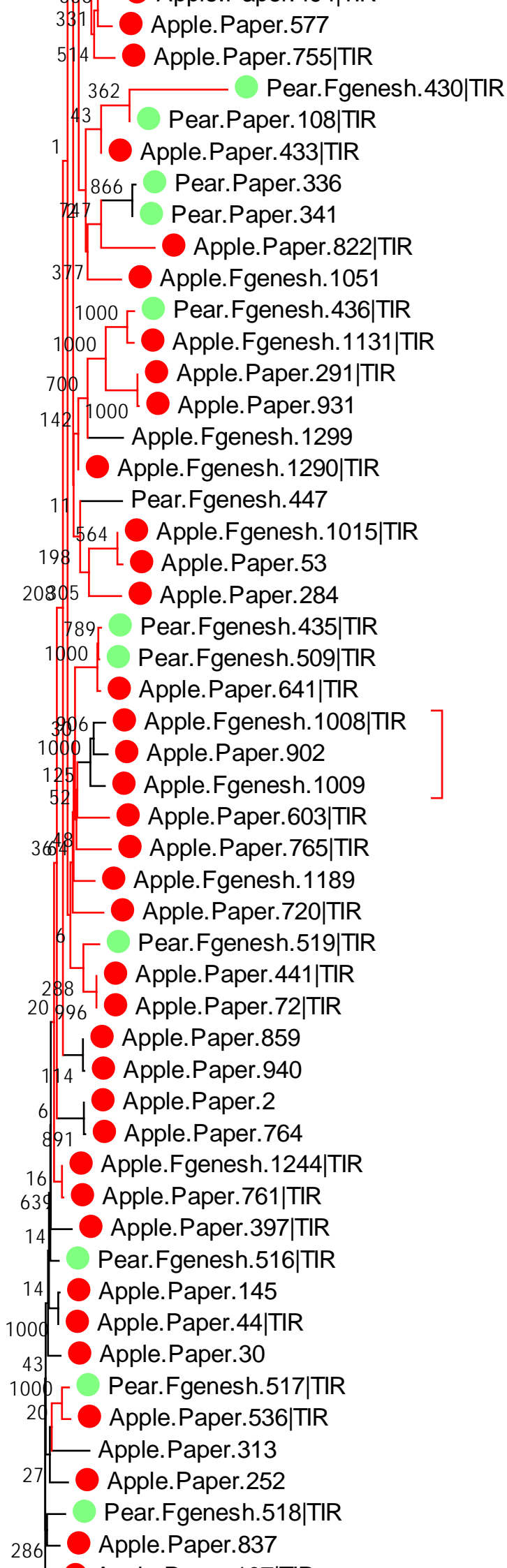

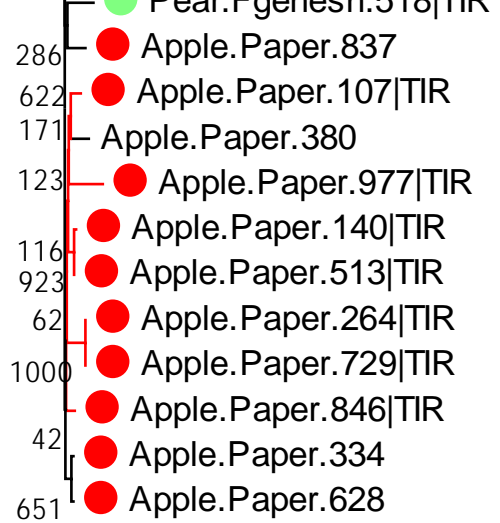

Supplement: Additional files 3: Figure S3. — Phylogenetic tree based on NBS domain of NBS-encoding genes in apple and pear. Red lines represent TIR genes and black lines represent non-TIR genes. Apple NBS genes are shown as red circles and pear NBS genes are shown as green circles. The red brackets and green brackets respectively indicate the apple-specific gene clades and pear-specific gene clades. [file 12863_2015_208_MOESM3_ESM.pdf]
