# Supplementary material for: Extreme expansion of NBS-encoding genes in Rosaceae
Source: BMC Genet. 2015 May 3;16:48. doi: 10.1186/s12863-015-0208-x (PMC4417205; doi:10.1186/s12863-015-0208-x)
Supplement: Additional files 4: Table S1. — Exon statistics in TIR and non-TIR NBS-encoding genes. [file 12863_2015_208_MOESM4_ESM.doc]

Supplementary Table 1. Exon statistics in TIR and non-TIR NBS-encoding genes.

|  | TIR-type | | | | | non_TIR-type | | | | |
| --- | --- | --- | --- | --- | --- | --- | --- | --- | --- | --- |
| Species | XN | XNL | TN | TNL | **Sum** | CN | CNL | XN | XNL | **Sum** |
| Cucumber.V1 | 2.0 | 4.3 | 3.4 | 6.2 | **4.9** | 1.3 | 1.9 | 1.2 | 1.8 | **1.7** |
| Cucumber.V2 | - | 5.3 | 3.7 | 6.2 | **5.6** | 1.0 | 2.9 | 2.0 | 1.8 | **2.3** |
| Cucumber.VW | 2.3 | 5.8 | 4.0 | 5.2 | **4.9** | 1.5 | 2.2 | 1.6 | 1.3 | **1.8** |
| Melon | 1.5 | 3.8 | 2.5 | 5.0 | **4.3** | 1.8 | 5.1 | 1.3 | 1.9 | **2.7** |
| Watermelon | - | 6.1 | 2.0 | 6.6 | **6.0** | - | 3.2 | 1.5 | 2.7 | **2.6** |
| Peach | 1.6 | 5.3 | 2.7 | 5.3 | **4.9** | 2.1 | 2.1 | 1.7 | 2.4 | **2.2** |
| Plum | 2.3 | 6.3 | 4.1 | 6.6 | **6.0** | 2.5 | 3.2 | 2.1 | 3.2 | **3.0** |
| Strawberry | 8.7 | 7.5 | 4.5 | 11.5 | **10.3** | 6.2 | 7.5 | 4.3 | 4.8 | **6.2** |
| Pear | 4.2 | 6.2 | 6.2 | 9.9 | **8.7** | 3.1 | 2.8 | 2.2 | 3.1 | **2.9** |
| Apple | 3.1 | 6.0 | 3.9 | 7.2 | **5.9** | 3.0 | 4.0 | 2.6 | 3.8 | **3.6** |
| **Average** | **3.2** | **5.7** | **3.7** | **7.0** | **6.2** | **2.5** | **3.5** | **2.1** | **2.7** | **2.9** |
